# Substituted nitrogen-bridged diazocines

**DOI:** 10.3762/bjoc.17.107

**Published:** 2021-06-25

**Authors:** Pascal Lentes, Jeremy Rudtke, Thomas Griebenow, Rainer Herges

**Affiliations:** 1 Otto Diels-Institute of Organic Chemistry, Christian Albrechts University Kiel, Otto-Hahn-Platz 4, 24118 Kiel, Germany

**Keywords:** bridged azobenzene, diazocine, photopharmacology, photoswitch, triazocine, visible light switch, water-soluble switch

## Abstract

Novel nitrogen-bridged diazocines (triazocines) were synthesized that carry a formyl or an acetyl group at the CH_2_NR-bridge and bromo- or iodo-substituents at the distant phenyl ring. The photophysical properties were investigated in acetonitrile and water. As compared to previous approaches the yields of the intramolecular azo cyclizations were increased (from ≈40 to 60%) using an oxidative approach starting from the corresponding aniline precursors. The *Z*→*E* photoconversion yields in acetonitrile are 80–85% and the thermal half-lives of the metastable *E* configurations are 31–74 min. Particularly, the high photoconversion yields (≈70%) of the water-soluble diazocines are noteworthy, which makes them promising candidates for applications in photopharmacology. The halogen substituents allow further functionalization via cross-coupling reactions.

## Introduction

Diazocines (bridged azobenzenes) are frequently used photoswitches with outstanding photophysical properties. Parent diazocine (CH_2_–CH_2_-bridged) exhibits well-separated *n–*π* transitions, which allow excellent photoconversion between the *Z* and *E* configurations ((*Z*→*E*)_385 nm_ = 92%, (*E*→*Z*)_525 nm_ > 99% in *n*-hexane) with light in the visible region [1]. Moreover, the *Z*-boat configuration is the thermodynamically stable isomer [2–9]. The latter property (i.e., the inverted stability compared to azobenzenes) makes them promising candidates for applications in photopharmacology. In the majority of azobenzene-based photopharmacophores, the bent *Z* configuration is biologically inactive [10–12]. Hence, (and in contrast to azobenzenes) the thermodynamically stable and biologically inactive *Z*-isomer can be administered and switched on with light at the site of interest with spatiotemporal resolution. Moreover, the photoconversion yield for the *E*→*Z* isomerization is quantitative (within the detection limit of UV and NMR spectroscopy). A high efficiency in switching the biological activity off is important to avoid side effects of residual concentrations of the active form [13].

**Figure 1:**
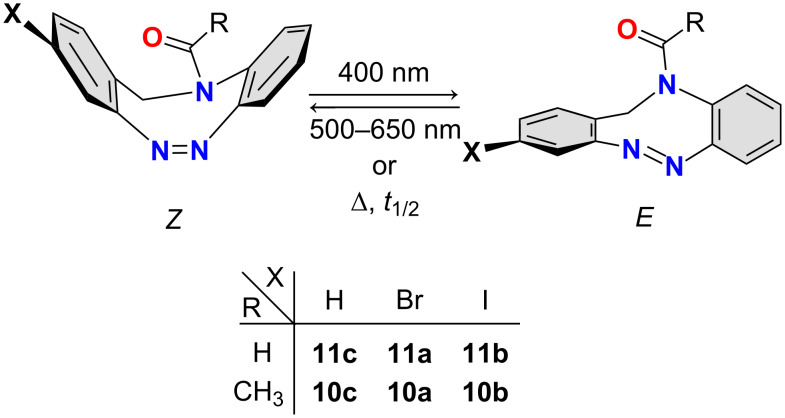
Bridged diazocines synthesized and investigated in this work.

**Scheme 1:**
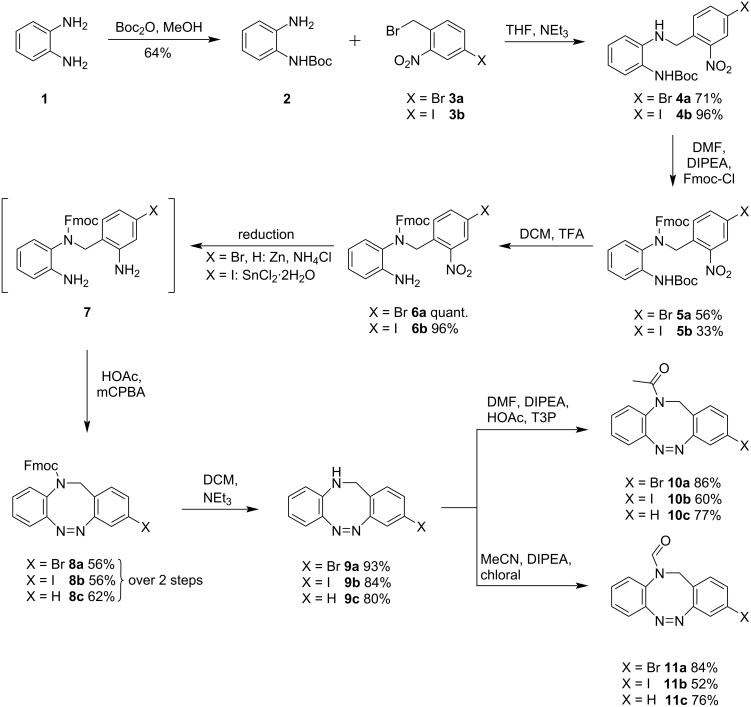
Synthesis of 3-bromo- and 3-iodo-acetylated CH_2_NR diazocines **10** (R = Ac) and formylated diazocines **11** (R = CHO).

Water solubility and high *Z*→*E* switching efficiencies in water are additional important criteria for applications in biological environments [14]. Our previously published NAc-bridged diazocine **10c** (Scheme 1, Table 1) exhibits both properties [15]. This is in stark contrast to the CH_2_–CH_2_ and S–CH_2_-bridged diazocines and the majority of azobenzenes [9,16–20]. Spurred by the promising properties of CH_2_–NR-bridged diazocines (triazocines), we now explored this class of photoswitches and developed synthetic access to these photochromes (Figure 1).

## Results and Discussion

### Synthesis

The first three stages of the synthesis of CH_2_–NR-bridged diazocines are analogous to the previously described synthesis of CH_2_–NH-bridged diazocine [15]. The single Boc-protected 1,2-phenylenediamine (**2**, Scheme 1) is reacted with halogen-substituted 2-nitrobenzyl bromides **3** [21] forming *N*-benzylanilines **4**, which were protected with Fmoc chloride to accomplish an orthogonal protective group strategy. The removal of the Boc groups from compounds **5** with TFA gave the mixed aniline and nitro precursors **6**.

In previous approaches, the nitro groups were reduced to hydroxylamines with zinc and oxidized to the corresponding nitroso compounds with iron(III) to perform an intramolecular Baeyer–Mills reaction [15,21]. We found that a complete reduction of the nitro group to aniline **7** and oxidation with mCPBA is increasing the yield of the intramolecular cyclization from 39% to 62% (over two steps) for the unsubstituted diazocine **8c** as compared to the pathway via the hydroxylamine. The 3-bromo **8a** and 3-iodo **8b** compounds were obtained in 56% yield using the oxidative method of Trauner [22] with mCPBA. The Fmoc groups were removed with NEt_3_ to yield the NH-diazocines **9**. The acetylated diazocines **10a**–**c** were synthesized using a mixed anhydride of acetic acid and T3P (propanephosphonic acid anhydride). The formylation of NH-diazocines **9a**–**c** was accomplished with chloral [23] under non-acidic conditions.

### Investigation of the photophysical properties

**Figure 2:**
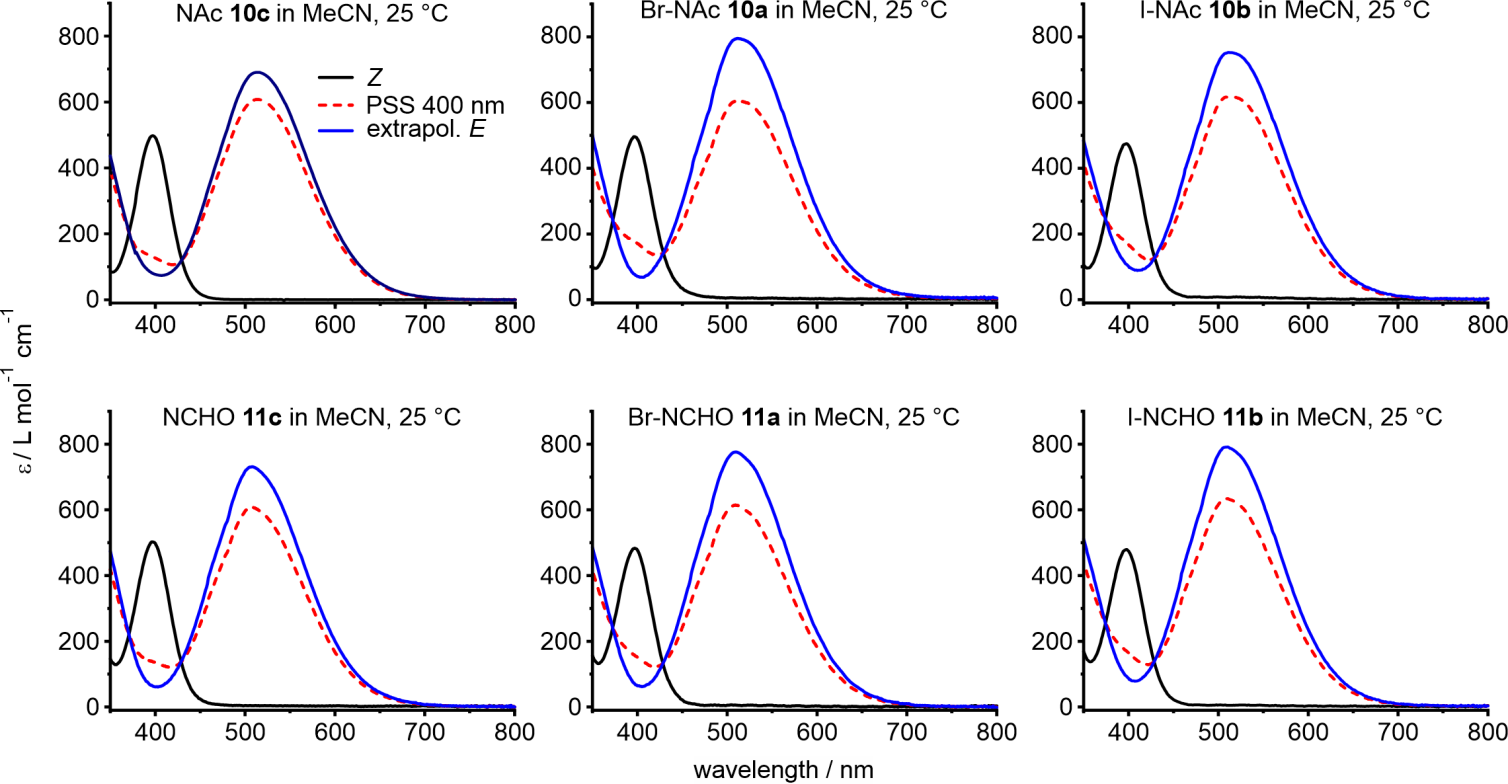
UV–vis spectra of 3-bromo and 3-iodo, and unsubstituted CH_2_NAc-bridged (**10a**–**c**) and CH_2_NCHO-bridged (**11a**–**c**) diazocines. The spectra of *Z*-isomers are given in black, the photostationary states at 400 nm are represented as dashed red lines and the extrapolated spectra of the pure *E*-isomers are in blue.

The UV–vis spectra of diazocines **10a**–**c**, and **11a**–**c** were recorded in acetonitrile at 25 °C. All compounds exhibit an *n–*π* transition at about 400 nm (*Z*→*E* conversion) and an *n–*π* transition at about 520 nm (*E*→*Z* conversion, Figure 2, Table 1).

**Table 1:** Photophysical properties of *N*-diazocines **10a**–**c** and **11a**–**c** in acetonitrile.

	acetonitrile							

	λ_max(Z)_	λ_max(E)_	ε_λmax(Z)_	ε_λmax(E)_	Γ_Z→E_^a^	*t*_1/2_ (25 °C)	*Ε*_Α_	ln(A)
	nm		L mol^−1^ cm^−1^		%	min	kJ mol^−1^	

Br-NAc **10a**	397	515	495	791	81	30.9	93.4	29.8
I-NAc **10b**	397	517	480	778	82	28.6	87.0	27.3
NCHO **11c**	397	509	502	760	85	74.0	88.4	26.9
Br-NCHO **11a**	397	509	469	784	82	49.9	93.9	29.5
I-NCHO **11b**	398	511	483	798	80	48.1	90.9	28.3
NAc **10c**	397	513	495	759	88	29.5	87.6	27.5

^a^Extrapolated values (for details, see Supporting Information File 1, section IV).

Irradiation with 400 nm gives the metastable *E*-isomers of the acetylated and formylated derivatives **10** and **11** with good photoconversion yields (Γ) of 80–85% (Table 1) in acetonitrile. A complete *E*→*Z* conversion (>99%) can be achieved with light between 520 and 600 nm. The unsubstituted acetylated and formylated diazocines **10c** and **11c** exhibit similar conversion yields (88% and 85%) and halogenation as well does not have a significant influence. However, thermal half-lives (*t*_1/2_) of the metastable *E*-isomers of the 3-bromo and 3-iodo *N*-acetyl diazocines **10a** and **10b** (≈30 min) are significantly smaller than the half-lives of the corresponding bromo and iodo *N*-formyl derivatives **11a** and **11b** (≈50 min). In general, halogenation decreases the half-lives compared to unsubstituted diazocines **10c** and **11c**. The activation barrier (*E*_A_) of the *E*→*Z* isomerization (obtained by an Arrhenius plot) is higher in formylated compounds **11** compared to acetylated compounds **10** and is further increased by halogenation.

**Figure 3:**
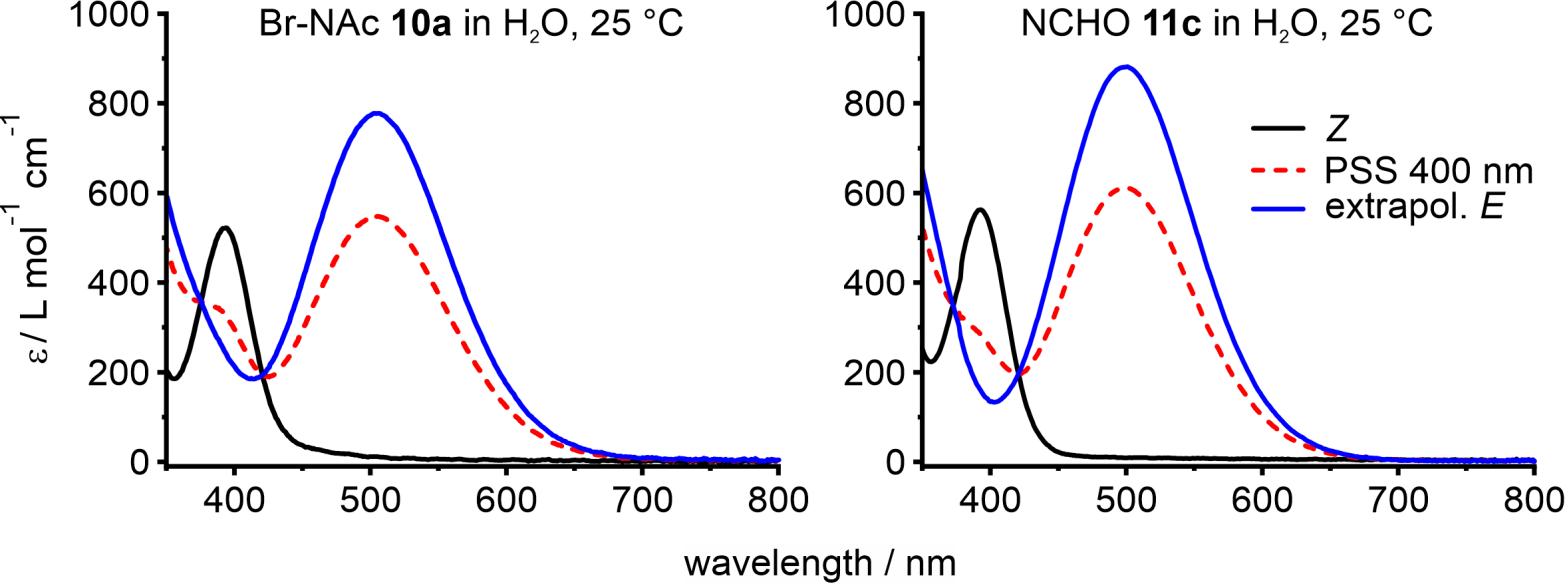
UV–vis spectra of 3-bromo-*N*Ac-diazocine **10a** and *N*-formyl-diazocine **11c** in water. Spectra of *Z*-isomers (black curve), the photostationary states at 400 nm (dashed red line), and the extrapolated spectra of the pure *E*-isomers (blue).

The unsubstituted *N*-formyl diazocine **11c** and brominated *N*Ac-diazocine **10a** were also investigated in pure water since they are water-soluble (**11c**: ≈250 µM, **10a**: ≈150 µM). The highest *Z*→*E* conversion yields are observed by irradiation with 400 nm in water and the back-isomerization *E*→*Z* can be accomplished by irradiation with light in the range of 525 and 600 nm (Figure 3, Table 2).

**Table 2:** Photophysical properties of water-soluble *N*-diazocines **10a**, **10c**, and **11c** in H_2_O.

	H_2_O							

	λ_max(Z)_	λ_max(E)_	ε_λmax(Z)_	ε_λmax(E)_	Γ_Z→E_^a^	*t*_1/2_ (25 °C)	*Ε*_Α_	ln(A)
	nm		L mol^−1^ cm^−1^		%	min	kJ mol^−1^	

Br-NAc **10a**	394	502	534	975	70	69.6	99.9	31.6
NCHO **11c**	393	500	567	871	69	198	97.8	29.7
NAc **10c**	393	502	564	850	72	72.8	90.4	27.7

^a^Extrapolated values (for details, see Supporting Information File 1, section IV).

The photoconversion yields (*Z*→*E*) of *N*-formyl diazocine **11c** in water and bromo-*N*Ac diazocine **10a** are about 70%, which do not differ significantly from unsubstituted NAc diazocine **10c** (72%) [15]. It is interesting to note that the half-lives and activation barriers (*E*→*Z*) are increasing (*t*_1/2_ ≈ 2–2.5-fold) in water as compared to the less polar acetonitrile.

## Conclusion

Five nitrogen-bridged diazocines (triazocines) were synthesized and characterized. Formyl (R = CHO) and acetyl groups (R = Ac) were introduced at the CH_2_NR bridge and the distant phenyl rings are Br and I substituted. In contrast to previous approaches, the azo cyclization (ring closure) was achieved via the oxidation of the bis-anilines **7** with *m*CPBA (≈60% yield). Among the nitrogen-bridged diazocines compounds **10a** and **11c** are water soluble and retained their high switching efficiency (≈70%) also in water. The half-lives of the metastable *E*-isomers are larger for the *N*-formyl diazocines **11a**–**c** compared to the acetylated compounds **10a–c** and generally, the half-lives are larger in water than in acetonitrile. Halogen atoms Br and I at the phenyl rings in 3-position as in **10a**,**b**, and **11a**,**b** are a good starting point for further functionalization [17,20,24]. We conclude that CH_2_NAc and CH_2_NCHO bridged diazocines (triazocines) are promising candidates for applications in biological environments and particularly as photoswitches in light-activatable drugs.

## Supporting Information

File 1 Analytical equipment, experimental procedures, NMR and UV–vis spectra.
