# Supplementary material for: Substituted nitrogen-bridged diazocines
Source: Beilstein J Org Chem. 2021 Jun 25;17:1503–8. doi: 10.3762/bjoc.17.107 (PMC8239257; doi:10.3762/bjoc.17.107)
Supplement: File 1 — Analytical equipment, experimental procedures, NMR and UV–vis spectra. [file Beilstein_J_Org_Chem-17-1503-s001.pdf]

BEILSTEIN JOURNAL OF ORGANIC CHEMISTRY

Supporting Information

for

Substituted nitrogen-bridged diazocines

Pascal Lentes, Jeremy Rudtke, Thomas Griebenow and Rainer Herges

Beilstein J. Org. Chem. **2021,** *17,* 1503–1508. doi:10.3762/bjoc.17.107

Analytical equipment, experimental procedures, NMR and UV–vis spectra

License and Terms: This is a supporting information file under the terms of the Creative Commons Attribution License (https://creativecommons.org/licenses/by/4.0). Please note that the reuse, redistribution and reproduction in particular requires that the author(s) and source are credited and that individual graphics may be subject to special legal provisions.
The license is subject to the *Beilstein Journal of Organic Chemistry* terms and conditions: (https://www.beilstein-journals.org/bjoc/terms)

Table of contents

I. Analytical equipment S2

II. Syntheses S3–S30

III. UV–vis switching experiments S32–S44

IV. ^{1}H NMR switching experiments S44–S53

I. Analytical equipment

NMR spectroscopy

NMR spectra were measured in deuterated solvents (Deutero). The spectra were referenced to the following solvent residual signals:

solvent	degree of deuteration	^{1}H signal (ppm)	^{13}C signal (ppm)
acetone-d_6	99.8 %	2.05 (quintet)	29.84 (septet)
chloroform-d_1	99.8 %	7.26 (singlet)	77.16 (triplet)
CD_3CN	99.5 %	1.94 (quintet)	/
water-d_2	99.9 %	4.79 (singlet)	/

Spectra were recorded with a Bruker AC 200 (^{1}H NMR: 200 MHz), a Bruker DRX 500 (^{1}H NMR: 500 MHz, ^{13}C NMR: 125 MHz), and a Bruker AV 600 (^{1}H NMR: 600 MHz, ^{13}C NMR: 150 MHz). The multiplicities of the signals were abbreviated with s (singlet), d (doublet), t (triplet), q (quartet), quint (quintet), m (multiplet) and br. (broad) in addition for broad signals.

Melting point

Melting points were measured with a Melting Point B-540 (Büchi) in melting point tubes without further correction.

Mass spectrometry

Mass spectra (EI) and high-resolution mass spectra (HR-EI) were measured with an AccuTOF GCv 4G (Joel) with an ionization energy of 70 eV. HR-ESI mass spectra were measured with a Q Exactive Plus (Thermo Scientific).

IR spectroscopy

Infrared spectra were measured with a Perkin-Elmer 1600 Series FT-IR spectrometer with an A531-G Golden-Gate-Diamond-ATR-unit. Signals were abbreviated with w (weak), m (medium), s (strong) for its intensity.

Chromatography

Silica gel (Merck, particle size 0.040-0.063 mm) was used for column chromatography purifications. R_f values were determined by thin layer chromatography on Polygeram® SilG/UV254 (Macherey Nagel, 0.2 mm particle size).

UV–vis spectroscopy

UV–vis spectra were measured with a Lambda 650 spectrometer (Perkin-Elmer) and quartz cuvettes of 10 mm optical path length were used.

Chemicals

All commercially available chemicals and solvents were used without purification.

Light sources

For exposure experiments with 400 nm, a custom-built LED light source was used. The irradiation wavelength of the 400 nm LED light source was measured via a mobile UV–vis spectrometer (USB4000-UV-vis, Ocean Optics, Largo, FL, USA).

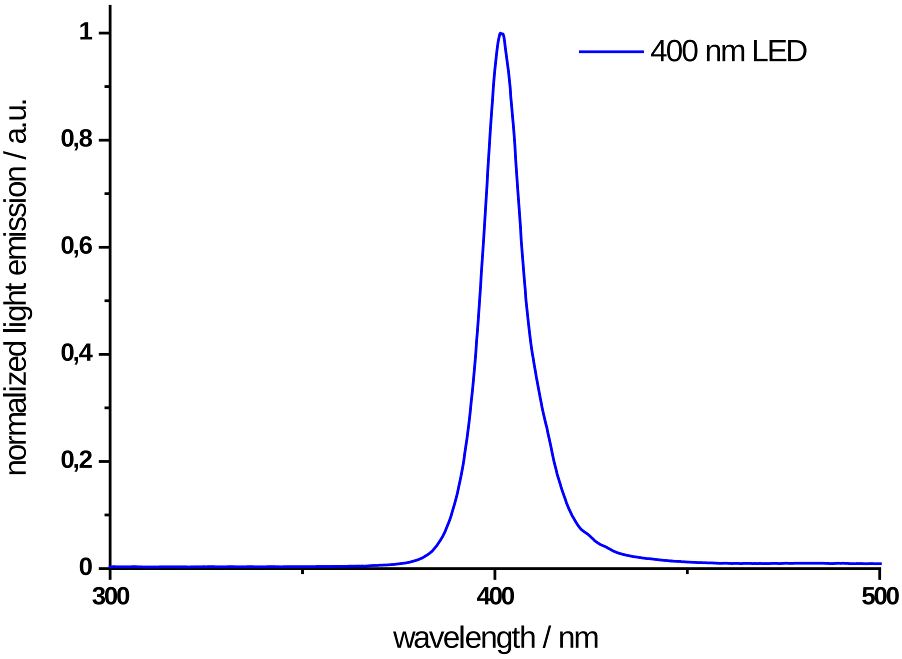

Figure S1: Emission spectrum of 400 nm LED light source. The full width at half maximum (FWHM) amounts to 12 nm (396–401 nm, maximum at 401 nm).

II. Syntheses

II.1 Synthesis of (9*H*-fluorene-9-yl)methyl (2-aminobenzyl)(2-aminophenyl)carbamate

(9*H*-Fluorene-9-yl)methyl (2-aminophenyl)(2-nitrobenzyl)carbamate[1] (6.80 g, 14.6 mmol) was dissolved in 500 mL of ethanol and 250 mL of a 1 M NH_4Cl solution in water was added. It was heated to 75 °C and zink powder (7.64 g, 117 mmol, 8 equiv) was added. The reaction mixture was stirred at 75 °C for 1 h and then hot filtrated. The solvent was evaporated and to the residue were added 500 mL of DCM and 500 mL of deionized water. The organic layer was separated and extracted twice with DCM. The combined organic layers were dried over $MgSO_4$ and the solvent was evaporated. The crude product, a white powder (6.36 g, 14.6 mmol, quant.) was used without further purification.

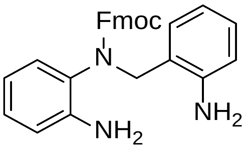

melting point: 75.7 °C

^{1}H NMR (600 MHz, acetone-d$_6$, 298 K): δ = 7.79 (d, ^3J = 6.9 Hz, 1 H, Ar-*H*), 7.54-6.31 (m, 14 H, Ar-*H*), 5.04-3.95 (m, 5 H, aliph. *H*) ppm.

^{13}C NMR (150 MHz, acetone-d$_6$, 298 K): δ = 131.28, 130.86, 130.47, 130.27, 129.76, 129.32, 128.77, 128.47, 128.44, 127.88, 127.85, 127.81, 126.60, 126.45, 126.25, 124.11, 123.92, 123.75, 121.30, 121.15, 120.69, 120.63, 119.96, 119.78, 117.64, 117.24, 116.65, 115.90, 68.71, 68.34, 67.87, 50.66, 49.63, 49.38, 47.96, 47.84 ppm.

IR (ATR): $\tilde{\nu}$ = 3353 (w), 3038 (w), 1618 (s), 1621 (m), 1501 (m), 1450 (m), 1409 (s), 1356 (w), 1310 (s), 1276 (s), 1215 (w), 1128 (m), 1069 (w), 1007 (w), 979 (m), 939 (w), 860 (w), 739 (s), 631 (m), 621 (w), 602 (w), 552 (w), 525 (m) cm^{-1}.

MS (ESI HR, CHCl$_3$/MeOH): *m/z* ($C_{28}H_{25}N_3O_2+H^+$) = calc.: 436.20195, found: 436.20159 ± 0.84 ppm.

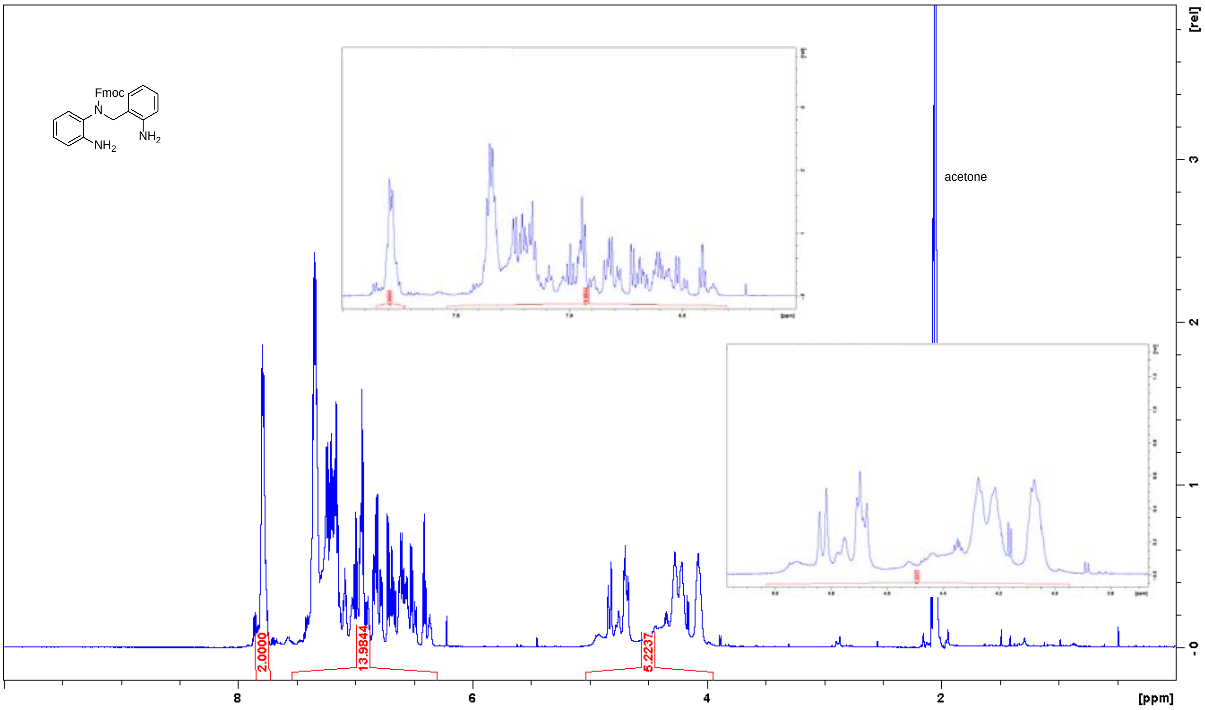

Figure S2: ^{1}H NMR spectrum of the given aniline compound.

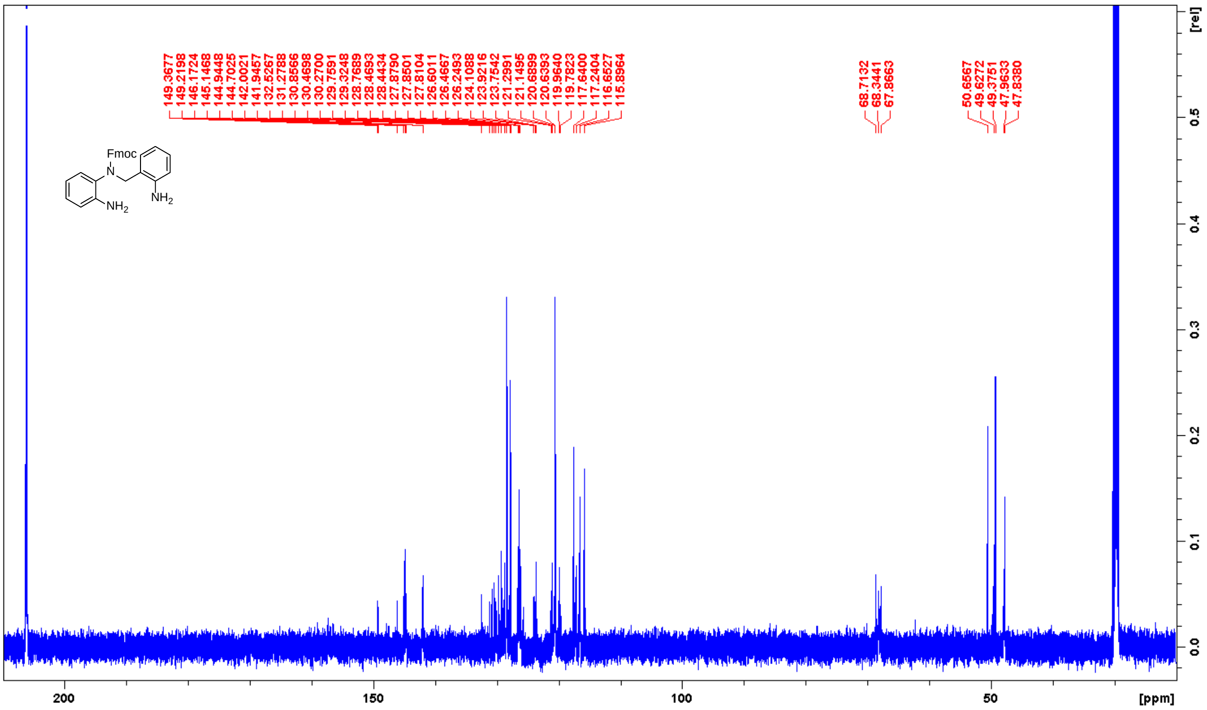

Figure S3: ^{13}C NMR spectrum of the given aniline compound.

II.2 Synthesis of (9*H*-fluorene-9-yl)methyl (*Z*)-dibenzo[*c*,*g*][1,2,5]triazocine-11(12*H*)-carboxylate (8c)

(9*H*-Fluorene-9-yl)methyl (2-aminobenzyl)(2-aminophenyl)carbamate (3.70 g, 8.50 mmol) was dissolved in 250 mL of acetic acid and *m*CPBA (3.81 g, 17.0 mmol, ≥ 77%) in 250 mL of acetic acid was added dropwise over a period of 2 h. The reaction mixture was stirred at rt over night and after that, the solvent was evaporated. To the residue were added 100 mL of DCM and 250 mL of half-concentrated $NaHCO_3$ solution in deionized water and the organic layer was separated. It was extracted twice with DCM and the combined organic layers were dried over $MgSO_4$. The solvent was evaporated and flash column chromatography on silica (0.040–0.063 mm, ethyl acetate/cyclohexane 1:3, R_f = 0.38) gave a yellow solid (**8c**, 2.28 g, 5.28 mmol, 62%).

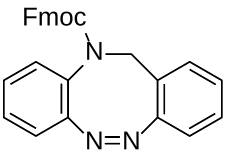

^{1}H NMR (200 MHz, acetone-d$_6$, 300 K): δ = 7.83 (d, ^{3}J = 7.2 Hz, 2 H, Ar-*H*), 7.73-6.73 (m 14 H, Ar-*H*), 5.12-3.94 (m, 5 H, aliph. *H*) ppm.

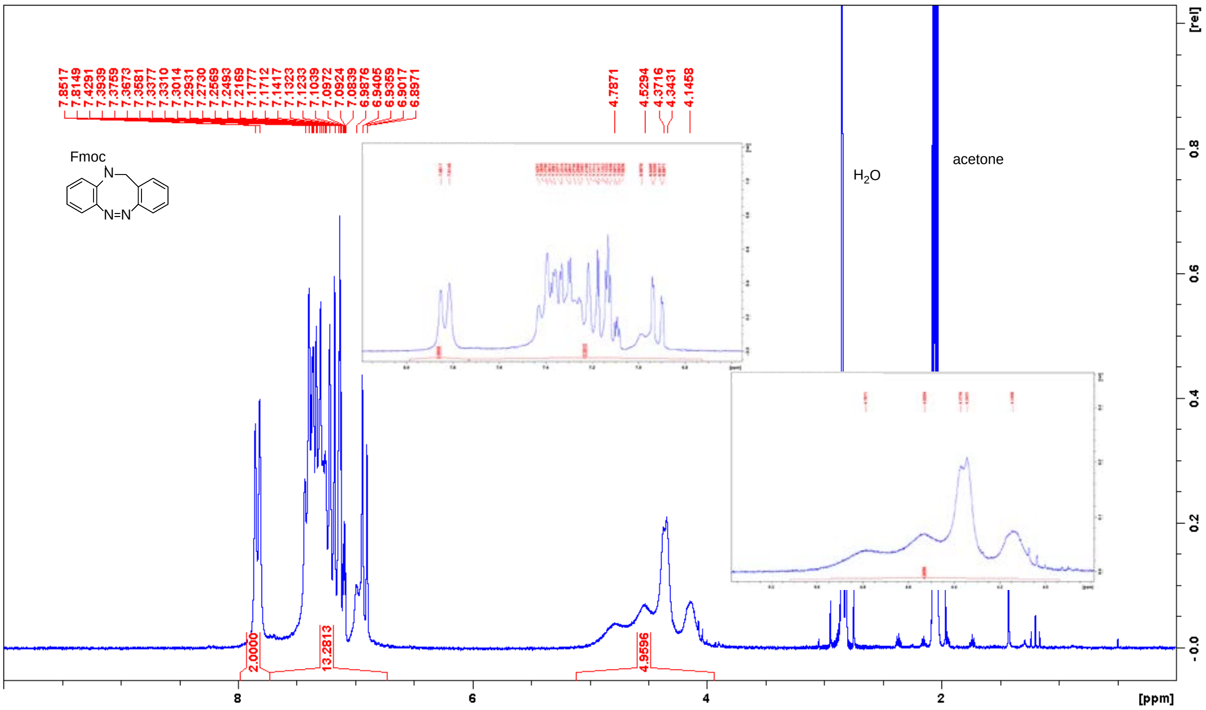

Figure S4: ^{1}H NMR spectrum of compound **8c**.

II.3 Synthesis of chloral[2]

Under a nitrogen atmosphere, 5 mL of concentrated H_2SO_4 was added to chloral hydrate (10.0 g, 60.5 mmol) and chloral (6.31 g, 42.8 mmol, 71%) was directly distilled out of the reaction mixture with a vigreux condenser.

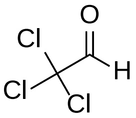

boiling point: 98 °C

^{1}H NMR (200 MHz, $CDCl_3$, 300 K): δ = 9.06 (s, 1 H, C*H*O) ppm.

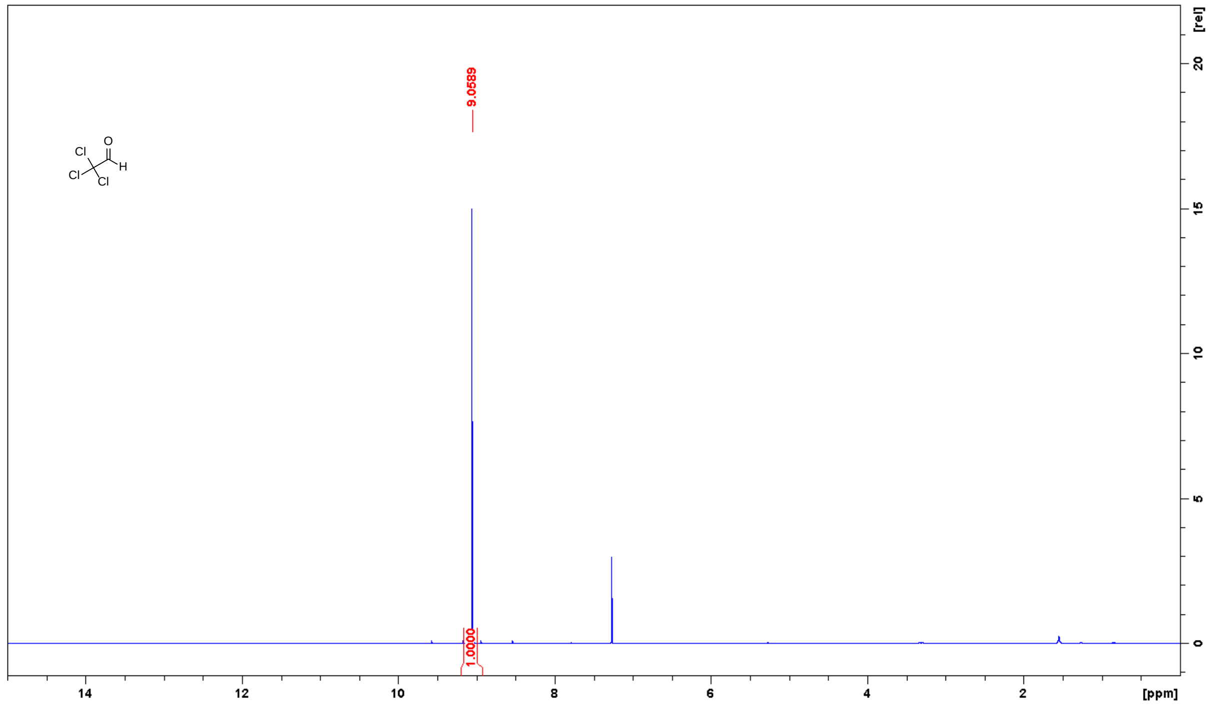

Figure S5: ^{1}H NMR spectrum of chloral.

II.4 Synthesis of (*Z*)-dibenzo[*c*,*g*][1,2,5]triazocine-11(12*H*)-carbaldehyde (11c)

Under a nitrogen atmosphere, (*Z*)-11,12-dihydrodibenzo[*c*,*g*][1,2,5]triazocine[1] **9c** (100 mg, 478 µmol) was dissolved in MeCN (10 mL anhydrous), and DIPEA (832 µL, 4.78 mmol) and chloral (466 µL, 4.78 mmol) were added. The reaction mixture was stirred at rt for 21 h. The solvent was removed and separation of the products by flash column chromatography on silica (0.040-0.063 mm, ethyl acetate/cyclohexane 1:2, R_f = 0.33) gave a pale yellow solid (**11c**, 86 mg, 362 µmol, 76%).

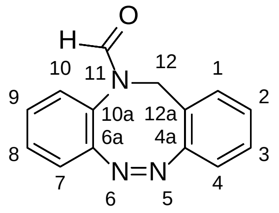

melting point: 206.5 °C

^{1}H NMR (500 MHz, acetone-d$_6$, 298 K): δ = 8.38 (s, 1 H, C*H*O), 7.38 (td, ^{3}J = 7.7 Hz, ^{4}J = 1.3 Hz, 1 H, *H*-2), 7.34-7.28 (m, 2 H, *H*-1, *H*-3), 7.24-7.17 (m, 2 H, *H*-8, *H*-9), 7.15 (dd, ^{3}J = 7.9 Hz, ^{4}J = 1.2 Hz, 1 H, *H*-4), 7.01 (dd, ^{3}J = 7.9 Hz, ^{4}J = 1.1 Hz, 1 H, *H*-7/10), 6.91 (dd, ^{3}J = 7.8 Hz, ^{4}J = 1.5 Hz, 1 H, *H*-7/10), 4.52 (m$_c$, 2 H, CH_2) ppm.

^{13}C NMR (125 MHz, acetone-d$_6$, 298 K): δ = 162.27 (*C*=O), 157.61 (*C*-12a), 152.96 (*C*-4a), 131.38 (*C*-1), 130.31 (*C*-2), 129.10 (*C*-8/9), 128.98 (*C*-8/9), 128.73 (*C*-3), 128.06 (*C*-4), 123.15 (*C*-6a/10a), 120.46 (*C*-7/10), 119.30 (*C*-7/10), 49.47 (CH_2) ppm.

IR (ATR): $\tilde{\nu}$=2251 (w), 1662 (s), 1587 (w), 1514 (w), 1476 (m), 1439 (w), 1367 (w), 1309 (s), 1273 (m), 1242 (m), 1152 (w), 1040 (w), 1015 (w), 944 (w), 916 (w), 857 (w), 804 (w), 774 (s), 755 (s), 727 (s), 687 (m), 661 (w),631 (w), 596 (w), 550 (s), 538 (m) cm^{-1}.

MS (ESI HR, $CHCl_3$/MeOH): *m/z* ($C_{14}H_{11}{}^{79}BrN_3O+H^+$) = calc.: 238.09749, found: 238.09758 ± 0.39 ppm.

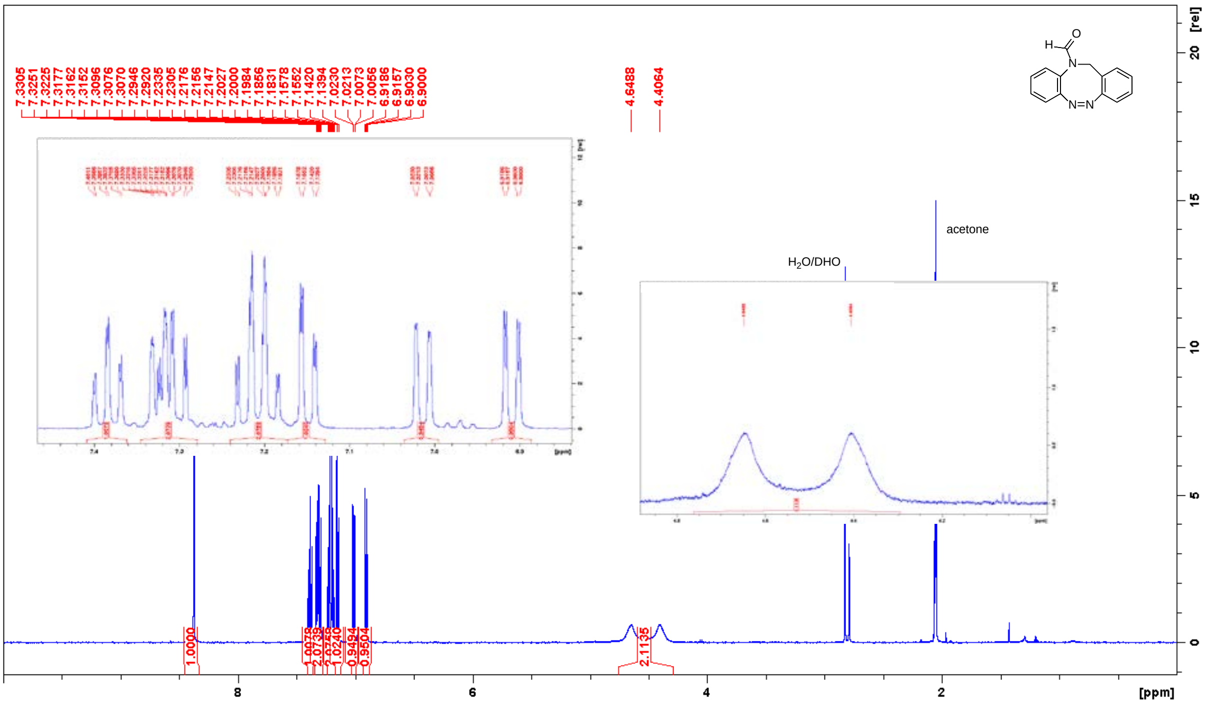

Figure S6: ^{1}H NMR spectrum of compound **11c**.

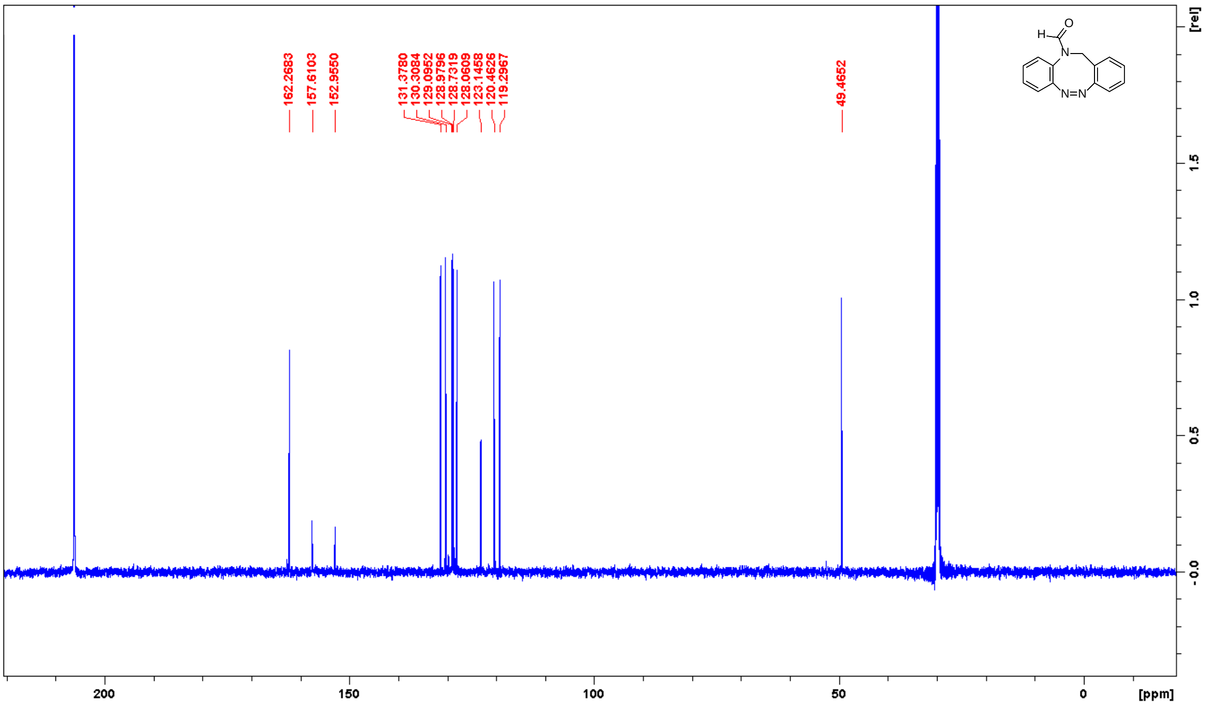

Figure S7: ^{13}C NMR spectrum of compound **11c**.

II.5 Synthesis of *tert*-butyl (2-((4-bromo-2-nitrobenzyl)amino)phenyl)carbamate (4a)

Under a nitrogen atmosphere, a solution of *tert*-butyl (2-aminophenyl)carbamate[1] (**2**, 2.62 g, 12.6 mmol) in 50 mL of abs. THF was prepared, and then triethylamine (4.30 mL, 13.5 mmol) and 4-bromo-1-(bromomethyl)-2-nitrobenzene[3] (**3a**, 3.72 g, 12.6 mmol) was added. The reaction mixture was refluxed

for 20 h and after that, the solvent was evaporated. Then, 50 mL of water and 50 mL of DCM were added to the residue. The organic layer was separated and it was extracted twice with 50 mL of DCM. The combined organic layers were dried over $MgSO_4$ and the solvent was evaporated. Recrystallization from cyclohexane/ethyl acetate 1:1 gave a yellow solid (**4a**, 3.03 g, 8.85 mmol, 71%).

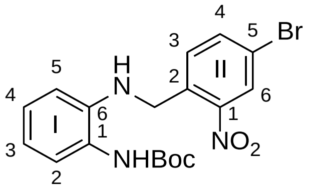

melting point: 151.1 °C

^{1}H NMR (500 MHz, acetone-d$_6$, 298 K): δ = 8.24 (d, ^4J = 2.0 Hz, 1 H, Ar^{II}-*H*-6), 7.83 (dd, ^3J = 8.4 Hz, ^4J = 2.0 Hz, 1 H, Ar^{II}-*H*-4), 7.73 (d, ^3J = 8.4 Hz, 1 H, Ar^{II}-*H*-3), 7.63 (s, 1 H, N*H*), 7.25 (d, ^3J = 7.6 Hz, 1 H, Ar^{I}-*H*-2), 6.93 (td, ^3J = 7.6 Hz, ^4J = 1.5 Hz, 1 H, Ar^{I}-*H*-4), 6.64 (td, ^3J = 7.6 Hz, ^4J = 1.3 Hz, 1 H, Ar^{I}-*H*-3), 6.50 (dd, ^3J = 7.6 Hz, ^4J = 1.1 Hz, 1 H, Ar^{I}-*H*-5), 5.59-5.42 (m, 1 H, N*H*), 4.76 (d, ^3J = 6.2 Hz, 2 H, CH_2), 1.47 (s, 9H, CH_3) ppm.

^{13}C NMR (125 MHz, acetone-d$_6$, 298 K): δ = 155.13 (*C*=O), 150.02 (Ar^{II}-*C*-1), 142.84 (Ar^{I}-*C*-6), 137.22 (Ar^{II}-*C*-4), 136.28 (Ar^{II}-*C*-2), 132.44 (Ar^{II}-*C*-3), 128.32 (Ar^{II}-*C*-6), 127.11 (Ar^{I}-*C*-4), 126.87 (Ar^{I}-*C*-2), 125.50 (Ar^{I}-*C*-1), 120.93 (Ar^{II}-*C*-5), 118.02 (Ar^{I}-*C*-3), 112.35 (Ar^{I}-*C*-5), 79.90 (*C*-(CH_3)$_3$), 45.09 (-CH_2), 28.58 (-*C*H_3) ppm.

IR (ATR): $\tilde{\nu}$ = 3400 (m), 3270 (w), 2976 (w), 1686 (m), 1604 (m), 1528 (s), 1462 (m), 1348 (w), 1151(s) 830 (m), 753 (m) cm^{-1}.

MS (EI, 70 eV): *m/z* (%) = 421.06 (3) [M]$^+$, 365.00 (13) [$C_{14}H_{12}BrN_3O_4$]$^+$, 321.01 (9) [$C_{13}H_{12}BrN_3O_2$]$^+$, 286.99 (18) [$C_{13}H_{10}BrN_3$]$^+$, 197.95 (14) [$C_7H_7BrN_2$]$^+$, 119.06 (32) [$C_7H_7N_2$]$^+$, 107.06 (32) [$C_6H_7N_2$]$^+$.

MS (HR) (EI, 70 eV): *m/z* ($C_{18}H_{20}{}^{79}BrN_3O_4$) = calc.: 421.06372, found: 421.06392 ± 0.49 ppm.

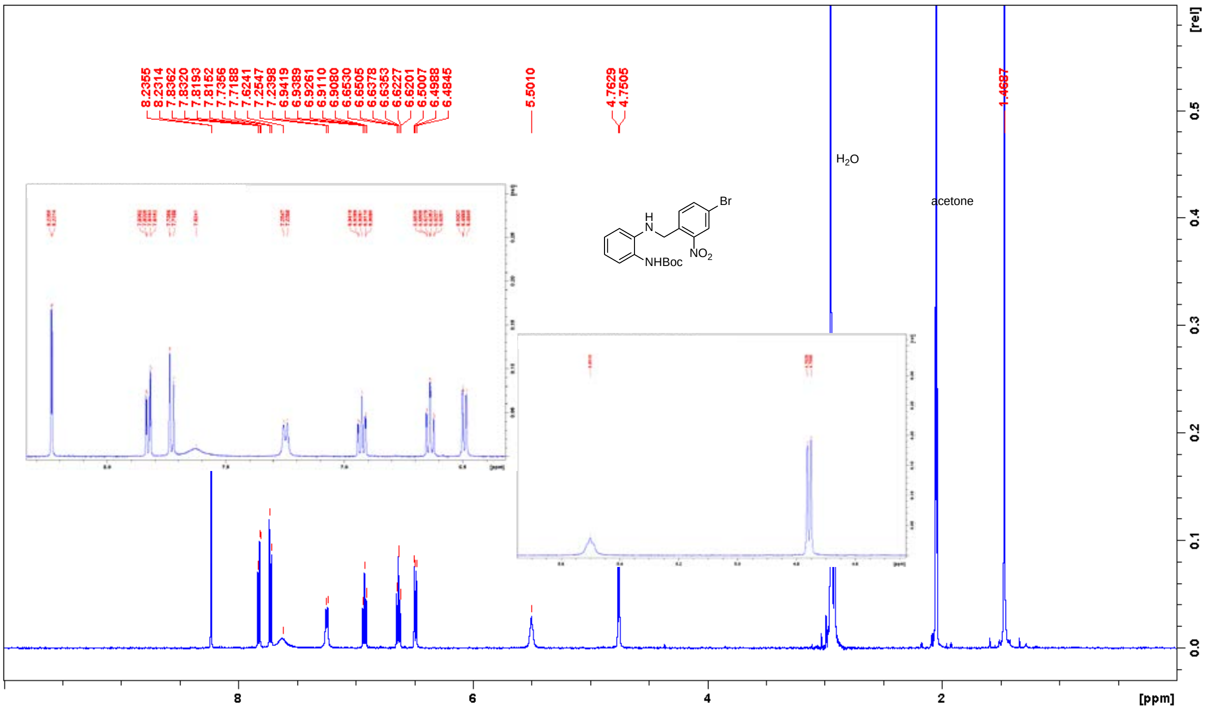

Figure S8: ^{1}H NMR spectrum of compound **4a**.

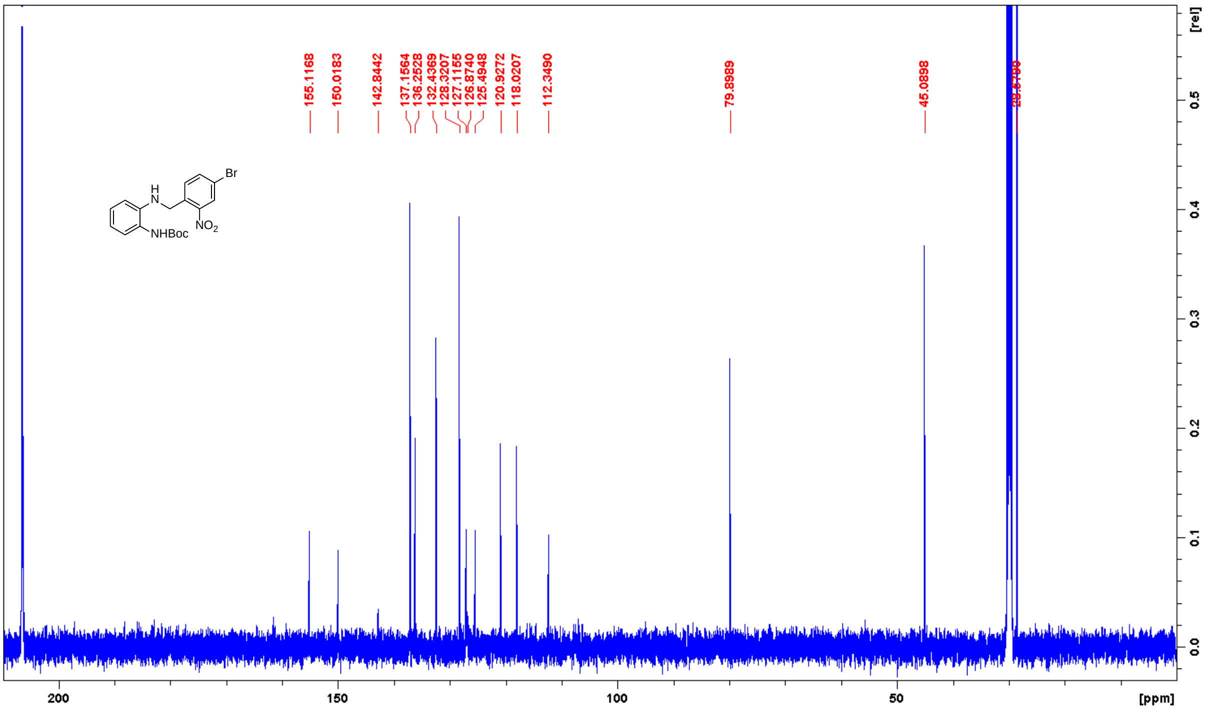

Figure S9: ^{13}C NMR spectrum of compound **4a**.

II.6 Synthesis of (9*H*-fluorene-9-yl)methyl (4-bromo-2-nitrobenzyl)(2-((*tert*-butoxycarbonyl)-amino)phenyl)carbamate (5a)

Under a nitrogen atmosphere, a solution of *tert*-butyl (2-((4-bromo-2-nitrobenzyl)amino)phenyl)-carbamate (**4a**, 1.70 g, 4.94 mmol) in anhydrous DMF (20 mL) was prepared, and then DIPEA (861 µL, 4.94 mmol) and 9-fluorenylmethoxycarbonyl chloride (2.40 g, 9.89 mmol) dissolved in anhydrous DMF (20 mL) were added. The reaction mixture was stirred at rt for 48 h and after that, the solvent was evaporated. Then, 50 mL of water and 50 mL of DCM were added to the residue. The organic layer was separated and it was extracted twice with DCM. The combined organic layers were dried over $MgSO_4$ and the solvent was evaporated. Flash column chromatography on silica (0.040–0.063 mm, ethyl acetate/cyclohexane 1:3, R_f = 0.44) gave a colorless solid (**5a**, 1.78 g, 2.76 mmol, 56%).

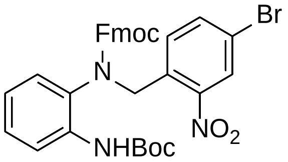

melting point: 164.3 °C

^{1}H NMR (500 MHz, acetone-d$_6$, 298 K): δ = 8.14 (d, ^4J = 2.1 Hz, 1 H), 8.03-7.66 (m, 5 H), 7.55-7.00 (m, 10 H), 5.27-4.88 (m, 2 H, aliph. *H*), 4.61-3.96 (m, 3 H, aliph. *H*), 1.46 (s, 9H, C*H*$_3$) ppm.

^{13}C NMR (125 MHz, acetone-d$_6$, 298 K): δ = 155.54 (*C*=O), 142.10, 137.03, 129.18, 128.49, 128.20, 127.86, 126.07, 124.43, 120.74, 80.46, 68.43, 47.83, 28.48 (*C*H$_3$) ppm.

IR (ATR): $\tilde{\nu}$ = 3380 (w), 2977 (w), 1719 (s), 1595 (w), 1535 (s), 1518 (s), 1477 (w), 1450 (m), 1390 (m), 1342 (m), 1292 (m), 1278 (m), 1218 (m), 1187 (m), 1153 (s), 1042 (w), 1025 (w), 976 (w), 942 (w), 933 (w), 885 (w), 843 (w), 801 (w), 756 (s), 739(s), 670 (w), 645 (w), 618 (m), 535 (m) cm^{-1}.

MS (ESI HR, $CHCl_3$/MeOH): *m/z* ($C_{33}H_{30}{}^{79}BrN_3O_6$+$H^+$) = calc.: 644.13907, found: 644.13932 ± 0.38 ppm.

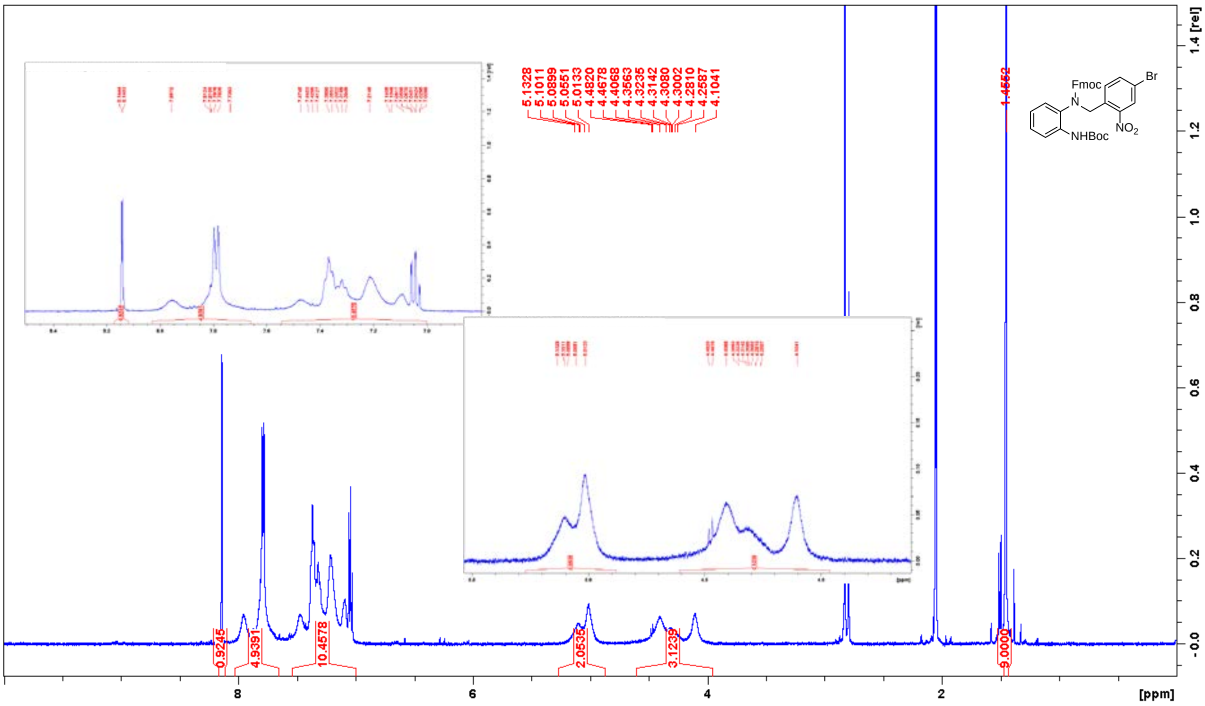

Figure S10: ^{1}H NMR spectrum of compound **5a**.

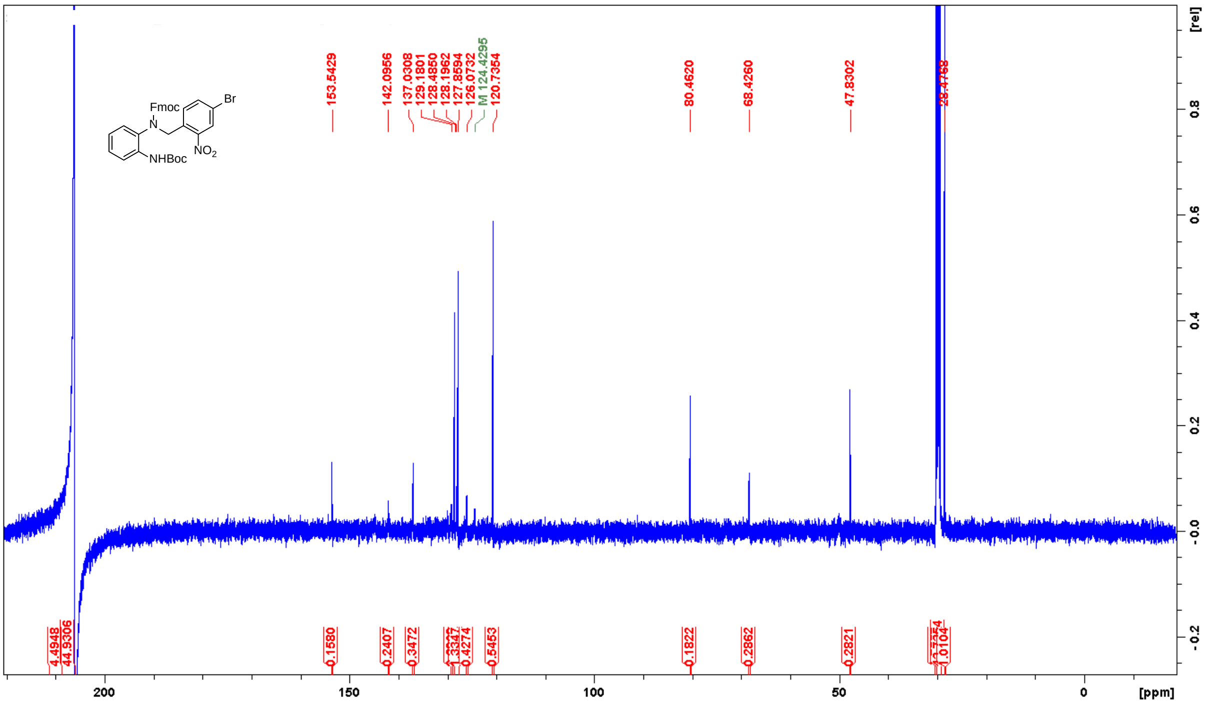

Figure S11: ^{13}C NMR spectrum of compound **5a**.

II.7 Synthesis of (9*H*-fluorene-9-yl)methyl (2-aminophenyl)(4-bromo-2-nitrobenzyl)carbamate (6a)

(9*H*-Fluorene-9-yl)methyl (4-bromo-2-nitrobenzyl)(2-((*tert*-butoxycarbonyl)amino)phenyl)carbamate (**5a**, 1.20 g, 1.86 mmol) was dissolved in 25 mL of DCM and 15 mL of TFA was added. The reaction mixture was stirred at rt for 16 h and then neutralized with saturated aqueous $NaHCO_3$. The organic layer was separated and it was extracted twice with DCM. The combined organic layers were dried over $MgSO_4$ and the solvent was evaporated. A pale yellow solid (**6a**, 1.01 g, 1.86 mmol, quant.) was obtained.

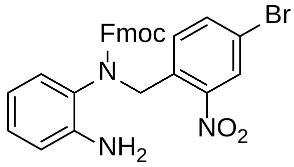

melting point: 79.6 °C

^{1}H NMR (500 MHz, acetone-d$_6$, 298 K): δ = 8.12 (d, ^4J = 1.8 Hz, 1 H), 7.98-7.58 (m, 4 H), 7.58-6.50 (m, 10 H), 5.58-3.89 (m, 5 H) ppm.

^{13}C NMR (125 MHz, acetone-d$_6$, 298 K): δ = 144.90, 142.13, 136.87, 133.24, 130.35, 129.02, 128.45, 128.05, 127.85, 127.81, 126.17, 124.44, 121.50 120.73, 120.68, 68.41, 49.68, 47.99, 47.90 ppm.

IR (ATR): $\tilde{\nu}$ = 3363 (w), 2251 (w), 1965 (s), 1621 (w), 1528 (s), 1503 (m), 1450 (w), 1404 (w), 1340 (m), 1299 (s), 1136 (m), 1101 (w), 1022 (w), 988 (w), 877 (m), 797 (w), 757 (s), 739 (s), 621 (w), 534 (w) cm^{-1}.

MS (ESI HR, $CHCl_3$/MeOH): *m/z* ($C_{28}H_{22}{}^{79}BrN_3O_4+H^+$) = calc.: 544.08665, found: 544.08639 ± 0.48 ppm.

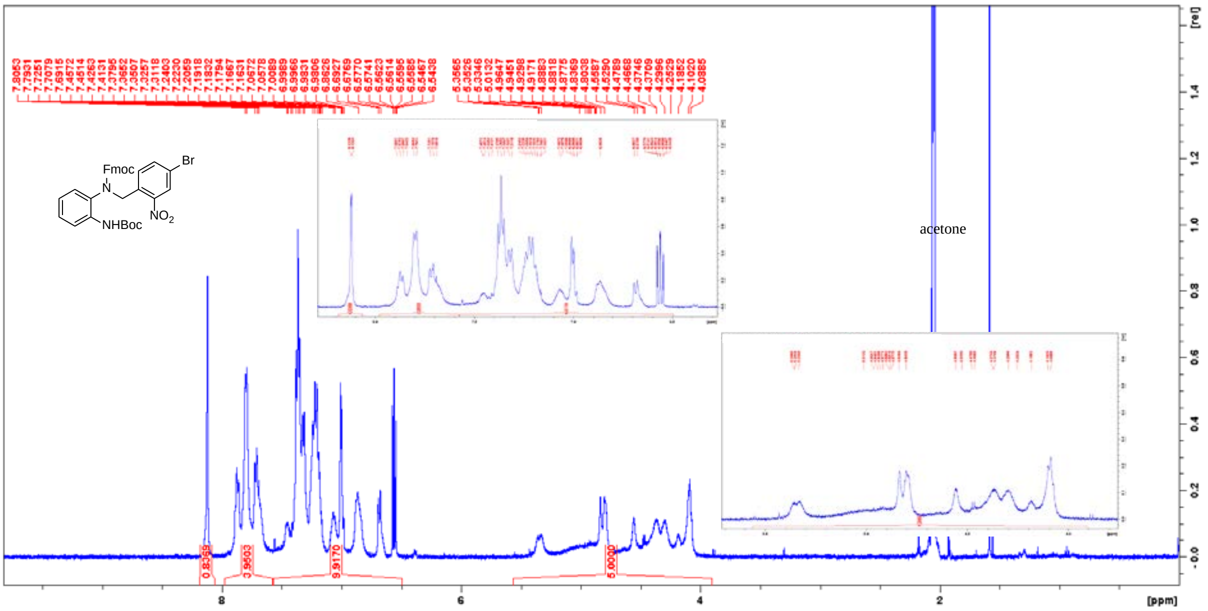

Figure S12: ^{1}H NMR spectrum of compound **6a**.

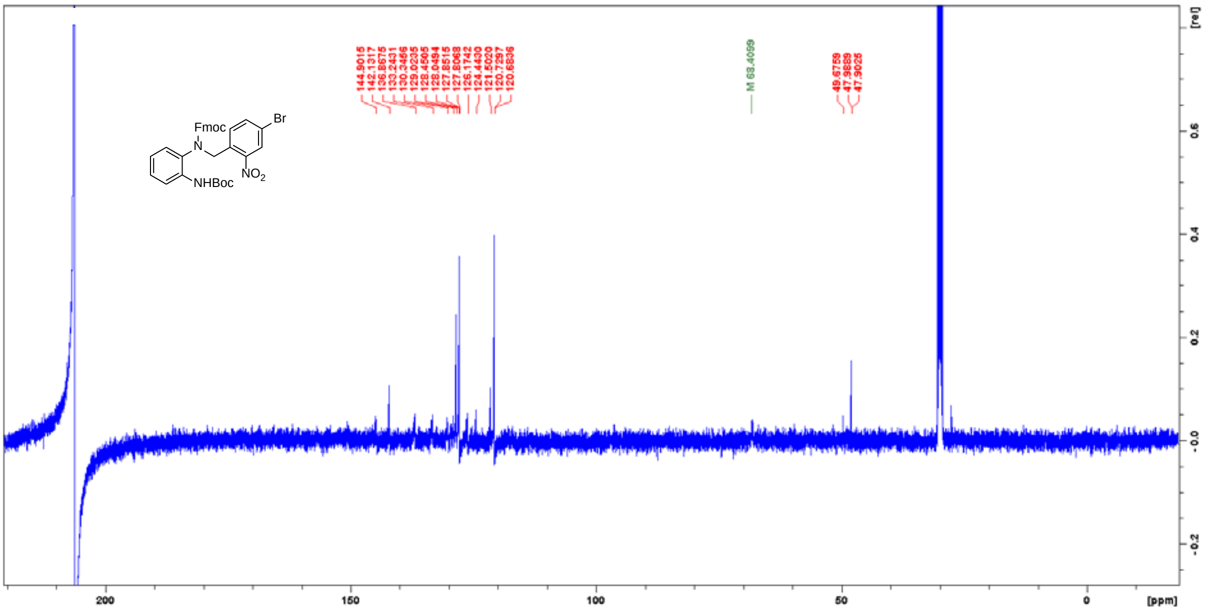

Figure S13: ^{13}C NMR spectrum of compound **6a**.

II.8 Synthesis of (9*H*-fluorene-9-yl)methyl (*Z*)-3-bromodibenzo[*c*,*g*][1,2,5]triazocine-11(12*H*)-carboxylate (8a)

(9*H*-Fluorene-9-yl)methyl (2-aminophenyl)(4-bromo-2-nitrobenzyl)carbamate (**6a**, 858 mg, 1.58 mmol) was suspended in 100 mL of ethanol and 50 mL of a 1 M NH_4Cl solution in water was added. The reaction mixture was heated to 75 °C and zink powder (824 mg, 12.6 mmol, 8 equiv) was added. The reaction mixture was stirred at 75 °C for 1 h and then hot filtrated. The solvent was evaporated and DCM (50 mL) and deionized water (50 mL) were added to the residue. The organic layer was separated and it was extracted twice with DCM. The combined organic layers were dried over $MgSO_4$ and the solvent was evaporated. The crude product was dissolved in 50 mL of acetic acid and *m*CPBA (708 mg, 3.15 mmol, ≥ 77%) dissolved in 50 mL of acetic acid was added dropwise over a period of 2 h. The reaction mixture was stirred at rt overnight and after that, the solvent was evaporated. Then, 50 mL of DCM (50 mL) and half-concentrated $NaHCO_3$ (50 mL, dissolved in deionized water) were added to the residue and the organic layer was separated. It was extracted twice with DCM and the combined organic layers were dried over $MgSO_4$. The solvent was evaporated and flash column chromatography on silica (0.040–0.063 mm, ethyl acetate/cyclohexane 1:3, R_f = 0.51) gave a yellow solid (**8a**, 451 mg, 883 µmol, 56%).

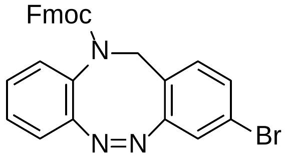

melting point: 91.4 °C

^{1}H NMR (500 MHz, acetone-d$_6$, 298 K): δ = 8.18-6.66 (m, 15 H, Ar-*H*), 5.13-3.87 (m, 5 H, aliph. *H*) ppm.

^{13}C NMR (125 MHz, acetone-d$_6$, 298 K): δ = 154.75, 142.11, 132.72, 131.30, 130.53, 129.56, 129.13, 128.56, 127.92, 126.14, 124.23, 122.63, 120.85, 119.66, 68.52, 52.74, 47.99, 47.78 ppm.

IR (ATR): $\tilde{\nu}$ = 2924 (w), 1702 (s), 1591 (w), 1476 (w), 1449 (m), 1390 (s), 1321 (s), 1295 (s), 1242 (w), 1128 (w), 1078 (w), 1041 (m), 102 (w), 866 (w), 818 (w), 757 (s), 737 (s), 694 (w), 670 (w), 650 (w), 621 (w), 604 (w), 549 (m), 513 (w) cm^{-1}.

MS (ESI HR, $CHCl_3$/MeOH): *m/z* ($C_{28}H_{20}{}^{79}BrN_3O_2+H^+$) = calc.: 510.08117, found: 510.08153 ± 0.7 ppm.

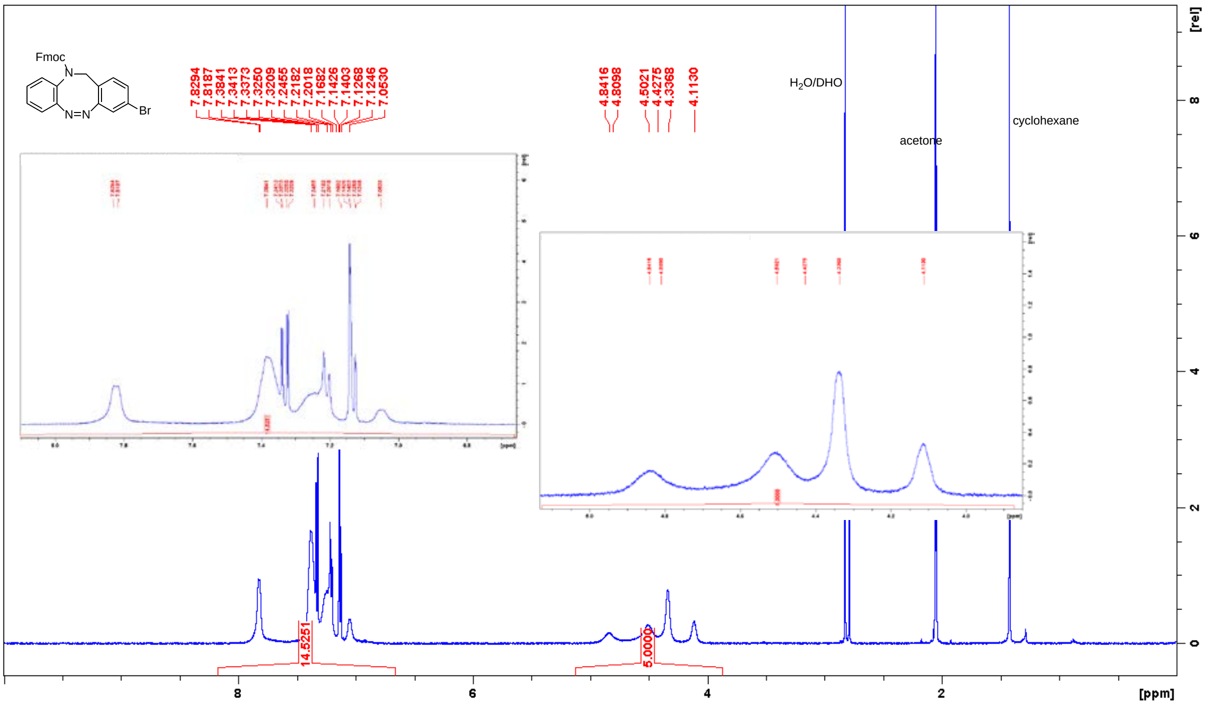

Figure S14: ^{1}H NMR spectrum of compound **8a**.

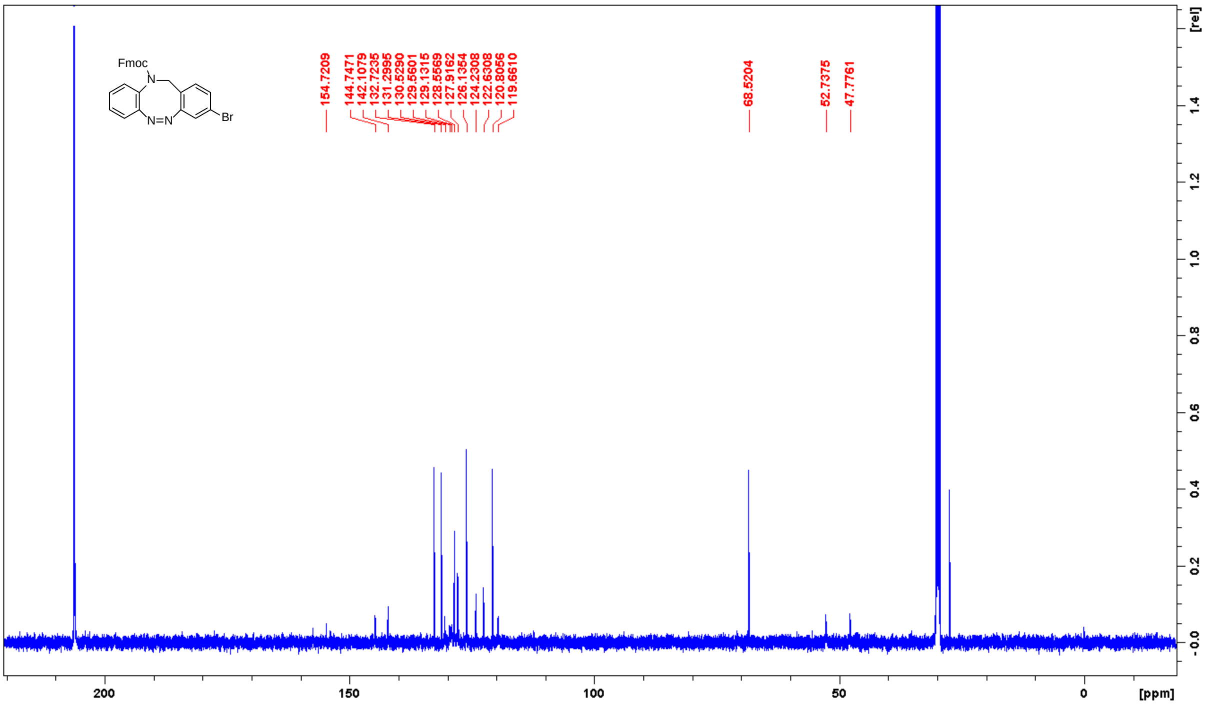

Figure S15: ^{13}C NMR spectrum of compound **8a**.

II.9 Synthesis of (*Z*)-3-bromo-11,12-dihydrodibenzo[*c*,*g*][1,2,5]triazocine (9a)

(9*H*-Fluorene-9-yl)methyl (*Z*)-3-bromodibenzo[*c*,*g*][1,2,5]triazocine-11(12*H*)-carboxylate (**8a**, 382 mg, 748 µmol) was dissolved in 10 mL of DCM and 25 mL of NEt_3 was added. The reaction mixture was stirred at rt for 22 h. The solvent was removed and flash column chromatography on silica (0.040–0.063 mm, ethyl acetate/cyclohexane 1:2, R_f = 0.54) gave a red solid (**9a**, 201 mg, 698 µmol, 93%).

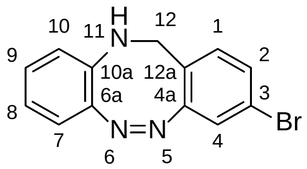

melting point: 160.3 °C

^{1}H NMR (500 MHz, acetone-d$_6$, 298 K): δ = 7.44 (dd, ^3J = 8.1 Hz, ^4J = 2.0 Hz, 1 H, *H*-2), 7.35 (d, ^4J = 2.0 Hz, 1 H, *H*-4), 7.31 (d, ^3J = 8.1 Hz, 1 H, *H*-1), 6.88 (td, ^3J = 7.7 Hz, ^4J = 1.6 Hz, 1 H, *H*-9), 6.75 (dd, ^3J = 8.0 Hz, ^4J = 1.6 Hz, 1 H, *H*-7), 6.65 (td, ^3J = 7.5 Hz, ^4J = 1.2 Hz, 1 H, *H*-8), 6.56 (dd, ^3J = 8.2 Hz, ^4J = 1.0 Hz, 1 H, *H*-10), 5.42 (m$_c$, 1 H, N*H*), 3.98 (m$_c$, 2 H, CH_2) ppm.

^{13}C NMR (125 MHz, acetone-d$_6$, 298 K): δ = 160.24 (*C*-4a), 144.64 (*C*-6a), 137.04 (*C*-10a), 132.43 (*C*-1), 131.85 (*C*-2), 129.06 (*C*-9), 124.00 (*C*-3), 123.56 (*C*-7), 122.67 (*C*-4), 121.76 (*C*-12a), 119.93 (*C*-10), 118.31 (*C*-8), 47.15 (*C*H_2) ppm.

IR (ATR): $\tilde{\nu}$ = 3322 (m), 2959 (w), 1601 (m), 1587 (m), 1568 (w), 1513 (m), 1480 (s), 1456 (m), 1422 (w), 1389 (m), 1363 (w), 1251 (m), 1191 (w), 1161 (m), 1119 (m), 1093 (m), 1072 (m), 956 (w), 898 (m), 831 (s), 812 (s); 768 (m), 741 (s), 720 (s), 703 (w), 651 (w), 604 (m), 560 (w), 513 (m) cm^{-1}.

MS (ESI HR, $CHCl_3$/MeOH): *m/z* ($C_{13}H_{10}{}^{79}BrN_3+H^+$) = calc.: 288.01309, found: 288.01278 ± 1.07 ppm.

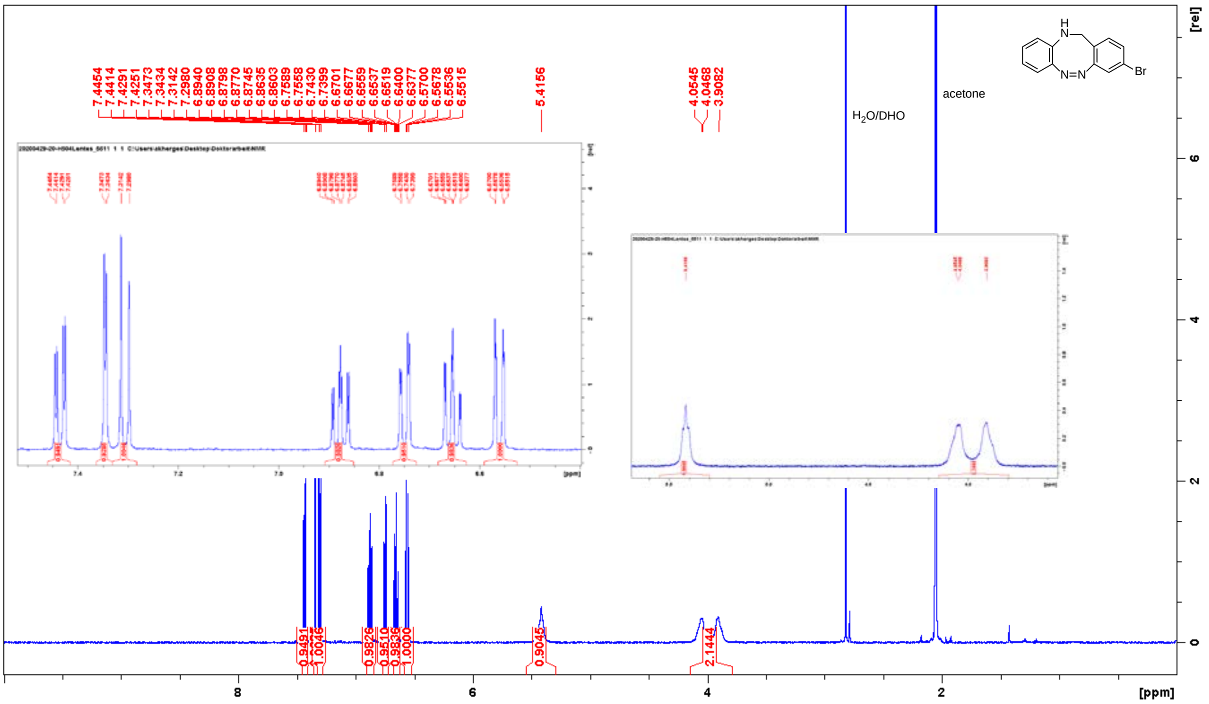

Figure S16: ^{1}H NMR spectrum of compound **9a**.

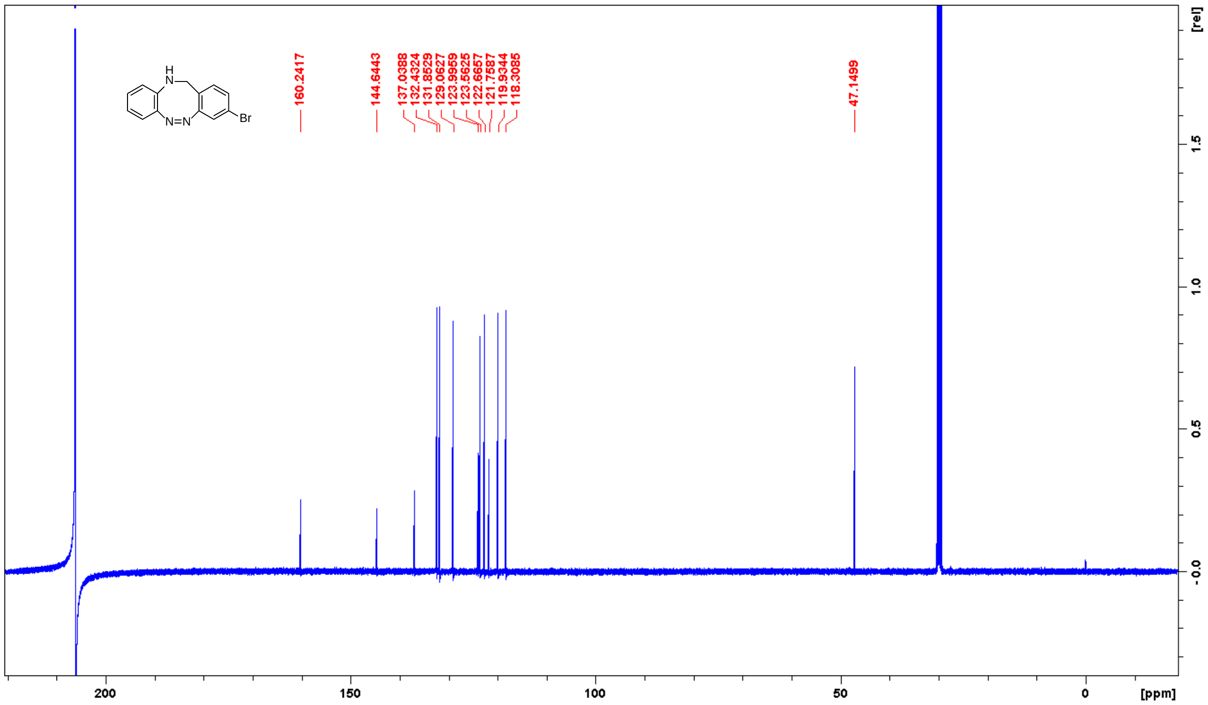

Figure S17: ^{13}C NMR spectrum of compound **9a**.

II.10 Synthesis of (*Z*)-1-(3-bromodibenzo[*c*,*g*][1,2,5]triazocin-11(12*H*)-yl)ethan-1-one (10a)

Under a nitrogen atmosphere, a solution of (*Z*)-3-bromo-11,12-dihydrodibenzo[*c*,*g*][1,2,5]triazocine (**9a**, 52 mg, 180 µmol) in anhydrous DMF (5 mL) was prepared, and then TEA (503 µL, 20 equiv) and HOAc (103 µL, 1.80 mmol) were added. The reaction mixture was cooled to 0 °C and T3P (1.08 mL, 1.80 mmol, 50% in ethyl acetate) was added dropwise. The ice bath was removed and it was stirred for 22 h. Then, 50 mL DCM and 50 mL deionized water were added and the organic layer was separated. It was extracted twice with DCM and the combined organic layers were dried over $MgSO_4$. The solvent was removed and flash column chromatography on silica (0.040–0.063 mm, ethyl acetate/cyclohexane 1:1, R_f = 0.31) gave a pale yellow solid (**10a**, 51 mg, 154 µmol, 86%).

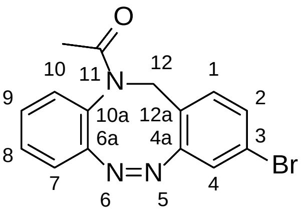

melting point: 164.9 °C

^{1}H NMR (500 MHz, acetone-d$_6$, 298 K): δ = 7.41 (td, ^3J = 7.5 Hz, ^4J = 1.8 Hz, 1 H, *H*-8/9), 7.34-7.36 (m, 3 H, *H*-2, Ar-*H*, Ar-*H*), 7.19 (d, ^3J = 7.2 Hz, 1 H, *H*-1), 7.12 (d, ^4J = 2.0 Hz, 1 H, *H*-4), 7.06 (dd, ^3J = 7.8 Hz, ^4J = 1.4 Hz, 1 H, *H*-7/10), 5.02 (d, ^2J = 14.5 Hz, 1 H, C*H*$_2$a), 4.32 (d, ^2J = 14.5 Hz, 1 H, C*H*$_2$b), 1.80 (s, 3 H, C*H*$_3$) ppm.

^{13}C NMR (125 MHz, acetone-d$_6$, 298 K): δ = 169.39 (*C*=O), 157.21 (*C*-4a), 154.16 (*C*-6a/10a), 132.77 (*C*-1), 131.29 (Ar-*C*), 130.29 (Ar-*C*), 130.26 (*C*-8/9), 129.57 (Ar-*C*), 129.45 (*C*-6a/10a), 124.74 (*C*-12a), 122.69 (*C*-4), 122.34 (*C*-3), 120.02 (*C*-7/10), 51.53 (*C*H$_2$), 20.04 (*C*H$_3$) ppm.

IR (ATR): $\tilde{\nu}$ = 3048 (w), 2924 (w), 1658 (s), 1590 (m), 1516 (w), 1475 (m), 1380 (s), 1338 (s), 1294 (m), 1242 (w), 1161 (w), 1115 (w), 1085 (w), 1070 (w), 1006 (w), 963 (w), 943 (w), 925 (w), 888 (w), 872 (w), 848 (m), 815 (s), 789 (w), 763 (s), 739 (m), 689 (w), 659 (m), 626 (w), 596 (m), 576 (m), 584 (s), 518 (m) cm^{-1}.

MS (ESI HR, CHCl$_3$/MeOH): *m/z* ($C_{15}H_{12}{}^{79}BrN_3+H^+$) = calc.: 330.02365, found: 330.02334 ± 0.95 ppm.

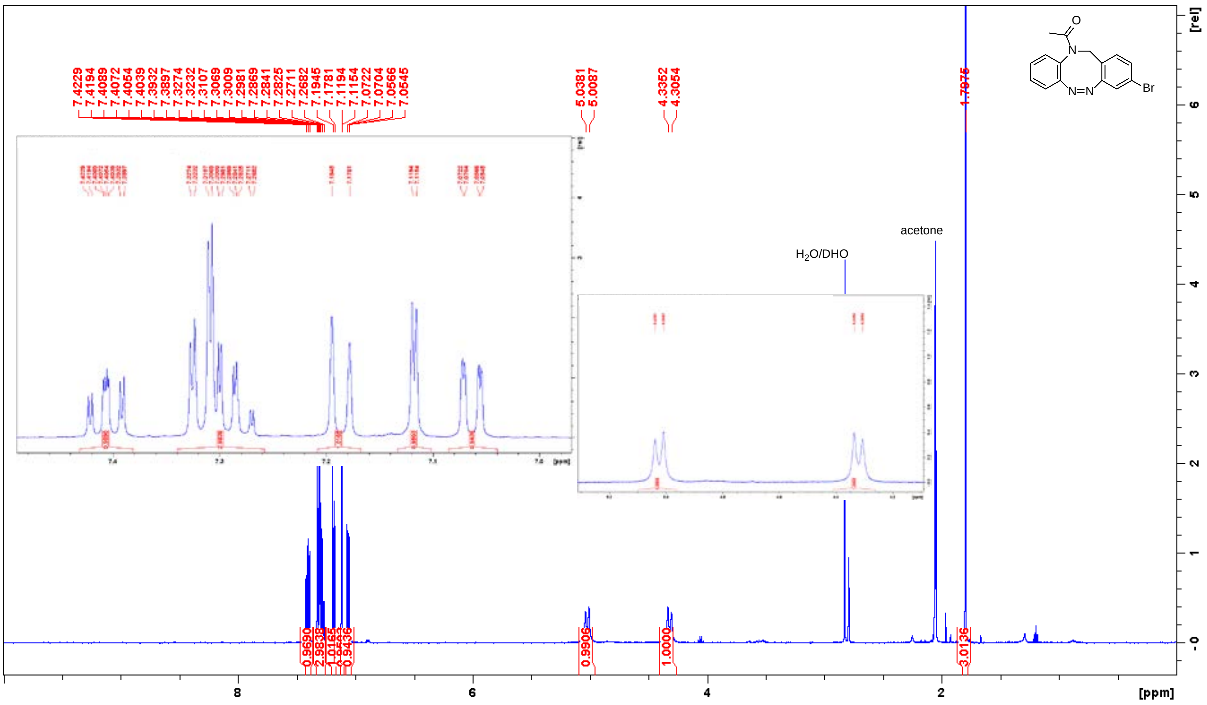

Figure S18: ^{1}H NMR spectrum of compound **10a**.

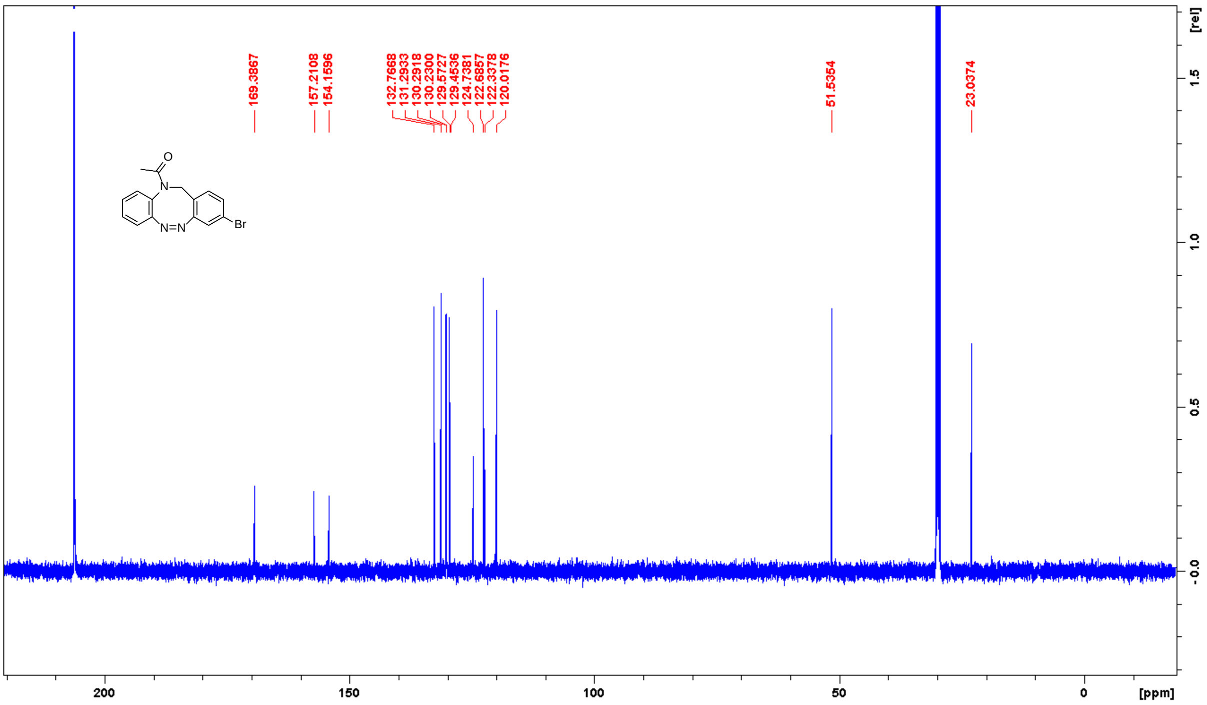

Figure S19: ^{13}C NMR spectrum of compound **10a**.

II.11 Synthesis of (*Z*)-3-bromodibenzo[*c*,*g*][1,2,5]triazocine-11(12*H*)-carbaldehyde (11a)

Under a nitrogen atmosphere, a solution of (*Z*)-3-bromo-11,12-dihydrodibenzo[*c*,*g*][1,2,5]triazocine (**9a**, 100 mg, 348 µmol) in anhydrous MeCN (6 mL) was prepared, and then DIPEA (604 µL, 3.48 mmol) and chloral (338 µL, 3.48 mmol) were added. The reaction mixture was stirred at rt for 20 h. The solvent was removed and flash column chromatography on silica (0.040–0.063 mm, ethyl acetate/cyclohexane 1:2, R_f = 0.35) gave a pale yellow solid (**11a**, 92 mg, 291 µmol, 84%).

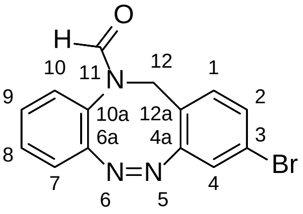

melting point: 201.8 °C

^{1}H NMR (500 MHz, acetone-d$_6$, 298 K): δ = 8.36 (s, 1 H, C*H*O), 7.39 (dd, ^3J = 8.2 Hz, ^4J = 2.0 Hz, 1 H, *H*-2), 7.36 (td, ^3J = 7.7 Hz, ^4J = 1.3 Hz, 1 H, *H*-8/9), 7.31 (d, ^3J = 8.1 Hz, 1 H, *H*-1), 7.28-7.23 (m, H, *H*-2, *H*-4, *H*-8/9), 7.18 (dd, ^3J = 7.9 Hz, ^4J = 1.2 Hz, 1 H, *H*-7/10), 6.97 (dd, ^3J = 7.8 Hz, ^4J = 1.4 Hz, 1 H, *H*-7/10), 4.52 (m$_c$, 2 H, C*H*$_2$) ppm.

^{13}C NMR (125 MHz, acetone-d$_6$, 298 K): δ = 162.30 (*C*HO), 158.37 (*C*-4a), 152.77 (*C*-6a/10a), 133.31 (*C*-1), 131.69 (*C*-2), 129.45 (*C*-8/9), 129.25 (*C*-8/9), 128.29 (*C*-7/10), 123.19 (*C*-3), 122.88 (*C*-12a), 122.21 (*C*-4), 120.51 (*C*-7/10), 48.98 (*C*H$_2$) ppm.

IR (ATR): $\tilde{\nu}$ = 3353 (w), 3038 (w), 1682 (s), 1621 (m), 1501 (s), 1450 (m), 1409 (s), 1356 (m), 1310 (s), 1276 (s), 1215 (m), 1128 (m), 1096 (w), 1007 (w), 979 (m), 939 (w), 860 (w), 739 (s), 631 (w), 621 (w), 552 (w), 525 (m) cm^{-1}.

MS (ESI HR, CHCl$_3$/MeOH): *m/z* ($C_{14}H_{10}{}^{79}BrN_3O+H^+$) = calc.: 316.00800, found: 316.00781 ± 0.59 ppm.

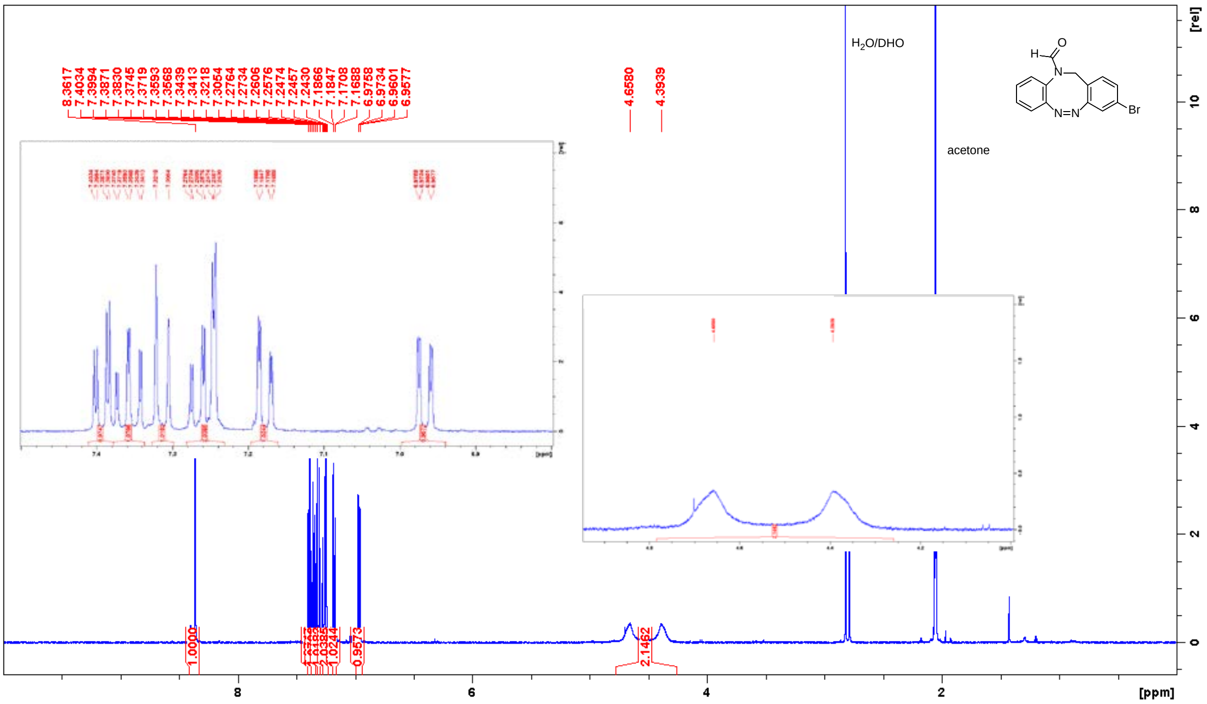

Figure S20: ^{1}H NMR spectrum of compound **11a**.

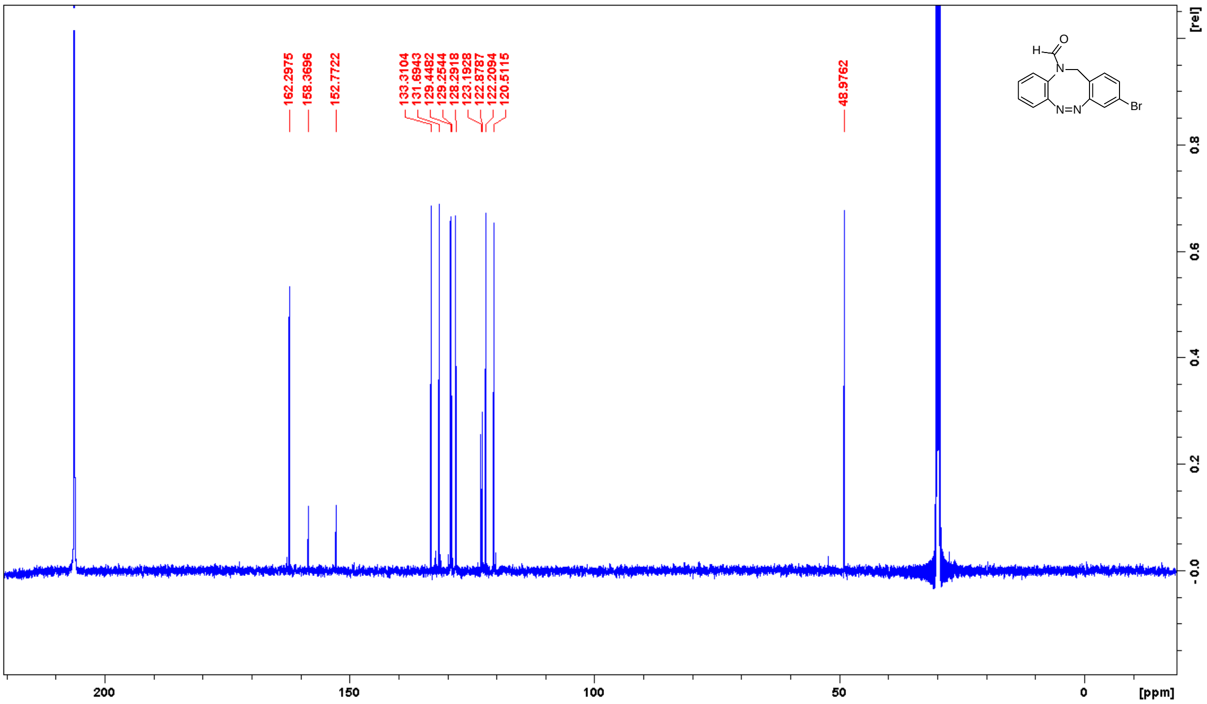

Figure S21: ^{13}C NMR spectrum of compound **11a**.

II.12 Synthesis of 1-(bromomethyl)-4-iodo-2-nitrobenzene (3b)

Under a nitrogen atmosphere, a solution of 4-iodo-1-methyl-2-nitrobenzene (5.00 g, 19.0 mmol) in anhydrous MeCN (21 mL) was prepared, and then NBS (4.00 g, 22.6 mmol) and AIBN (97.2 mg, 600 µmol) were added. The reaction mixture was refluxed for 24 h. The solvent was removed and flash column chromatography on silica (0.040–0.063 mm, ethyl acetate/cyclohexane 1:12, R_f = 0.43) gave a brown solid (**3b**, 1.07 g, 3.12 mmol, 16%).

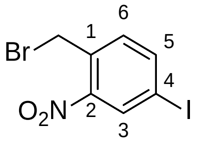

melting point: 103.9 °C

^{1}H NMR (500 MHz, $CDCl_3$, 298 K): δ = 8.35 (d, ^4J = 1.8 Hz, 1 H, *H*-3), 7.93 (dd, ^3J = 8.2 Hz, ^4J = 1.8 Hz, 1 H, *H*-5), 7.30 (d, ^3J = 8.2 Hz, *H*-6), 7.46 (s, 2 H, CH_2) ppm.

^{13}C NMR (125 MHz, $CDCl_3$, 298 K): δ = 148.06 (*C*-2), 142.77 (*C*-5), 134.29 (*C*-3), 133.91 (*C*-6), 132.58 (*C*-1), 93.60 (*C*-4), 28.18 (CH_2) ppm.

IR (ATR): $\tilde{\nu}$ = 3081 (w), 1702 (w), 1592 (w), 1555 (w), 1518 (s), 1476 (w), 1433 (m), 1342 (m), 1275 (w), 1223 (m), 1204 (w), 1164 (w), 1124 (w), 1077 (w), 971 (w), 897 (w), 889 (w), 870 (m), 837 (m), 804 (m), 762 (w), 705 (w), 681 (w), 627 (w), 611 (m), 567 (w) cm^{-1}.

MS (EI, 70 eV): *m/z*(%) = 341 (11) $[M]^+$, 262 (100) $[M-Br]^+$, 242 (5) $[M-BrO]^+$.

MS (EI, HR, 70 eV): *m/z* ($C_7H_5{}^{79}BrINO_2+H^+$) = calc.: 330.85483, found: 340.85328 ± 4.65 ppm.

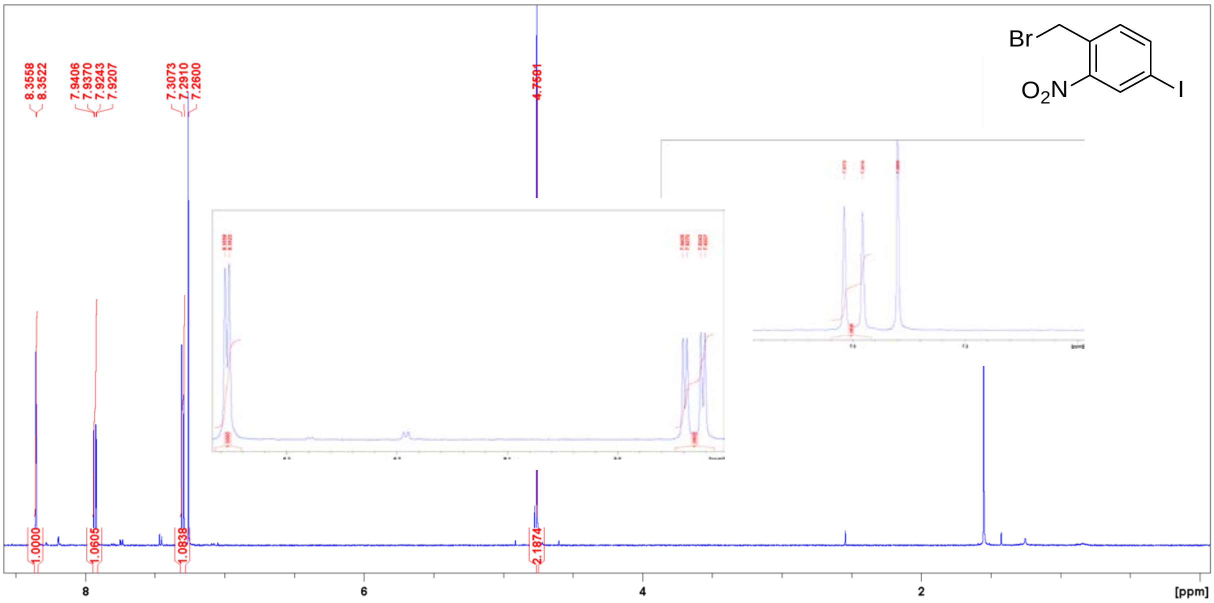

Figure S22: ^{1}H NMR spectrum of compound **3b**.

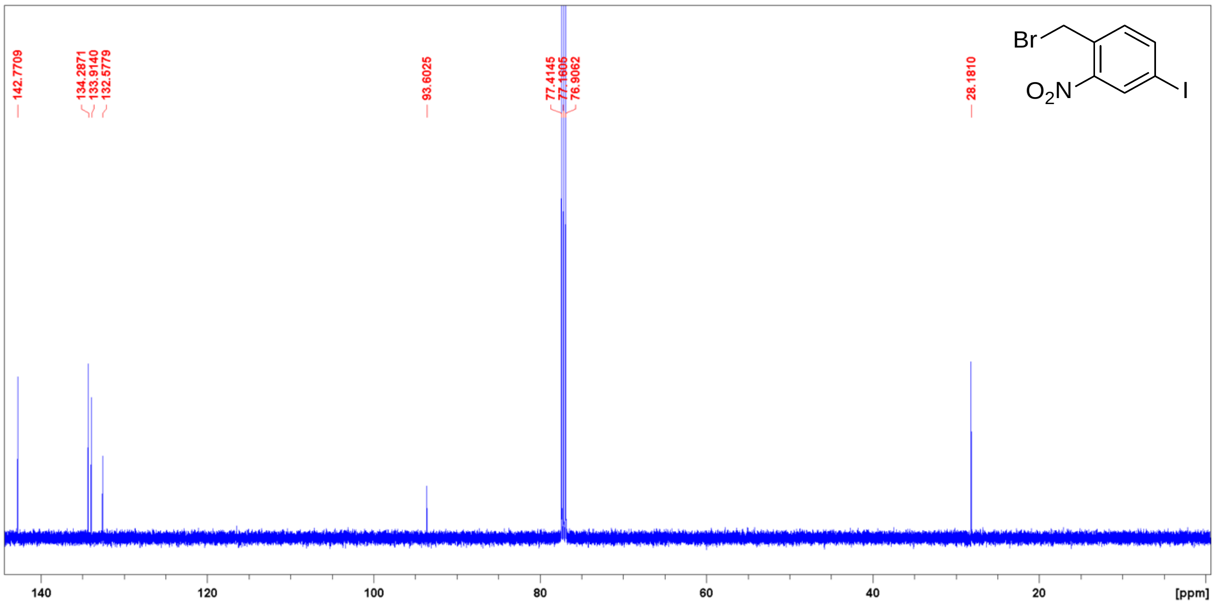

Figure S23: ^{13}C NMR spectrum of compound **3b**.

II.13 Synthesis of *tert*-butyl (2-((4-iodo-2-nitrobenzyl)amino)phenyl)carbamate (4b)

Under a nitrogen atmosphere, a solution of *tert*-butyl (2-aminophenyl)carbamate[1] (**2**, 651 mg, 3.12 mmol) in anhydrous THF (12 mL) was prepared, and then TEA (477 µL, 3.42 mmol) and 1-(bromomethyl)-4-iodo-2-nitrobenzene (**3b**, 1.07 g, 3.12 µmol) were added. The reaction mixture was refluxed for 20 h and after that, the solvent was removed. Deionized water and DCM were added, the organic layer was separated and it was extracted twice with DCM. The combined organic layers were dried over $MgSO_4$ and the solvent was evaporated. Recrystallization from ethyl acetate/cyclohexane 1:1 gave a yellow solid (**4b**, 1.41 g, 3.00 mmol, 96%).

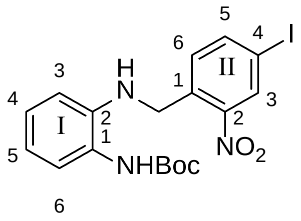

melting point: 143.5 °C

^{1}H NMR (500 MHz, $CDCl_3$, 298 K): δ = 8.38 (d, ^4J = 1.8 Hz, 1 H, Ar^{II}-*H*-3), 7.85 (dd, ^3J = 8.2 Hz, ^4J = 1.8 Hz, 1 H, Ar^{II}-*H*-5), 7.40 (d, ^3J = 8.2 Hz, 1H, Ar^{II}-*H*-6), 7.25 (d, ^3J = 7.7 Hz, 1 H, Ar^{I}-*H*-6), 7.01 (td, ^3J = 7.7 Hz, ^4J = 1.5 Hz, 1 H, Ar^{I}-*H*-4), 6.80 (t, ^3J = 7.7 Hz, 1 H, Ar^{I}-*H*-5), 6.52 (d, ^3J = 7.7 Hz, 1 H, Ar^{I}-*H*-3), 6.28 (br. s, 1 H, N*H*), 4.69 (s, 2 H, -C*H*$_2$), 1.51 (s, 9 H, tBu-*H*) ppm.

^{13}C NMR (125 MHz, $CDCl_3$, 298 K): δ = 154.39 (*C*=O),148.69 (Ar^{II}-*C*-2), 140.69 (Ar^{I}-*C*-2), 142.74 (Ar^{II}-*C*-5), 134.55 (Ar^{II}-*C*-1), 133.81 (Ar^{II}-*C*-3), 131.85 (Ar^{II}-*C*-6), 127.09 (Ar^{I}-*C*-4), 126.03 (Ar^{I}-*C*-6), 124.84 (Ar^{I}-*C*-1), 119.72 (Ar^{I}-*C*-5), 113.62 (Ar^{I}-*C*-3), 91.87 (Ar^{II}-*C*-4), 81.08 (-*C*-$(CH_3)_3$, 45.84 (-*C*H_2), 28.46 (-*C*H_3) ppm.

IR (ATR): $\tilde{\nu}$ = 3414 (w), 3314 (w), 2924 (w), 2847 (w), 1679 (m), 1606 (m), 1531 (m), 1503 (m), 1460 (m), 1392 (w), 1363 (w), 1343 (m), 1329 (m), 1305 (w), 1285 (w), 1271 (m), 1247 (m), 1159 (s), 1133 (w), 1085 (w), 1055 (w), 1029 (w), 990 (w), 937 (w), 906 (w), 890 (w), 869 (w), 837 (w), 821 (w), 783 (w), 774 (w), 754 (w), 741 (w), 717 (w), 692 (w), 647 (w), 599 (w), 523 (w) cm^{-1}.

MS (ESI HR, $CHCl_3$/MeOH): *m/z* ($C_{18}H_{20}IN_3O_4+H^+$) = calc.: 470.05713, found: 470.05680 ± 0.69 ppm.

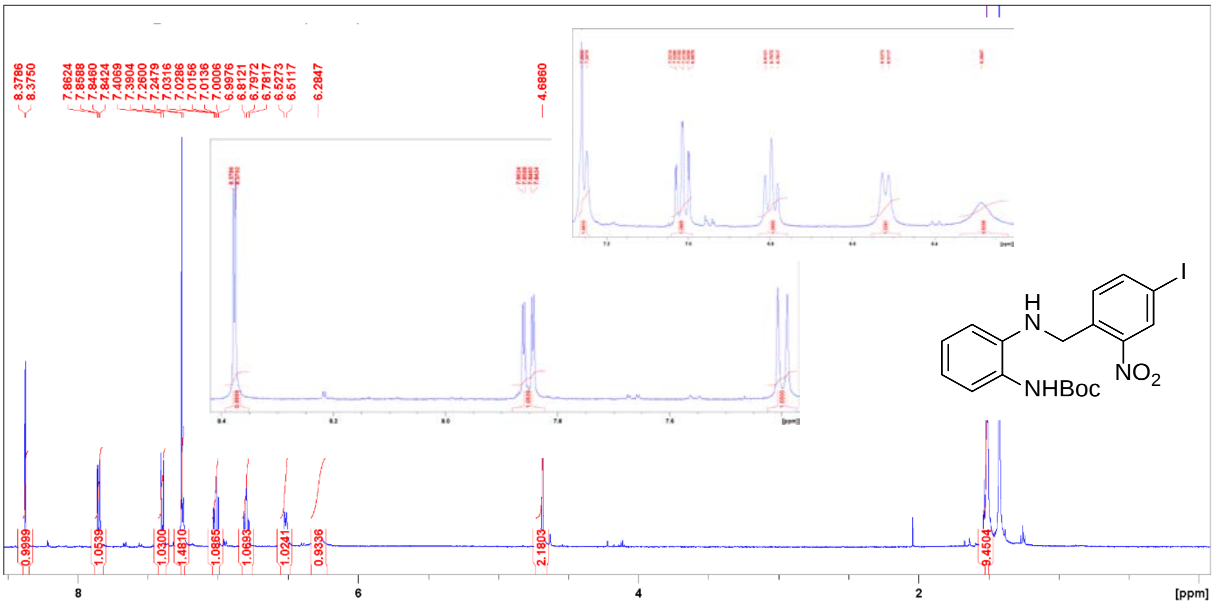

Figure S24: ^{1}H NMR spectrum of compound **4b**.

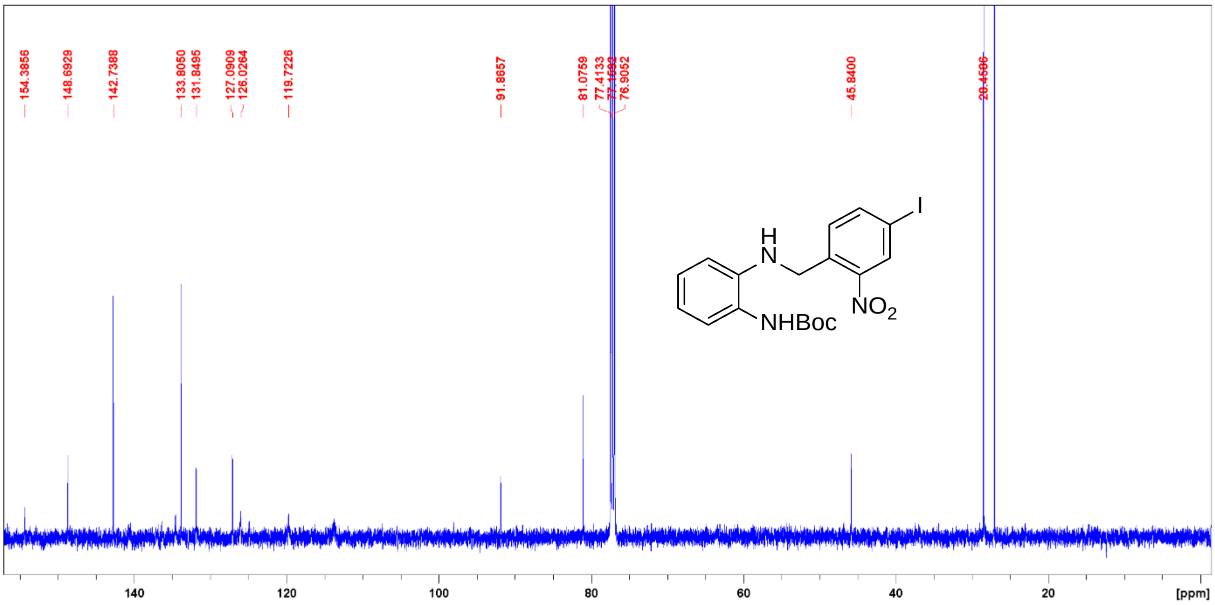

Figure S25: ^{13}C NMR spectrum of compound **4b**.

II.14 Synthesis of (9*H*-fluorene-9-yl)methyl (2-((*tert*-butoxycarbonyl)amino)phenyl)(4-iodo-2-nitrobenzyl)carbamate (5b)

Under a nitrogen atmosphere, a solution of *tert*-butyl (2-((4-iodo-2-nitrobenzyl)amino)phenyl)carbamate (**4b**, 1.41 g, 3.00 mmol) in anhydrous DMF (12 mL) was prepared, and then DIPEA (510 µL, 3.00 mmol) and Fmoc-chloride (1.55 g, 6.00 µmol) were added. The reaction mixture was stirred for 91 h at rt and after that, the solvent was evaporated. Deionized water and DCM were added, the organic layer was separated and it was extracted twice with DCM. The combined organic layers were dried over $MgSO_4$ and the solvent was evaporated. Flash column chromatography on silica (0.040–0.063 mm, ethyl acetate/cyclohexane 1:4, R_f = 0.45) gave a white solid (**5b**, 682 mg, 986 µmol, 33%).

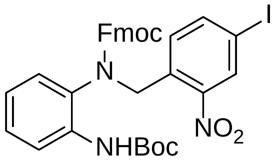

melting point: 131.6 °C

^{1}H NMR (500 MHz, acetone-d$_6$, 298 K): δ = 8.29 (d, ^{4}J = 1.7 Hz, 1 H, Ar-*H*), 8.14-7.55 (m, 5 H), 7.84-7.54-6.87 (m, 10 H), 5.12 (br. S, 1 H, -C*H*$_2$), 5.28-4.76 (m, 2 H), 4.73-3.96 (m, 3 H), 1.46 (s, 9 H, tBu-*H*) ppm.

^{13}C NMR (125 MHz, acetone-d$_6$, 298 K): δ = 153.53, 143.00, 142.08, 133.86, 129.96, 129.22, 128.48, 127.86, 126.06, 124.43, 122.55, 120.73, 92.68, 80.46, 68.42, 47.83, 28.60 ppm.

IR (ATR): $\tilde{\nu}$ = 2976 (w), 1706 (m), 1594 (w), 1524 (m), 1477 (s), 1449 1449, 1392 (w), 1366 (m), 1345 (m), 1295 (m), 1274 (m), 1239 (m), 1153 (s), 1046 (w), 1024 (m), 984 (w), 870 (w), 835 (w), 758 (m), 739 (s), 620 (w), 588 (w), 553 (w) cm^{-1}.

MS (ESI HR, $CHCl_3$/MeOH): *m/z* ($C_{33}H_{30}IN_3O_6$+H^+) = calc.: 692.12521, found: 692.12442 ± 1.14 ppm.

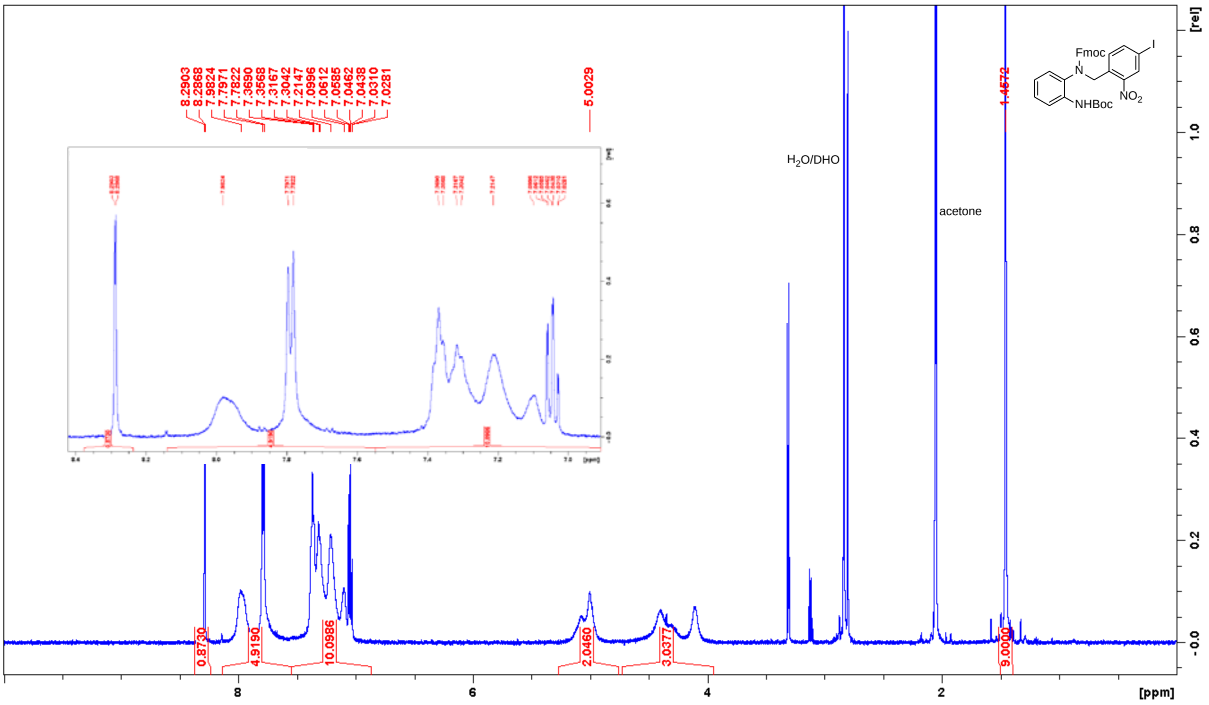

Figure S26: ^{1}H NMR spectrum of compound **5b**.

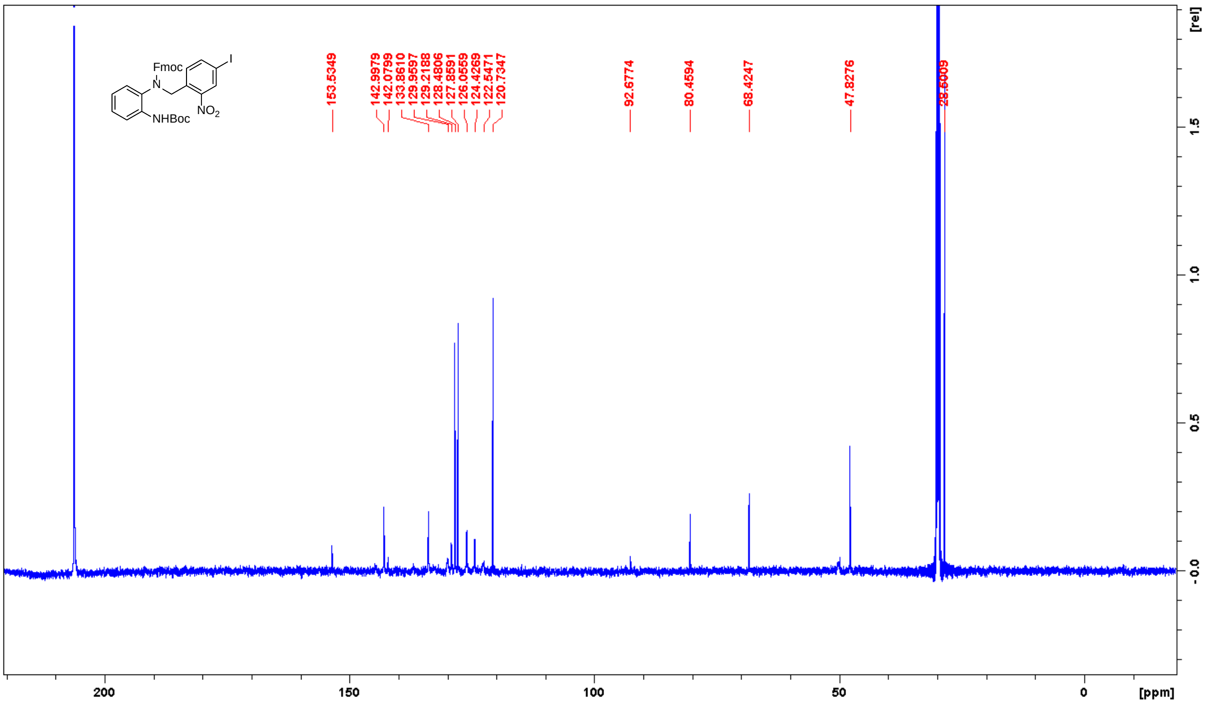

Figure S27: ^{13}C NMR spectrum of compound **5b**.

II.15 Synthesis of (9*H*-fluorene-9-yl)methyl (2-aminophenyl)(4-iodo-2-nitrobenzyl)carbamate (6b)

A solution of (9*H*-fluorene-9-yl)methyl (2-((*tert*-butoxycarbonyl)amino)phenyl)(4-iodo-2-nitrobenzyl)carbamate (**5b**, 2.15 g, 3.11 mmol) in DCM (20 mL) was prepared, and then TFA (12 mL) was added. The reaction mixture was stirred for 20 h at rt and after that, it was neutralized with saturated aqueous $NaHCO_3$ solution. The organic layer was separated and it was extracted twice with DCM. The combined organic layers were dried over $MgSO_4$ and the solvent was evaporated. A yellow solid (**6b**, 1.76 g, 2.97 mmol, 96%) was obtained.

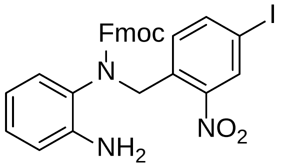

melting point: 85.7 °C

^{1}H NMR (500 MHz, $CDCl_3$, 298 K): δ = 8.24 (d, ^4J = 1.8 Hz, 1 H,), 7.87 (br. s, 1 H, 1 H), 7.71-7.62 (m, 2 H), 7.39-7.29 (m, 3 H), 7.24-7.07 (m, 5 H), 6.82-6.72 (m, 2 H), 6.70-6.65 (m, 1 H), 5.23 (br. s, 1 H, CH_2), 4.93 (br. s, 1 H, CH_2), 4.50-4.32 (m, 2 H, Fmoc-CH_2), 4.07 (br. s, 1 H, Fmoc-C*H*), 3.60 (br. s, 2 H, NH_2).

^{13}C NMR (125 MHz, $CDCl_3$, 298 K): δ = 156.20, 143.52, 142.32, 141.41, 133.50, 132.44, 128.81, 128.6, 127.78, 127.08, 125.27, 120.02, 118.99, 116.6, 68.05, 47.21 ppm.

IR (ATR): $\tilde{\nu}$ = 3360 (w), 3065 (w), 1696 (m), 1620 (w), 1525 (m), 1501 (w), 1450 (w), 1403 (w), 1340 (w), 1297 (m), 1271 (w), 1157 (w), 1137 (w), 1044 (w), 987 (w), 739 (s), 692 (w), 620 (w), 535 (w) cm^{-1}.

MS (ESI HR, $CHCl_3$/MeOH): *m/z* ($C_{28}H_{22}IN_3O_4+H^+$) = calc.: 592.07278, found: 592.07234 ± 0.73 ppm.

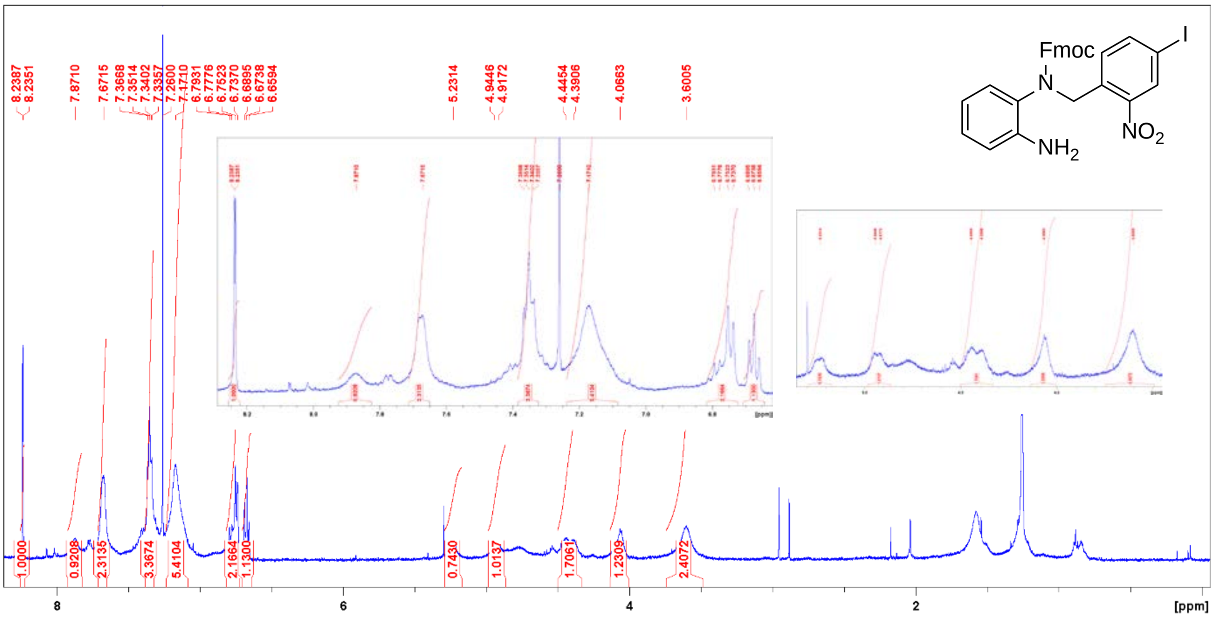

Figure S28: ^{1}H NMR spectrum of compound **6b**.

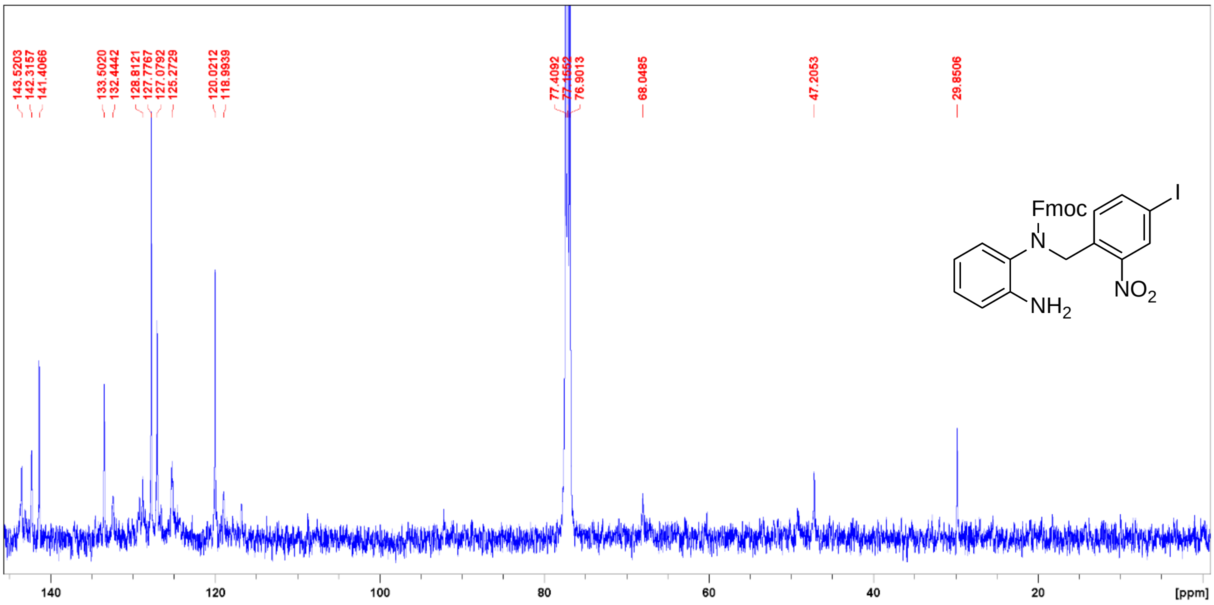

Figure S29: ^{13}C NMR spectrum of compound **6b**.

II.16 Synthesis of (9*H*-fluorene-9-yl)methyl (*Z*)-3-iododibenzo[*c*,*g*][1,2,5]triazocine-11(12*H*)-carboxylate (8b)

(9*H*-Fluorene-9-yl)methyl (2-aminophenyl)(4-iodo-2-nitrobenzyl)carbamate (**6b**, 1.55 g, 2.62mmol) was suspended in 250 mL of ethanol and $SnCl_2{\cdot}2\ H_2O$ (2.96 g, 14.9mmol) was added. The reaction mixture was heated to 75 °C and zink powder (824 mg, 12.6 mmol, 8 eq) was added. It was stirred at rt over night and afterwards neutralized with saturated aqueous $NaHCO_3$ solution. The solvent was evaporated and to the residue was added DCM and deionized water. The organic layer was separated and it was extracted twice with DCM. The combined organic layers were dried over $MgSO_4$ and the solvent was evaporated. The crude product was dissolved in 150 mL of acetic acid and *m*CPBA (1.17 g, 3.15 mmol, ≥ 77%) in 150 mL of acetic acid was added dropwise over a period of 2 h. The reaction mixture was stirred at rt over night and after that, the solvent was evaporated. To the residue was added DCM and half-concentrated aqueous $NaHCO_3$ solution in deionized water and the organic layer was separated. It

was extracted twice with DCM and the combined organic layers were dried over $MgSO_4$. The solvent was evaporated and flash column chromatography on silica (0.040–0.063 mm, ethyl acetate/cyclohexane 1:4, R_f = 0.41) gave a yellow solid (**8b**, 833 mg, 1.49 mmol, 56%).

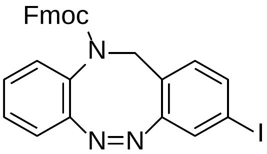

melting point: 87.6 °C

^{1}H NMR (600 MHz, acetone-d_6, 298 K): δ = 8.15-6.65 (m, 15 H, Ar-*H*), 5.05-3.99 (m, 5 H, aliph. *H*) ppm.

^{13}C NMR (150 MHz, acetone-d_6, 298 K): δ = 154.71, 153.87, 144.72, 142.08, 137.37, 132.64, 130.47, 129.54, 129.13, 128.53, 128.36, 127.89, 126.12, 124.68, 120.78, 119.69, 68.50, 52.85, 47.74 ppm. ppm.

IR (ATR): $\tilde{\nu}$ = 2925 (w), 2850 (w),2036 (w), 1703 (s), 1586 (w), 1477 (w), 1449 (m), 1390 (m), 1320 (s), 1295 (s), 1230 (m), 1129 (m), 1105 (m), 1041 (m), 1022 (m), 863 (w), 816 (w), 756 (s), 738 (s), 693 (w), 621 (m), 603 (w), 549 (m), 512 (w) cm^{-1}.

MS (ESI HR, $CHCl_3$/MeOH): *m/z* ($C_{28}H_{20}IN_3O_2+H^+$) = calc.: 558.06730, found: 558.06694 ± 0.63 ppm.

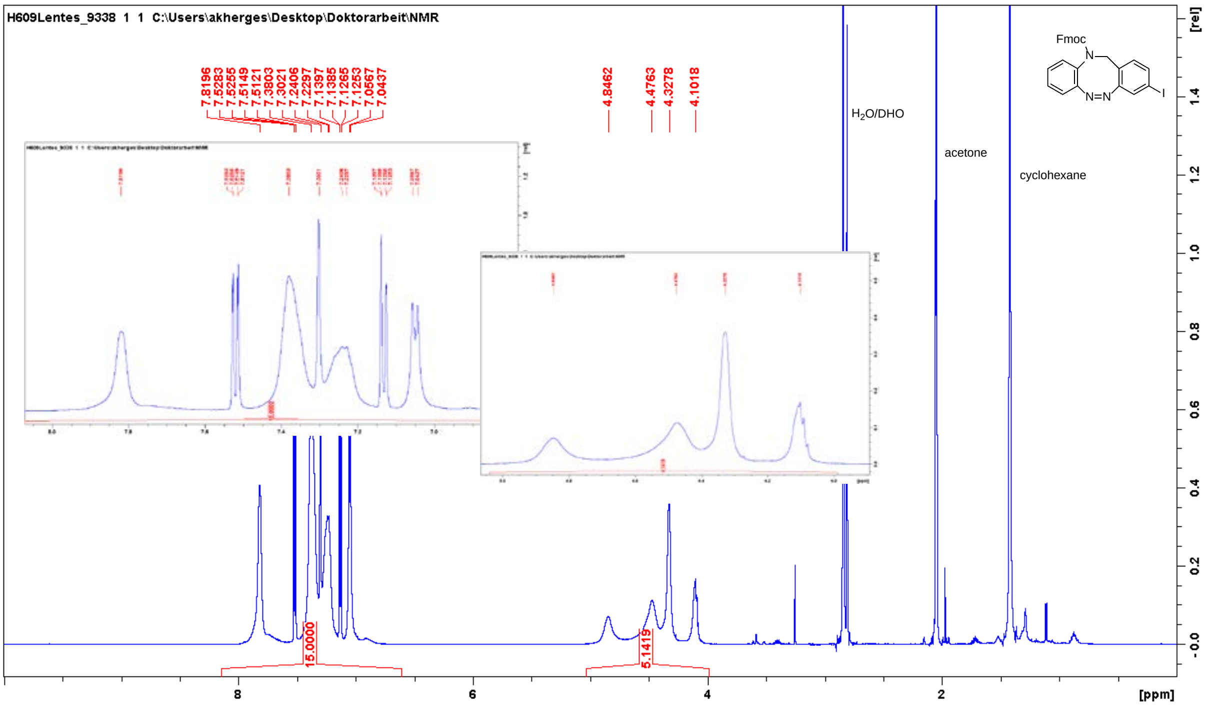

Figure S30: ^{1}H NMR spectrum of compound **8b**.

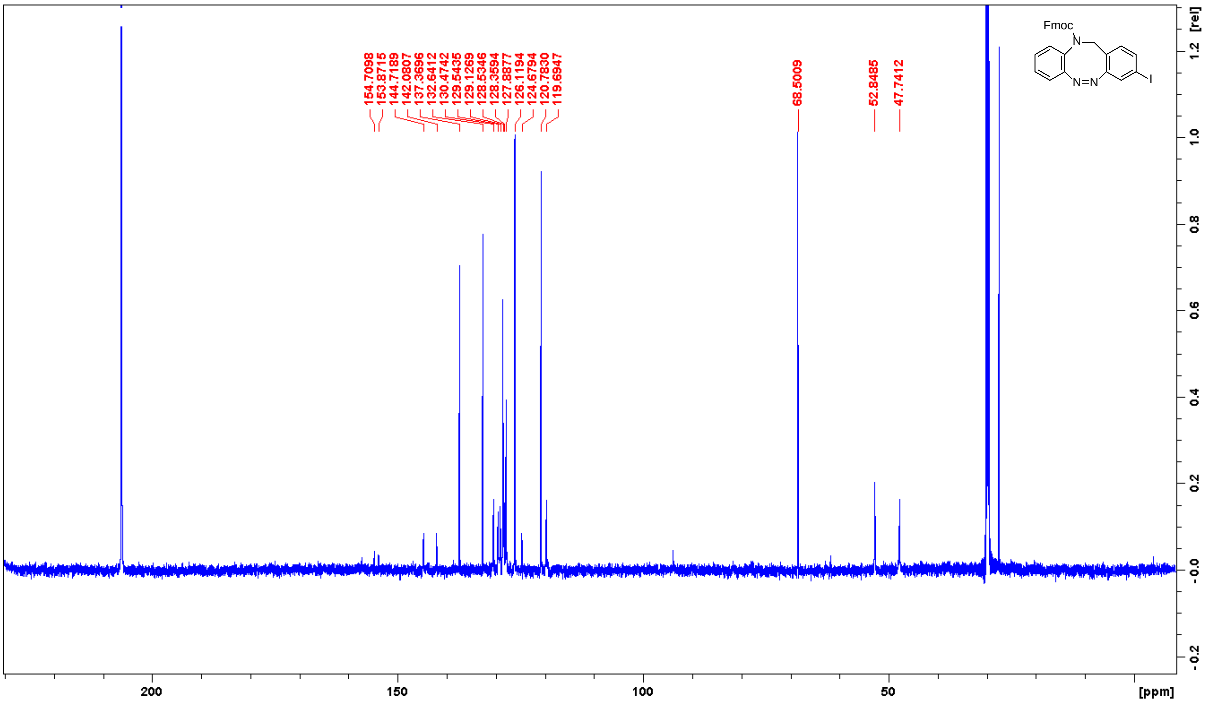

Figure S31: ^{13}C NMR spectrum of compound **8b**.

II.17 Synthesis of (*Z*)-3-iodo-11,12-dihydrodibenzo[*c*,*g*][1,2,5]triazocine (9b)

(9*H*-Fluorene-9-yl)methyl (*Z*)-3-iododibenzo[*c*,*g*][1,2,5]triazocine-11(12*H*)-carboxylate (**8b**, 813 mg, 1.46 mmol) was dissolved in 20 mL of DCM and 50 mL of NEt_3 was added. The reaction mixture was stirred at rt for 20 h. The solvent was removed and flash column chromatography on silica (0.040–0.063 mm, ethyl acetate/cyclohexane 1:3, R_f = 0.43) gave a red solid (**9b**, 410 mg, 1.22 mmol, 84%).

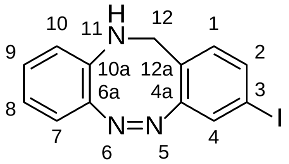

melting point: 165.2 °C

^{1}H NMR (500 MHz, acetone-d$_6$, 298 K): δ = 7.63 (dd, ^3J = 8.0 Hz, ^4J = 1.7 Hz, 1 H, *H*-2), 7.52 (d, ^4J = 1.7 Hz, 1 H, *H*-4), 7.16 (d, ^3J = 8.0 Hz, 1 H, *H*-1), 6.87 (td, ^3J = 7.6 Hz, ^4J = 1.6 Hz, 1 H, *H*-9), 6.75 (dd, ^3J = 8.0 Hz, ^4J = 1.5 Hz, 1 H, *H*-7), 6.65 (td, ^3J = 7.5 Hz, ^4J = 1.2 Hz, 1 H, *H*-8), 6.56 (dd, ^3J = 8.2 Hz, ^4J = 1.1 Hz, 1 H, *H*-10), 5.43 (m_c, 1 H, N*H*), 3.96 (m_c, 2 H, C*H*$_2$) ppm.

^{13}C NMR (125 MHz, acetone-d$_6$, 298 K): δ = 160.26 (*C*-4a), 144.64 (*C*-6a), 137.88 (*C*-2), 137.08 (*C*-10a), 132.44 (*C*-1), 129.06 (*C*-9), 128.50 (*C*-4), 124.49 (*C*-12a), 123.61 (*C*-7), 119.93 (*C*-10), 118.30 (*C*-8), 93.11 (*C*-3), 47.15 (*C*H$_2$) ppm.

IR (ATR): $\tilde{\nu}$ = 3323 (m), 2959 (w), 1601 (m), 1581 (m), 1561 (w), 1518 (m), 1479 (s), 1453 (m), 1383 (m), 1364 (w), 1322 (m), 1251 (w), 1161 (w), 1139 (w), 1117 (w), 1094 (m), 1068 (w), 949 (w), 896 (m), 831 (s), 806 (s), 764 (m), 742 (s), 720 (s), 701 (w), 645 (w), 600 (s), 559 (w), 511 (m) cm^{-1}.

MS (ESI HR, $CHCl_3$/MeOH): *m/z* ($C_{13}H_{10}IN_3+H^+$) = calc.: 335.99922, found: 335.99888 ± 1.02 ppm.

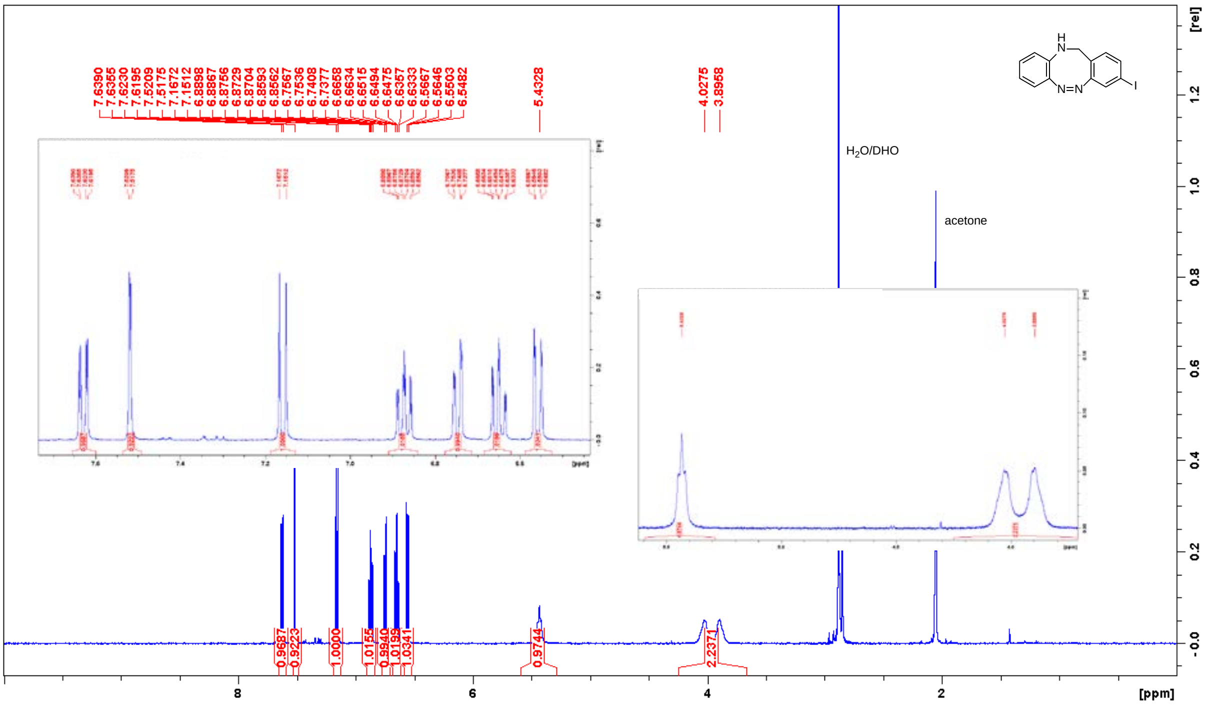

Figure S32: ^{1}H NMR spectrum of compound **9b**.

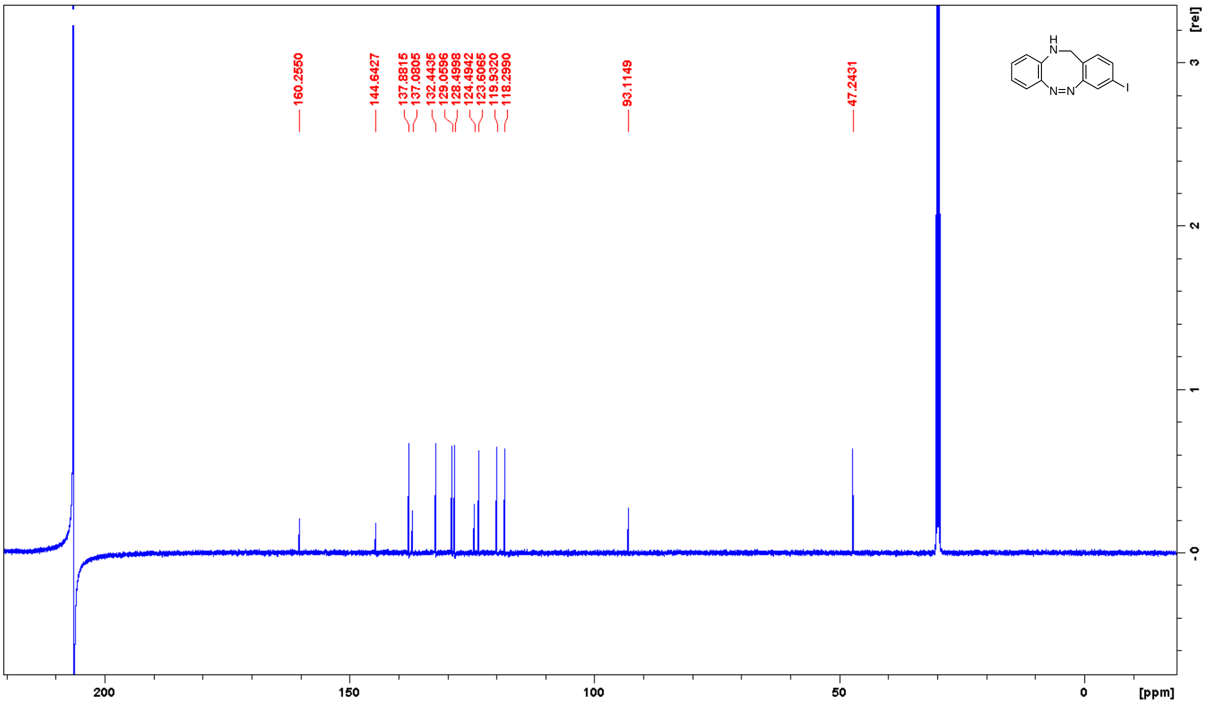

Figure S33: ^{13}C NMR spectrum of compound **9b**.

II.18 Synthesis of (*Z*)-3-iododibenzo[*c*,*g*][1,2,5]triazocine-11(12*H*)-carbaldehyde (11b)

Under a nitrogen atmosphere, a solution of (*Z*)-3-iodo-11,12-dihydrodibenzo[*c*,*g*][1,2,5]triazocine (**9b**, 50.0 mg, 149 µmol) in anhydrous MeCN (5 mL) was prepared, and then DIPEA (260 µL, 1.49 mmol) and chloral (146 µL, 1.49 mmol) were added. The reaction mixture was stirred at rt for 22 h. The solvent

was removed and flash column chromatography on silica (0.040–0.063 mm, ethyl acetate/cyclohexane 1:2, R_f = 0.20) gave a pale yellow solid (**11b**, 28 mg, 77.1 µmol, 52%).

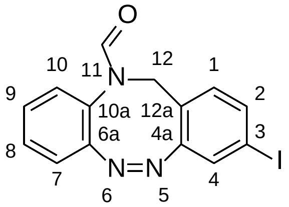

melting point: 208.9 °C

^{1}H NMR (500 MHz, acetone-d$_6$, 298 K): δ = 8.35 (s, 1 H, C*H*O), 7.58 (dd, ^3J = 8.0 Hz, ^4J = 1.7 Hz, 1 H, *H*-2), 7.41 (d, ^4J = 1.7 Hz, 1 H, *H*-4), 7.36 (td, ^3J = 7.7 Hz, ^4J = 1.3 Hz, 1 H, *H*-9), 7.26 (td, ^3J = 7.5 Hz, ^4J = 1.5 Hz, 1 H, *H*-8), 7.18 (dd, ^3J = 7.9 Hz, ^4J = 1.3 Hz, 1 H, *H*-7), 7.15 (d, ^3J = 8.0 Hz, 1 H, *H*-1), 6.96 (dd, ^3J = 7.8 Hz, ^4J = 1.4 Hz, 1 H, *H*-10), 4.50 (m$_c$, 2 H, C*H*$_2$) ppm.

^{13}C NMR (125 MHz, acetone-d$_6$, 298 K): δ = 162.37 (*C*=O), 158.26 (*C*-4a), 152.78 (*C*-6a), 137.80 (*C*-2), 133.25 (*C*-1), 129.44 (*C*-8), 129.28 (*C*-9), 128.28 (*C*-7), 128.19 (*C*-12a), 127.93 (*C*-4), 123.30 (*C*-10a), 120.54 (*C*-10), 94.57 (*C*-3), 49.12 (*C*H$_2$) ppm.

IR (ATR): $\tilde{\nu}$ = 3070 (w), 3045 (w), 2880 (w), 1935 (w), 1668 (s), 1538 (m), 1513 (w), 1475 (m), 1387 (w), 1358 (m), 1313 (s), 1277 (m), 1242 (m), 1209 (w), 1117 (m), 1038 (m), 1012 (w), 956 (m), 028 (w), 892 (w), 828 (s), 810 (s), 759 (s), 741 (m), 692 (m), 666 (m), 647 (w), 605 (w), 562 (s), 516 (m) cm^{-1}.

MS (ESI HR, CHCl$_3$/MeOH): *m/z* ($C_{14}H_{10}IN_3O+H^+$) = calc.: 363.99413, found: 363.99426 ± 0.36 ppm.

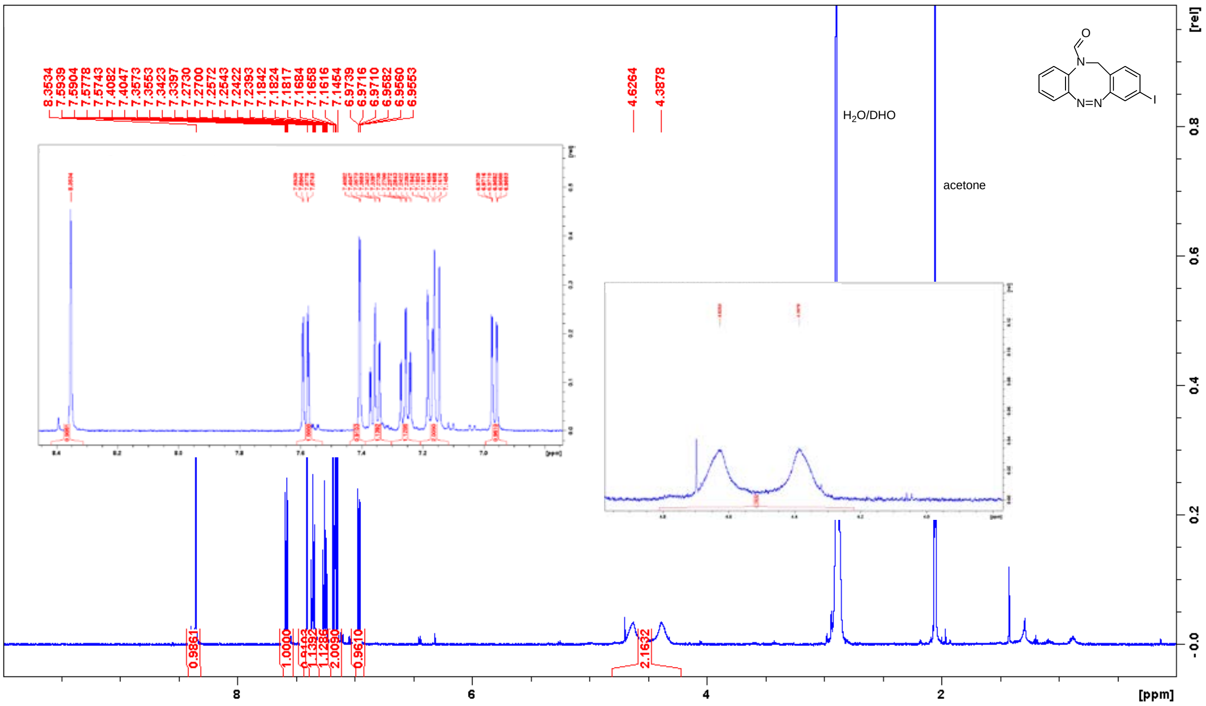

Figure S34: ^{1}H NMR spectrum of compound **11b**.

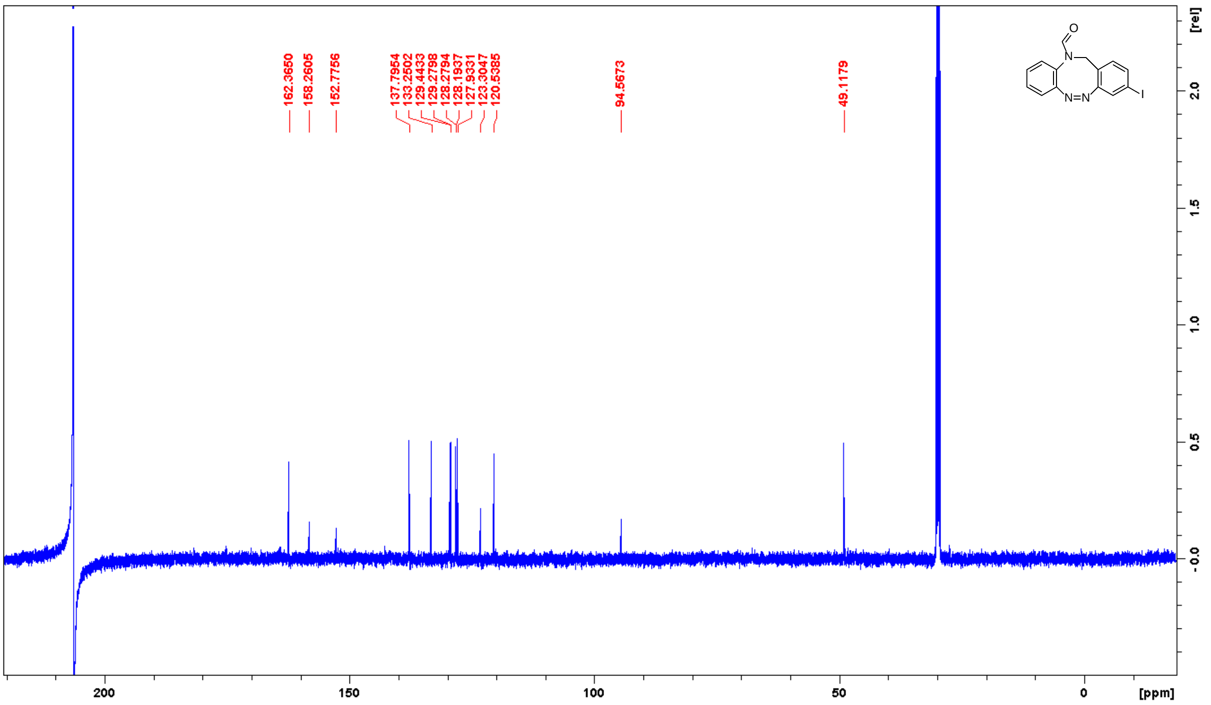

Figure S35: ^{13}C NMR spectrum of compound **11b**.

II.19 Synthesis of (*Z*)-1-(3-iododibenzo[*c*,*g*][1,2,5]triazocine-11(12*H*)-yl)ethan-1-one (10b)

Under a nitrogen atmosphere, a solution of (*Z*)-3-iodo-11,12-dihydrodibenzo[*c*,*g*][1,2,5]triazocine (**9b**, 100 mg, 298 µmol) in anhydrous DMF (5 mL) was prepared, and then TEA (832 µL, 20 equiv) and HOAcl (171 µL, 2.98 mmol) were added. The reaction mixture was cooled to 0 °C and T3P (1.78 mL, 2.98 mmol, 50% in ethyl acetate) was added dropwise. The ice bath was removed and it was stirred for 21 h. Then, 50 mL DCM and 50 mL deionized water were added and the organic layer was separated. It was extracted twice with DCM and the combined organic layers were dried over $MgSO_4$. The solvent was removed and flash column chromatography on silica (0.040–0.063 mm, ethyl acetate/cyclohexane 1:2, R_f = 0.13) gave a pale yellow solid (**10b**, 68 mg, 180 µmol, 60%).

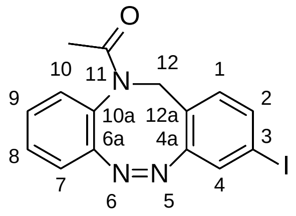

melting point: 170.0 °C

^{1}H NMR (500 MHz, acetone-d$_6$, 298 K): δ = 7.51 (dd, ^3J = 8.1 Hz, ^4J = 1.8 Hz, 1 H, *H*-2), 7.51 (td, ^3J = 7.4 Hz, ^4J = 1.8 Hz, 1 H, *H*-8/9), 7.34-7.26 (m, 3 H, *H*-4, Ar-*H*, Ar-*H*), 7.06 (dd, ^3J = 8.0 Hz, ^4J = 1.3 Hz, 1 H, *H*-7/10), 7.03 (d, ^3J = 8.1 Hz, 1 H, *H*-1), 5.03 (d, ^2J = 14.5 Hz, 1 H, CH_2a), 4.30 (d, ^2J = 14.9 Hz, 1 H, CH_2b), 1.79 (s, 3 H, CH_3) ppm.

^{13}C NMR (125 MHz, acetone-d$_6$, 298 K): δ = 169.50 (*C*=O), 157.09 (*C*-4a), 154.15 (*C*-6a/10a), 137.40 (*C*-2), 132.73 (*C*-1), 130.32 (*C*-7/10), 130.19 (Ar-*C*), 129.58 (Ar-*C*), 129.44 (*C*-6a/10a), 128.43 (*C*-4), 125.19 (*C*-12a), 120.05 (*C*-7/10), 93.56 (*C*-6a/10a), 51.65 (*C*H_2), ppm.

IR (ATR): $\tilde{\nu}$ = 3058 (w), 3012 (w), 2928 (w), 2251 (w), 1652 (s), 1583 (w), 1556 (w), 1513 (w), 1473 (m), 1434 (w), 1378 (s), 1336 (s), 1297 (m), 1250 (w), 1111 (m), 1084 (m), 1031 (m), 1009 (w), 971 (m), 917 (w), 842 (m), 824 (s), 768 (s), 691 (m), 653 (m), 598 (s), 582 (s), 534 (w), 515 (m) cm^{-1}.

MS (ESI HR, $CHCl_3$/MeOH): *m/z* ($C_{15}H_{12}IN_3O+H^+$) = calc.: 378.00978, found: 378.00937 ± 1.08 ppm.

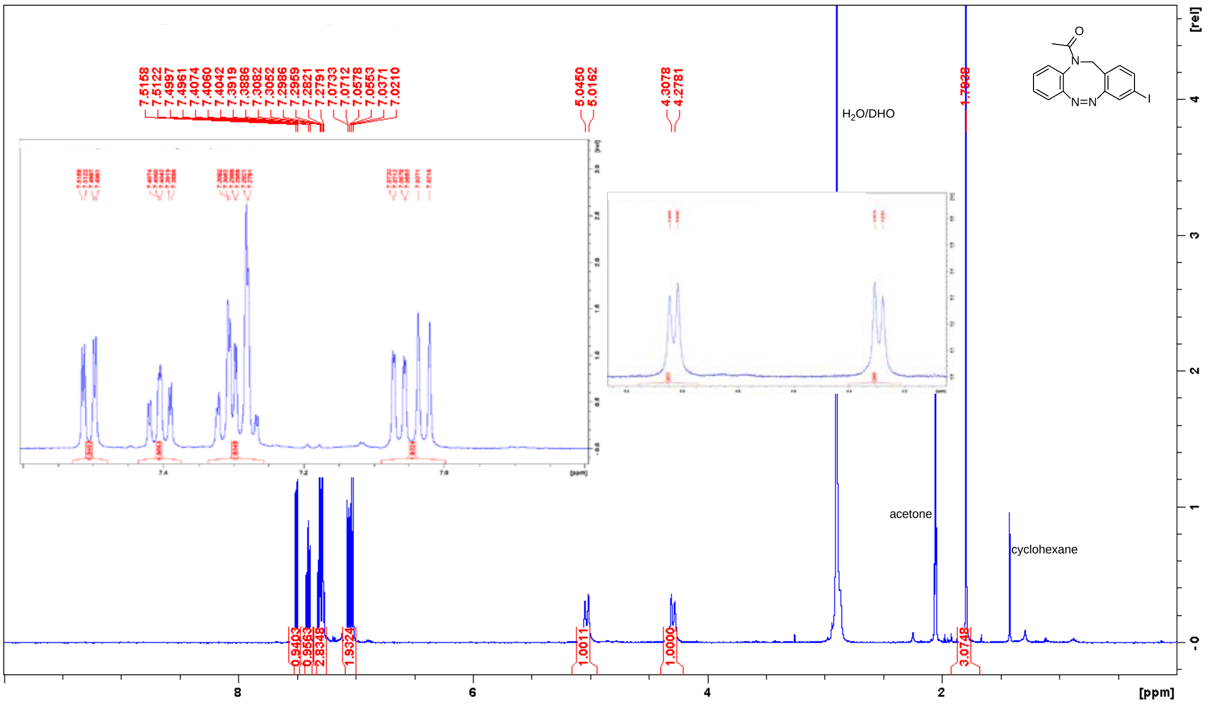

Figure S36: ^{1}H NMR spectrum of compound **10b**.

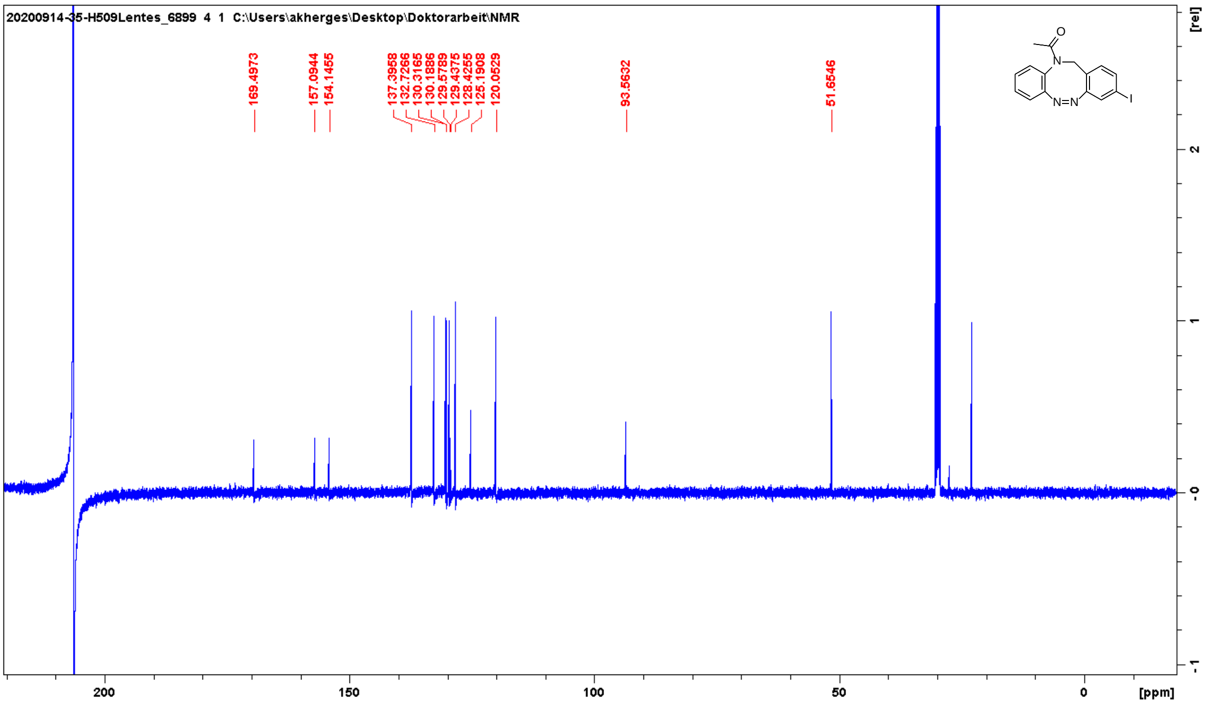

Figure S37: ^{13}C NMR spectrum of compound **10b**.

III. UV–vis switching experiments

All samples were irradiated to the photostationary state (PSS) with 400 nm in the given solvent or solvent mixture. The extrapolated UV–vis spectra of the *E* compounds were calculated from the UV–vis spectra of the PSS and the percentage of the *E* compounds which were measured by ^{1}H NMR (for details see SIV) by the following equation (1):

$$A_{E,\text{extrapol.}} = \left\{A_{PSS} - \left(A_Z \cdot \left[1 - \frac{100\%}{\%\text{PSS}}\right]\right)\right\} / \left(\frac{100\%}{\%\text{PSS}}\right) \tag{1}$$

The rate constants *k* of the thermal *E*→*Z* isomerization of all compounds were determined with first oder kinetic plots by the following equation (2):

$$A_t = A_0 \cdot e^{-kt} \tag{2}$$

Half-lifes ($t_{1/2}$) were calculated with equation 3:

$$t_{1/2} = \frac{\ln(2)}{k} \tag{3}$$

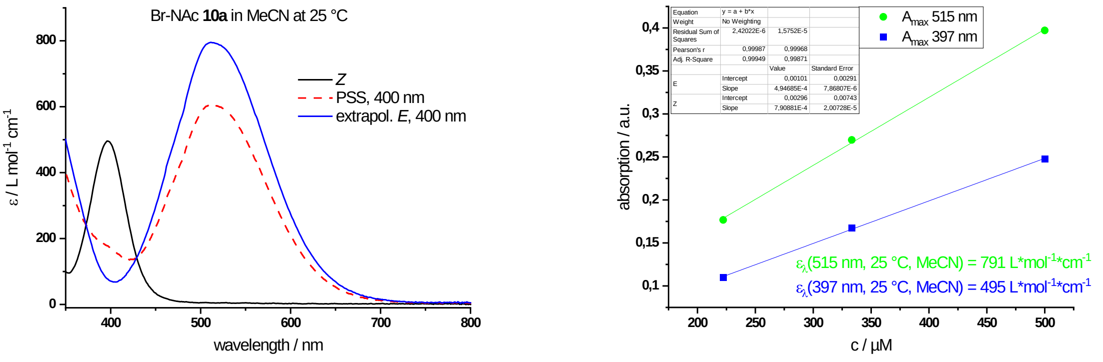

Figure S38: UV–vis spectra of Br-NAc-diazocine **10a** in MeCN at 25 °C before (black) and after irradiation with 400 nm (dashed red). The extrapolated *E* spectrum is given in blue (left) and molar extinction coefficient (ε) determination of the maxiumum absorption (λ_{max}) of the $n\pi^*$-transitions of the *E* (green) and *Z* (blue) isomers (right).

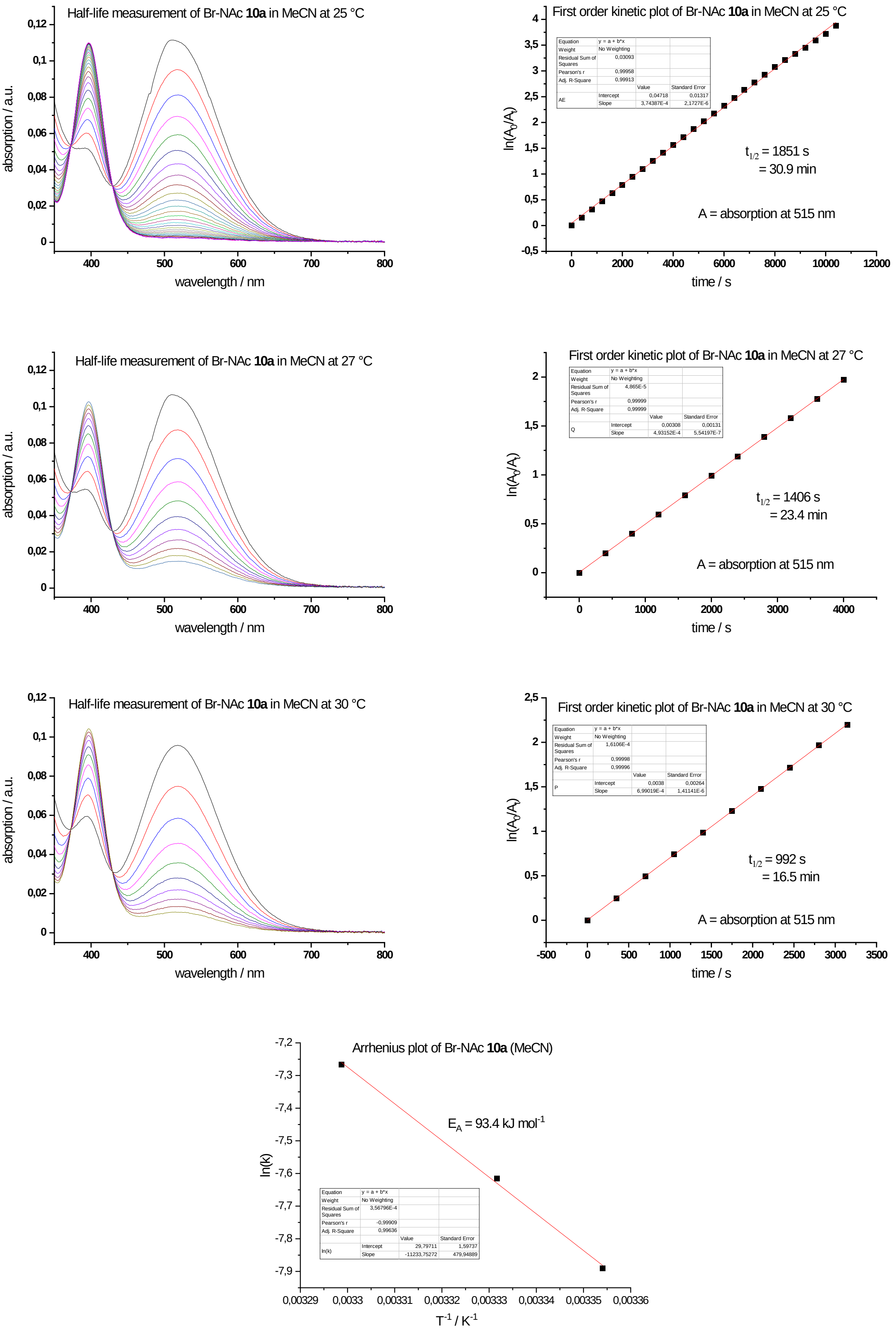

Figure S39: UV–vis spectra of the half-life determination of Br-NAc-diazocine **10a** in MeCN at different temperatures (left) with corresponding first order kinetic plots (right) and Arrhenius plot (bottom).

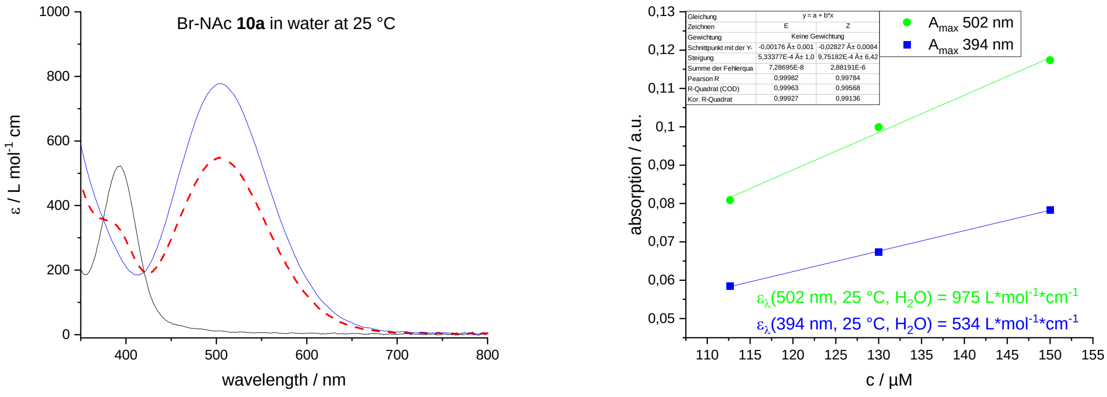

Figure S40: UV–vis spectra of Br-NAc-diazocine **10a** in water at 25 °C before (black) and after irradiation with 400 nm (dashed red). The extrapolated *E* spectrum is given in blue (left) and molar extinction coefficient (ε) determination of the maxiumum absorption (λ_{max}) of the $n\pi^*$-transitions of the *E* (green) and *Z* (blue) isomers (right).

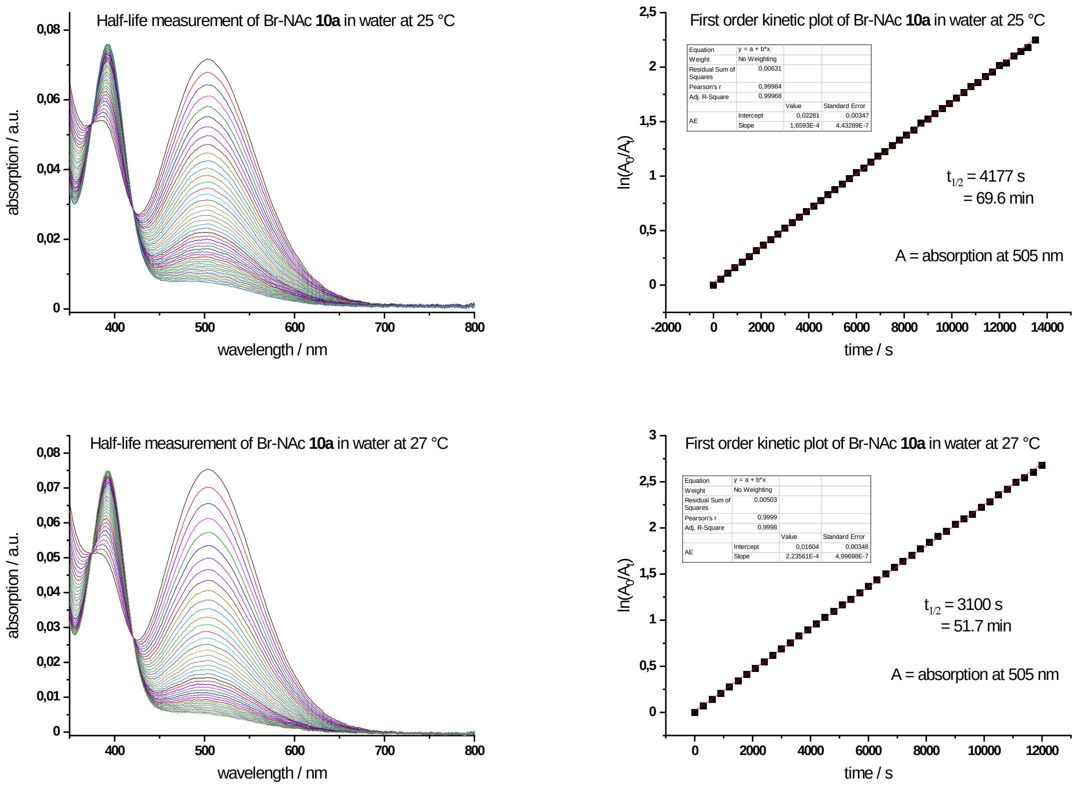

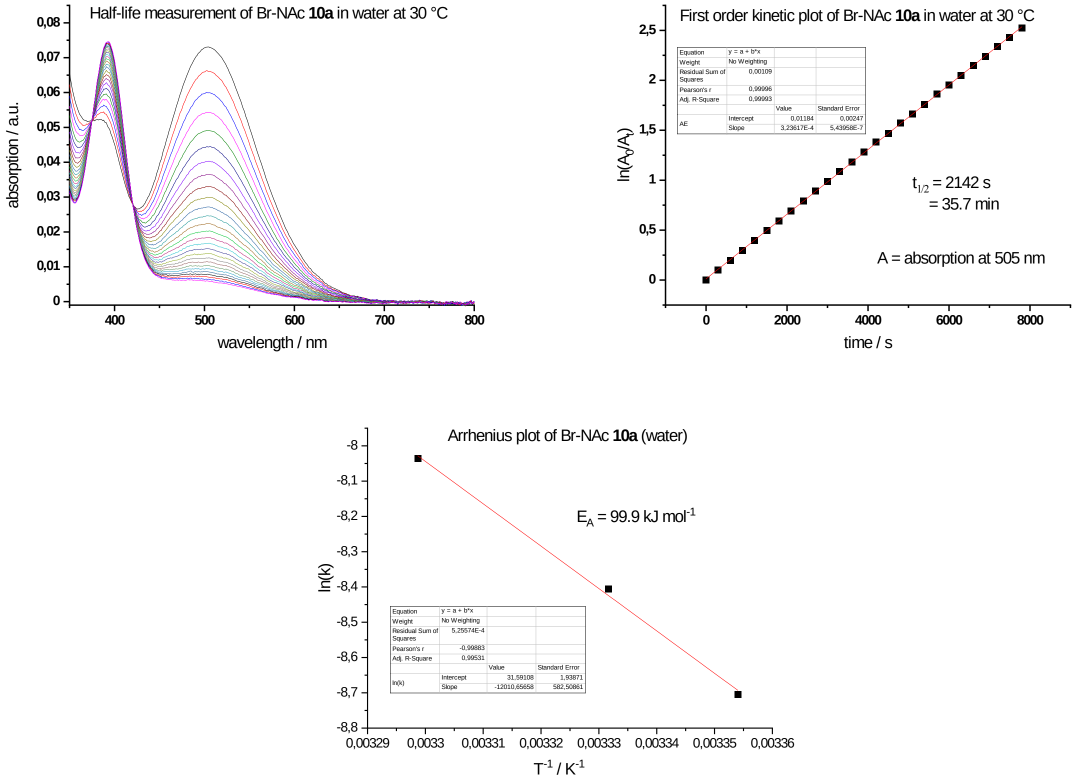

Figure S41: UV–vis spectra of the half-life determination of Br-NAc-diazocine **10a** in water at different temperatures (left) with corresponding first order kinetic plots (right) and Arrhenius plot (bottom).

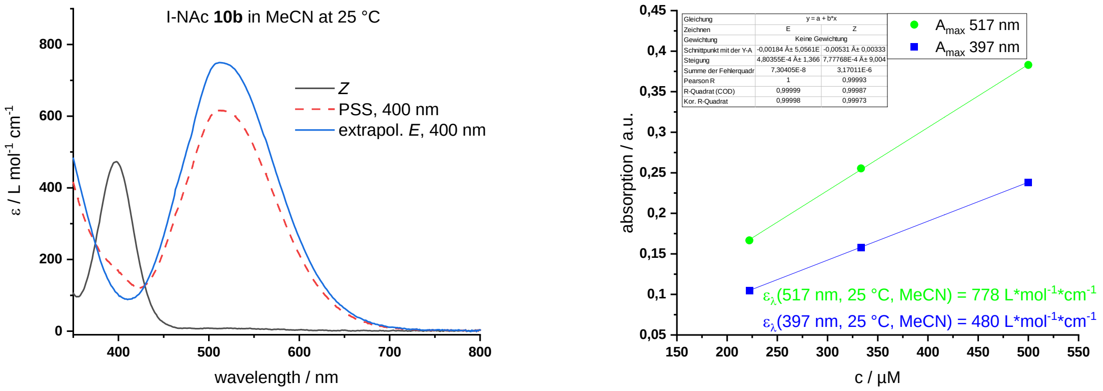

Figure S42: UV–vis spectra of I-NAc-diazocine **10b** in MeCN at 25 °C before (black) and after irradiation with 400 nm (dashed red). The extrapolated *E* spectrum is given in blue (left) and molar extinction coefficient (ε) determination of the maxiumum absorption (λ_{max}) of the $n\pi^*$-transitions of the *E* (green) and *Z* (blue) isomers (right).

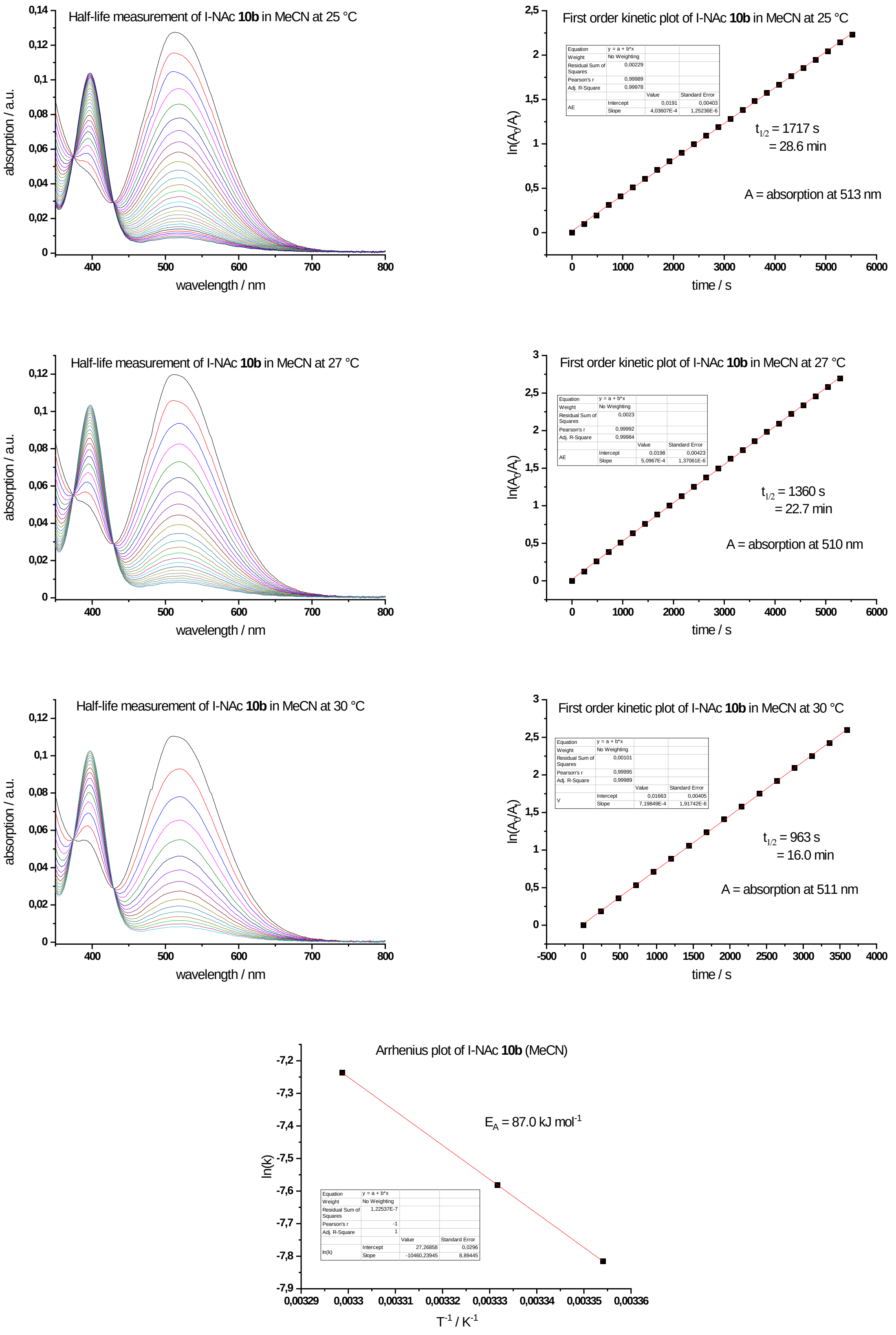

Figure S43: UV–vis spectra of the half-life determination of I-NAc-diazocine **10b** in MeCN at different temperatures (left) with corresponding first order kinetic plots (right) and Arrhenius plot (bottom).

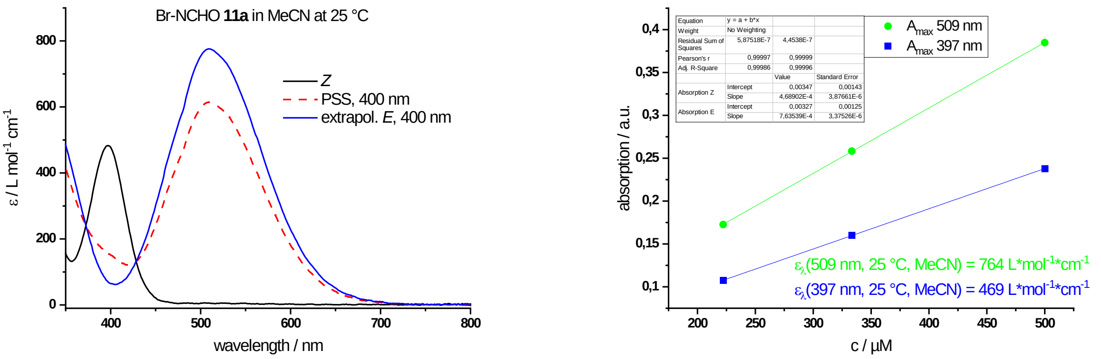

Figure S44: UV–vis spectra of Br-NCHO-diazocine **11a** in MeCN at 25 °C before (black) and after irradiation with 400 nm (dashed red). The extrapolated *E* spectrum is given in blue (left) and molar extinction coefficient (ε) determination of the maxiumum absorption (λ_{max}) of the $n\pi^*$-transitions of the *E* (green) and *Z* (blue) isomers (right).

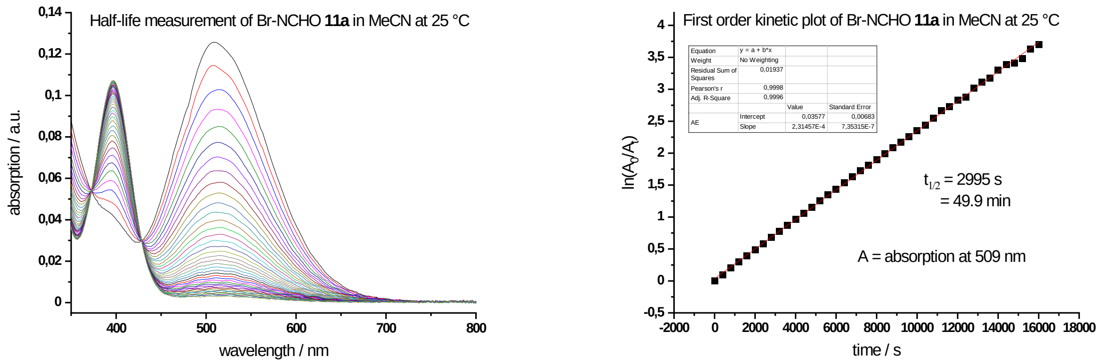

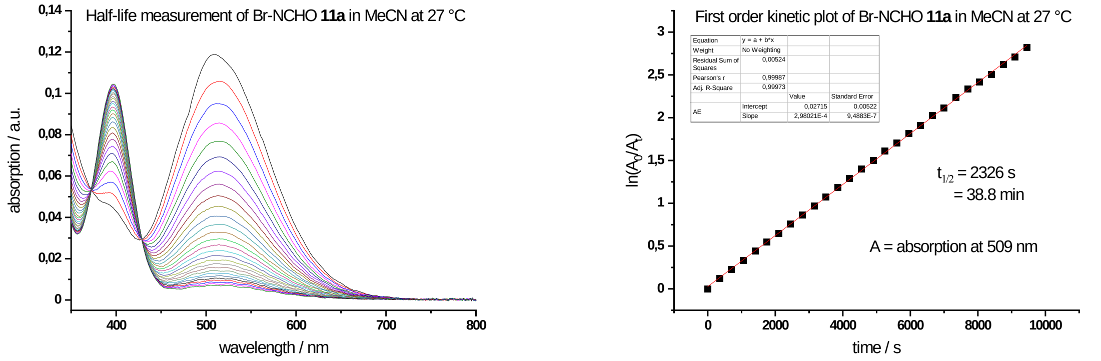

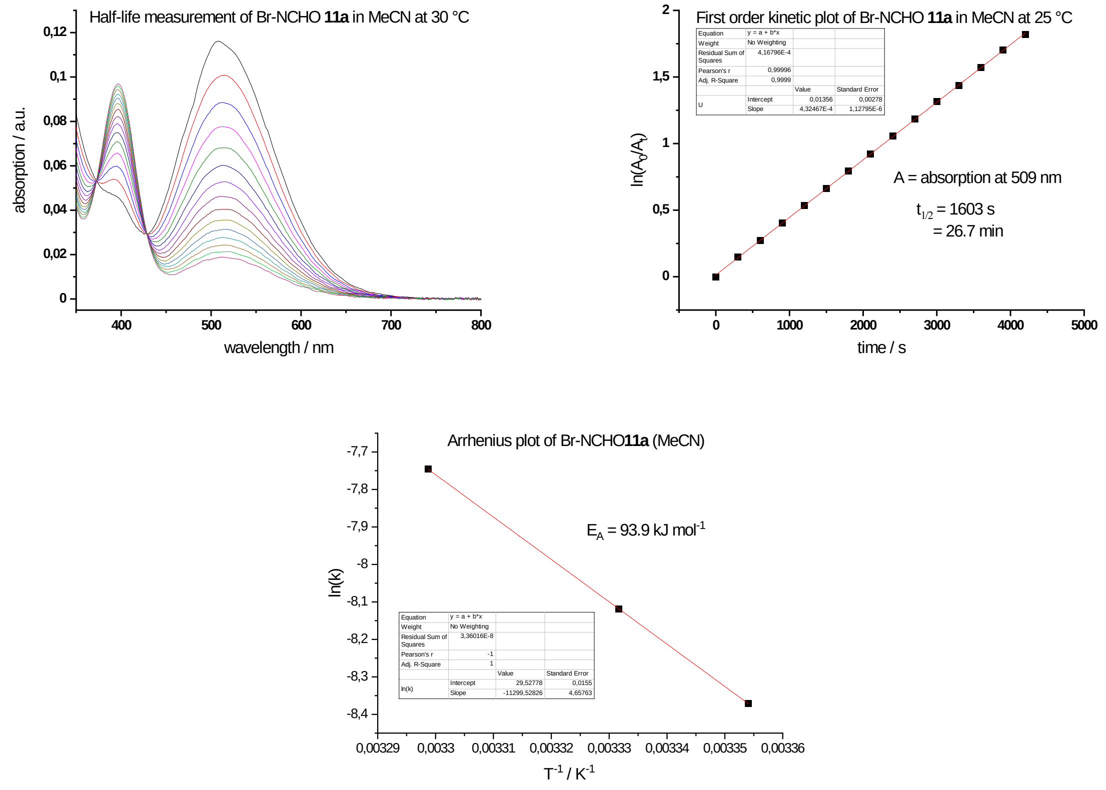

Figure S45: UV–vis spectra of the half-life determination of Br-NCHO-diazocine **11a** in MeCN at different temperatures (left) with corresponding first order kinetic plots (right) and Arrhenius plot (bottom).

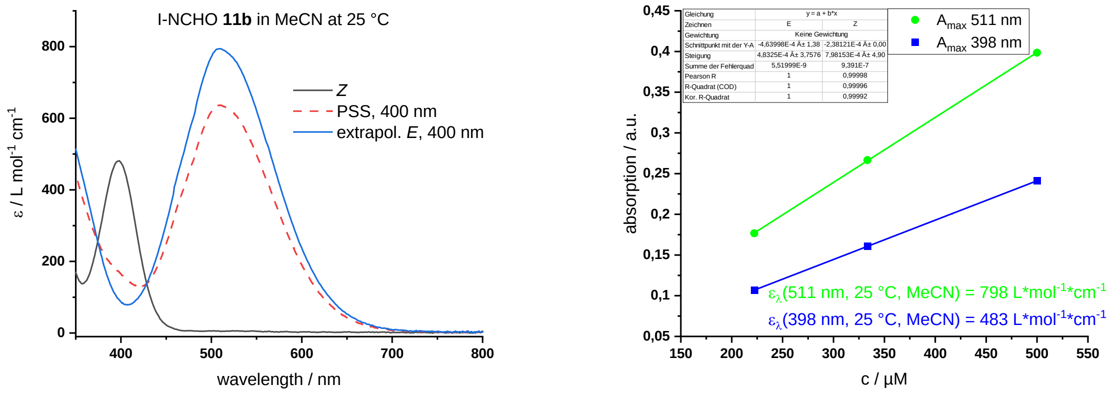

Figure S46: UV–vis spectra of I-NCHO-diazocine **11b** in MeCN at 25 °C before (black) and after irradiation with 400 nm (dashed red). The extrapolated *E* spectrum is given in blue (left) and molar extinction coefficient (ε) determination of the maxiumum absorption (λ_{max}) of the $n\pi$*-transitions of the *E* (green) and *Z* (blue) isomers (right).

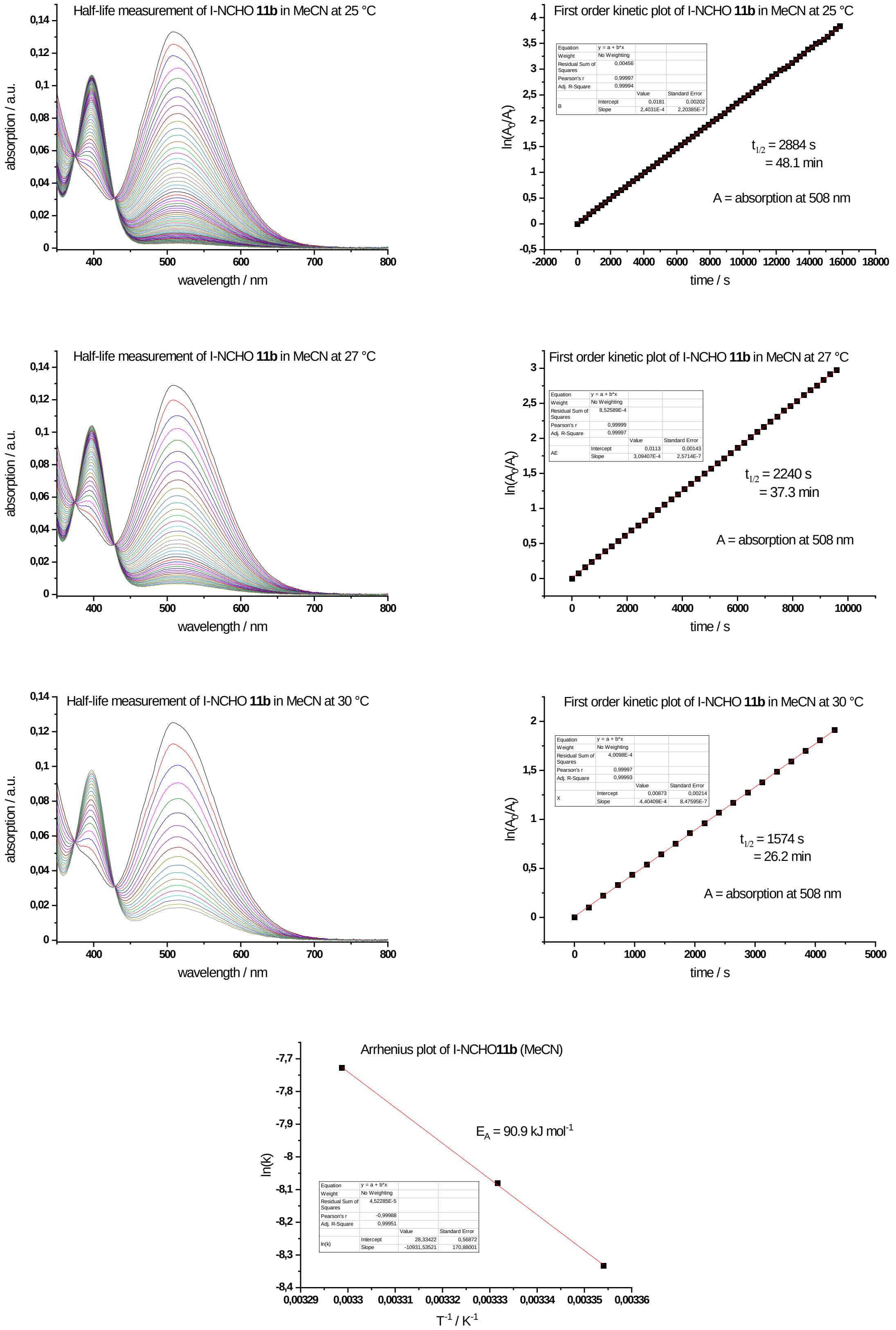

Figure S47: UV–vis spectra of the half-life determination of I-NCHO-diazocine **11b** in MeCN at different temperatures (left) with corresponding first order kinetic plots (right) and Arrhenius plot (bottom).

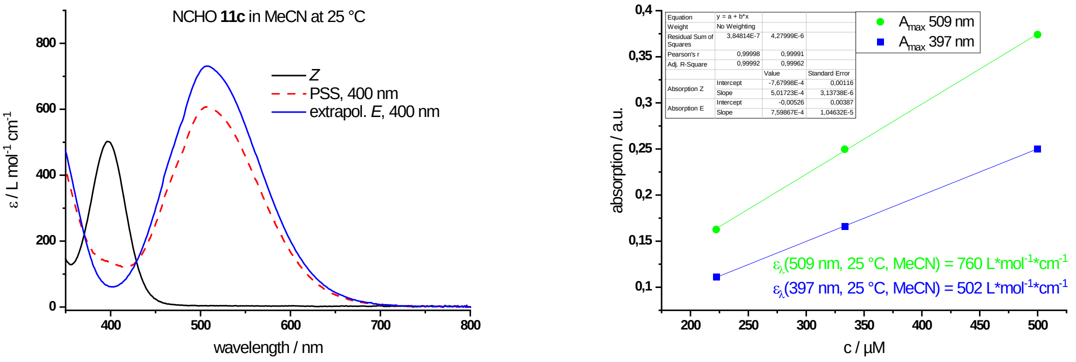

Figure S48: UV–vis spectra of NCHO-diazocine **11c** in MeCN at 25 °C before (black) and after irradiation with 400 nm (dashed red). The extrapolated *E* spectrum is given in blue (left) and molar extinction coefficient (ε) determination of the maxiumum absorption (λ_{max}) of the $n\pi^*$-transitions of the *E* (green) and *Z* (blue) isomers (right).

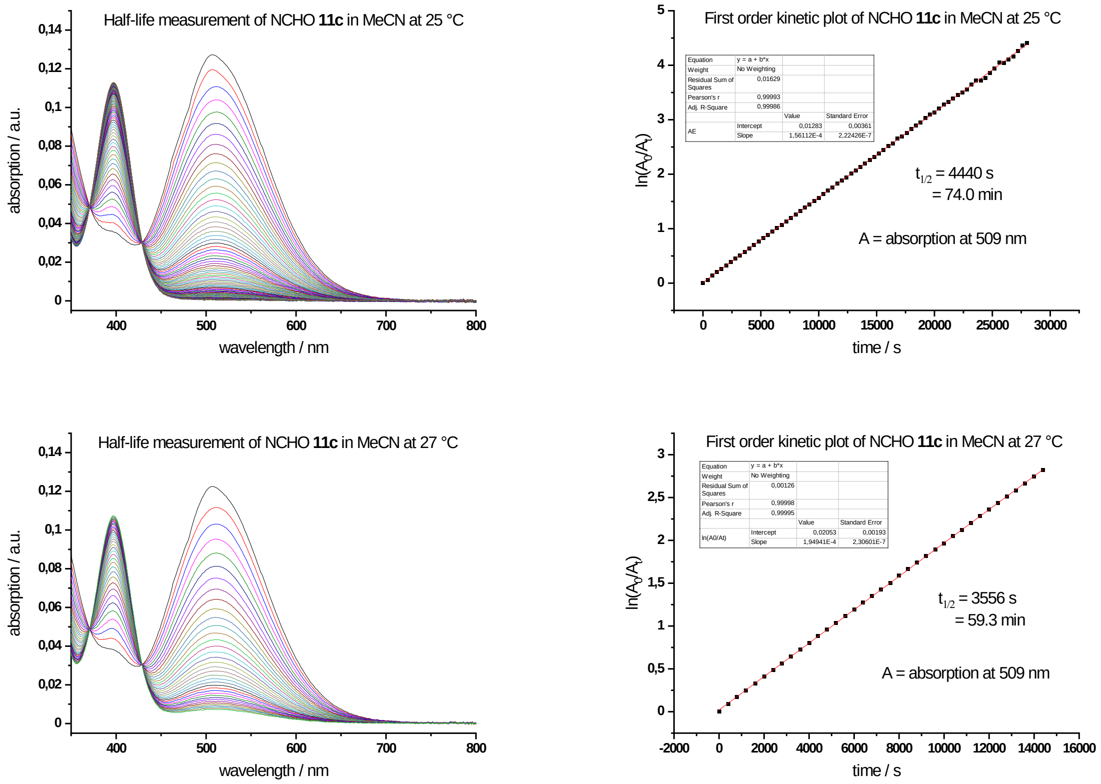

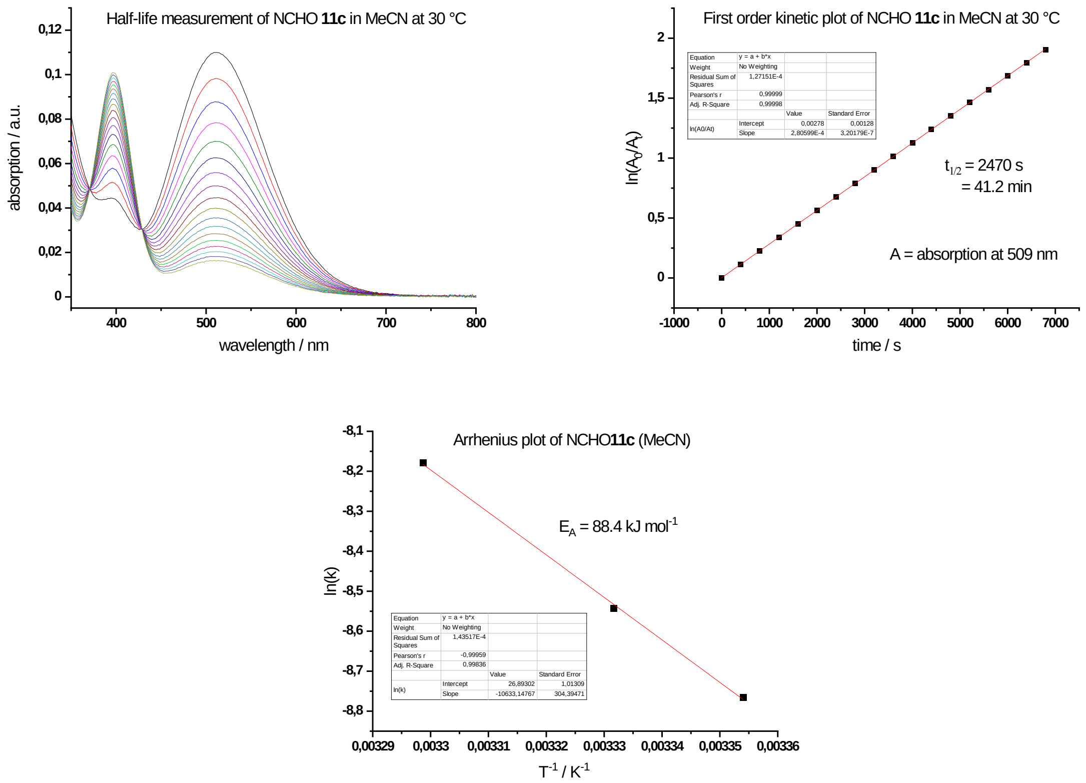

Figure S49: UV–vis spectra of the half-life determination of NCHO-diazocine **11c** in MeCN at different temperatures (left) with corresponding first order kinetic plots (right) and Arrhenius plot (bottom).

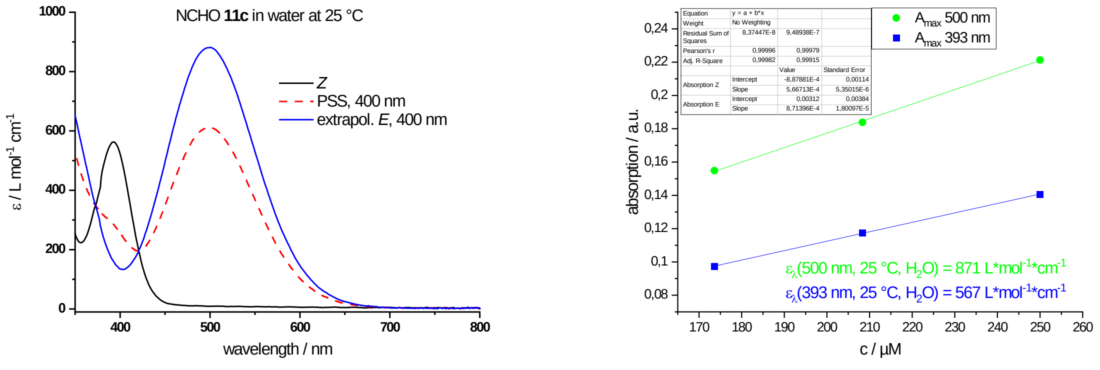

Figure S50: UV–vis spectra of NCHO-diazocine **11c** in water at 25 °C before (black) and after irradiation with 400 nm (dashed red). The extrapolated *E* spectrum is given in blue (left) and molar extinction coefficient (ε) determination of the maxiumum absorption (λ_{max}) of the $n\pi^*$-transitions of the *E* (green) and *Z* (blue) isomers (right).

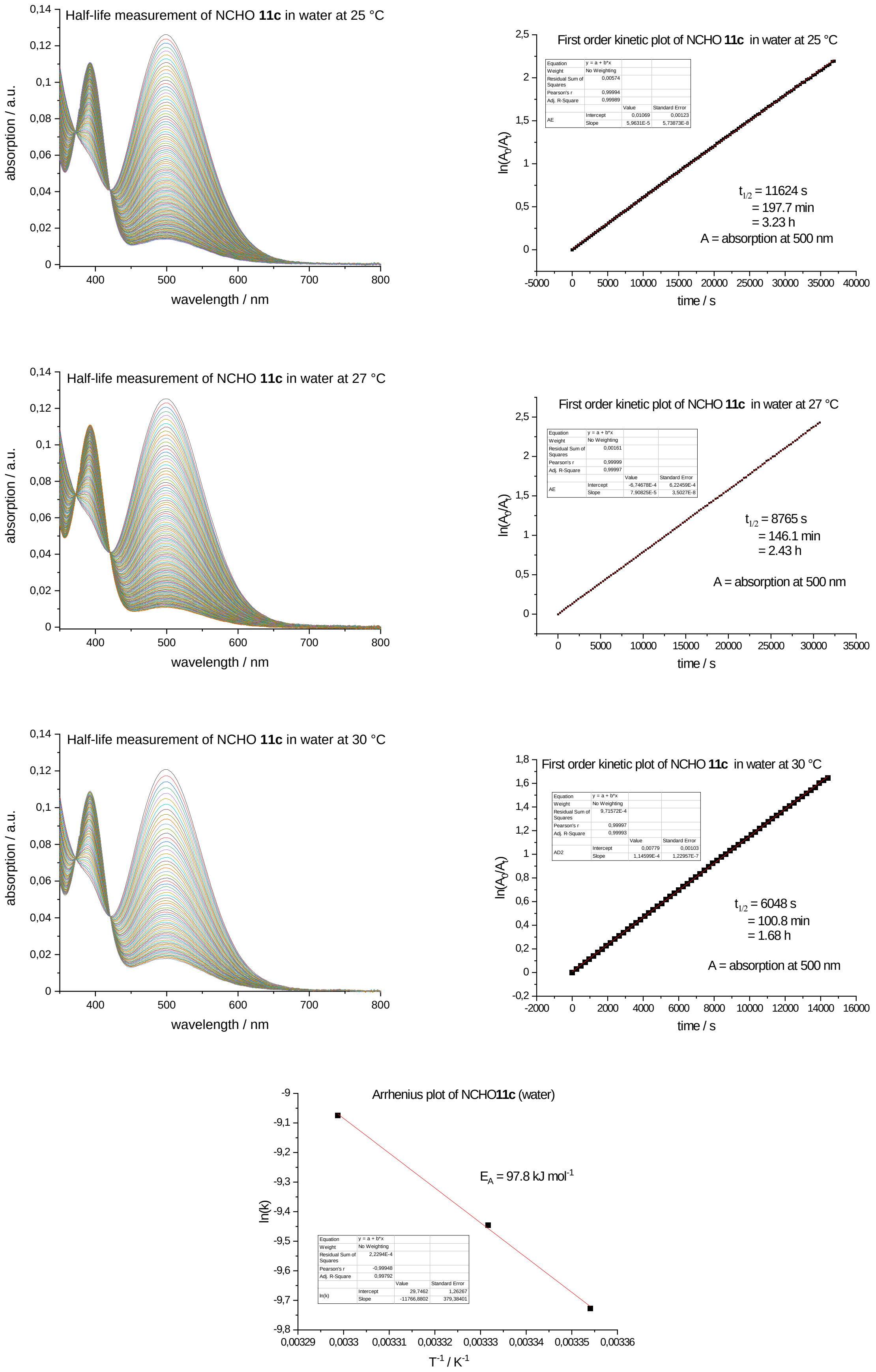

Figure S51: UV–vis spectra of the half-life determination of NCHO-diazocine **11c** in water at different temperatures (left) with corresponding first order kinetic plots (right) and Arrhenius plot (bottom).

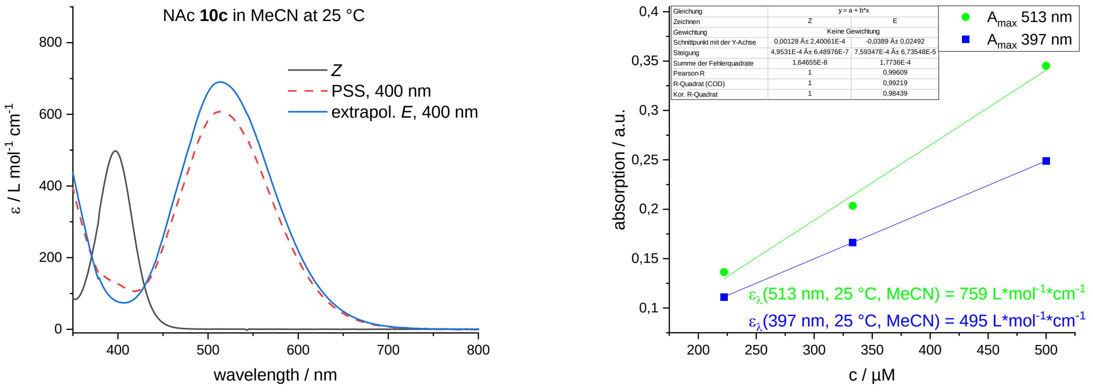

Figure S52: UV–vis spectra of NAc-diazocine **10c** in MeCN at 25 °C before (black) and after irradiation with 400 nm (dashed red). The extrapolated *E* spectrum is given in blue (left) and molar extinction coefficient (ε) determination of the maxiumum absorption (λ_{max}) of the $n\pi$*-transitions of the *E* (green) and *Z* (blue) isomers (right).

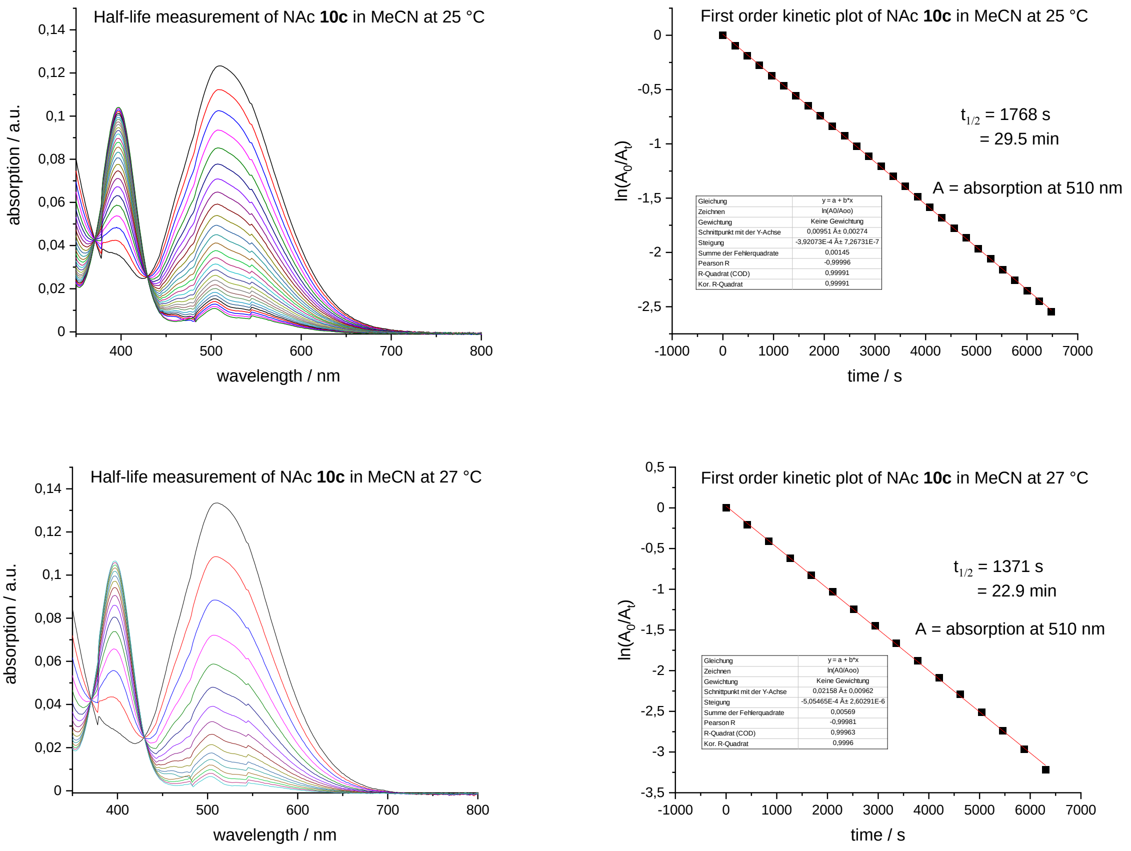

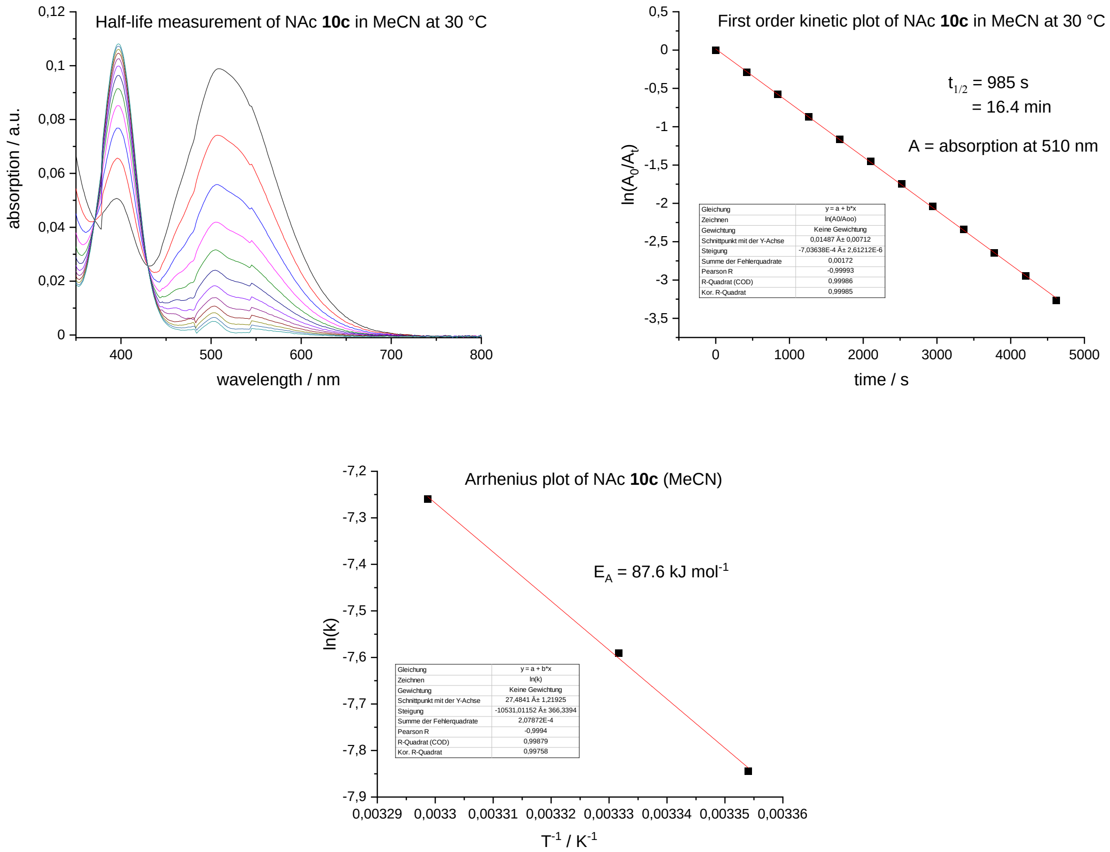

Figure S53: UV–vis spectra of the half-life determination of NAc-diazocine **10c** in MeCN at different temperatures (left) with corresponding first order kinetic plots (right) and Arrhenius plot (bottom).

IV. ^{1}H NMR switching experiments

Photostationary states (PSS) of compounds **10** and **11** were measured via ^{1}H NMR by integration of the different species after irradiation with 400 nm. The value of the integrals and the extrapolated values is given in Table S1. ^{1}H NMR und UV–vis spectra (SIII) were measured at 250 µM concentrations, except of Br-NCHO diazocine **11a** (150 µM due to solubility limit). The following equation was used for extrapolation of the photostationary states:

$$PSS_{\text{extrapol.}} = \text{PSS} \cdot e^{kt} \quad (2)$$

k is the rate constant at 25 °C (see SIII) and *t* the measuring time (180 s): 60 s preparation (transfer into the spectrometer and shimming) and ~120 s measuring time for 32 scans. All spectra were measured at a Bruker AV 600 spectrometer.

Table S1: Measured and extrapolated photostationary states of compounds **10** and **11** in MeCN and water.

	CD_3CN		D_2O	
	measured PSS / %	extrapolated PSS / %	measured PSS / %	extrapolated PSS / %
Br-NAc **10a**	76	81	68	70
I-NAc **10b**	76	82	-	-
NCHO **11c**	83	85	68	69
Br-NCHO **11a**	79	82	-	-
I-NCHO **11b**	77	80	-	-
NAc **10c**	82	88	-	-

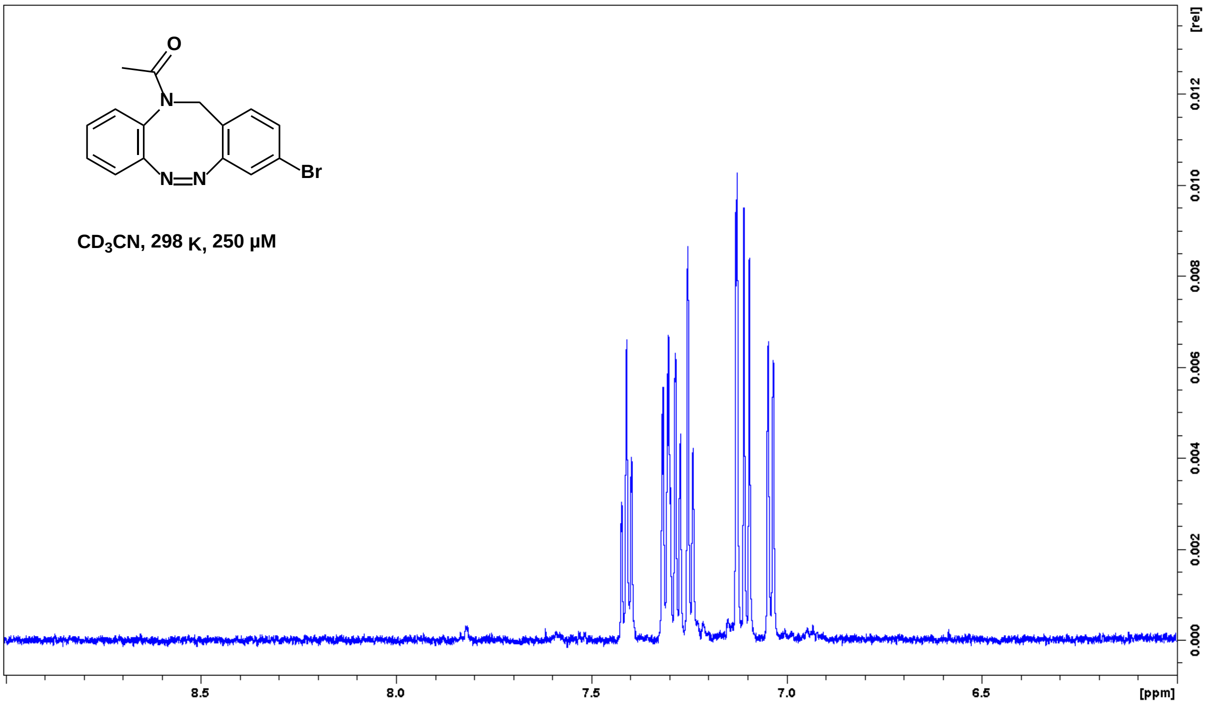

Figure S54: ^1H NMR spectrum (aromatic region) of Br-NAc diazocine **10a** in CD_3CN at 298 K.

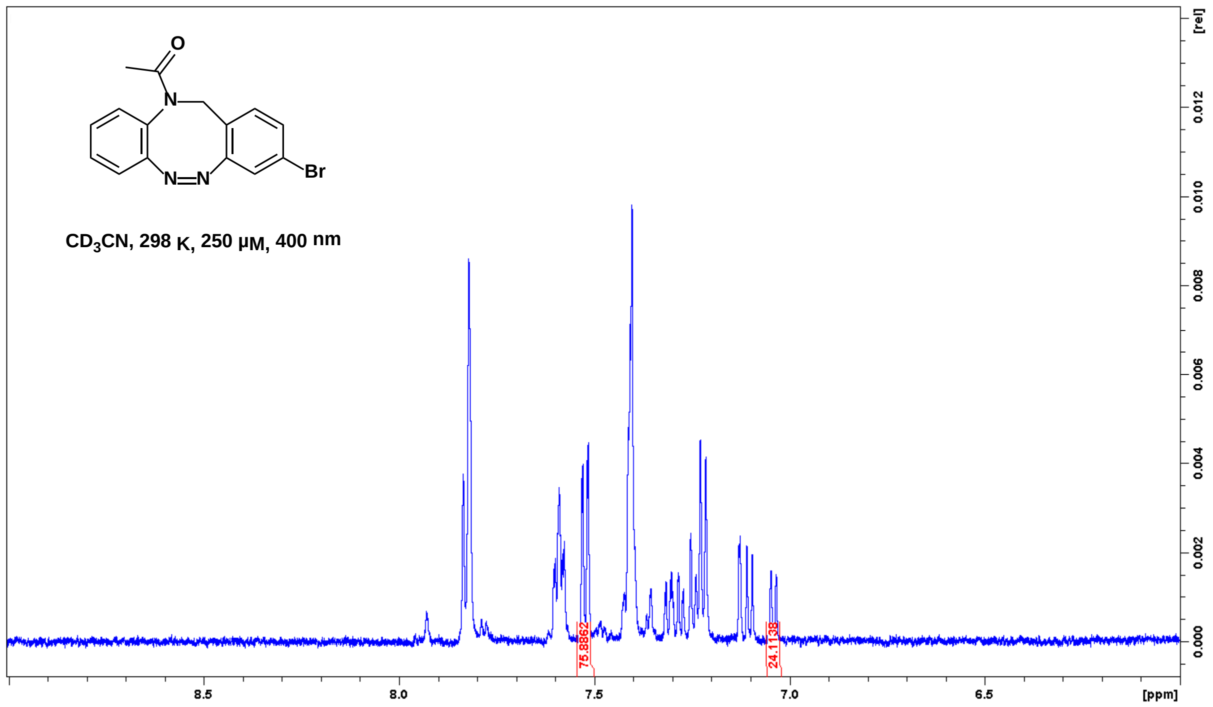

Figure S55: ^{1}H NMR spectrum (aromatic region) of Br-NAc diazocine **10a** in CD_3CN at 298 K after irradiation with 400 nm.

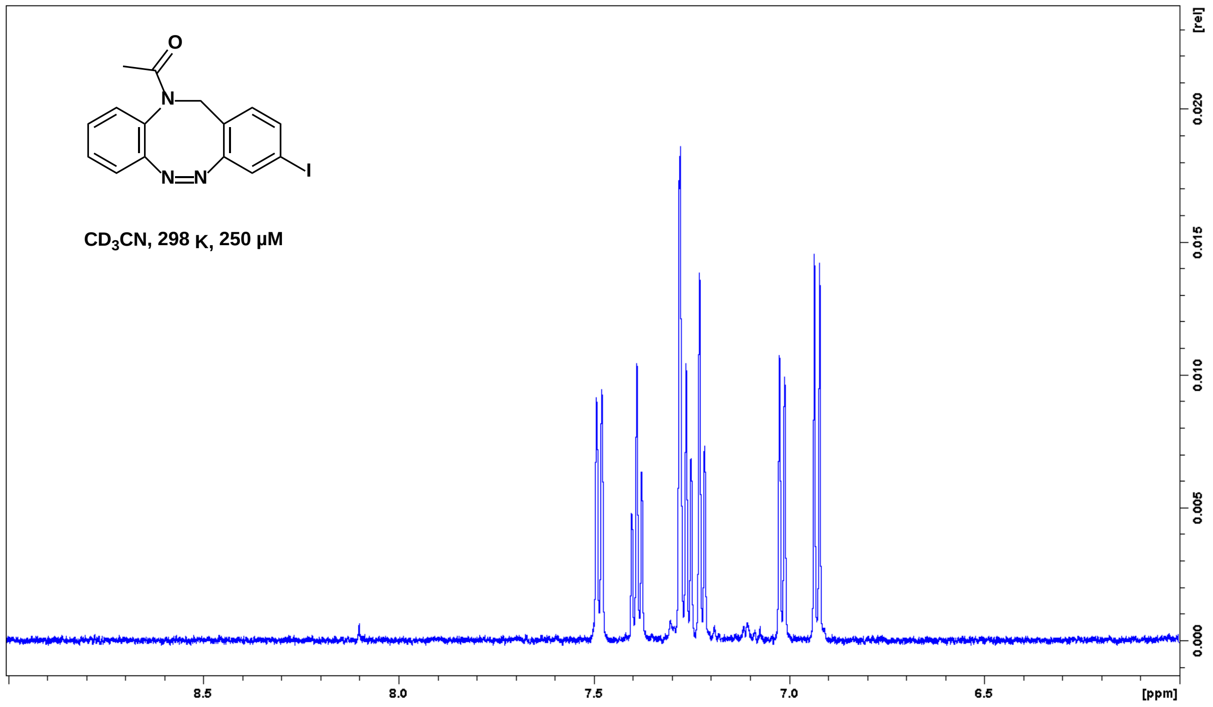

Figure S56: ^{1}H NMR spectrum (aromatic region) of I-NAc diazocine **10b** in CD_3CN at 298 K.

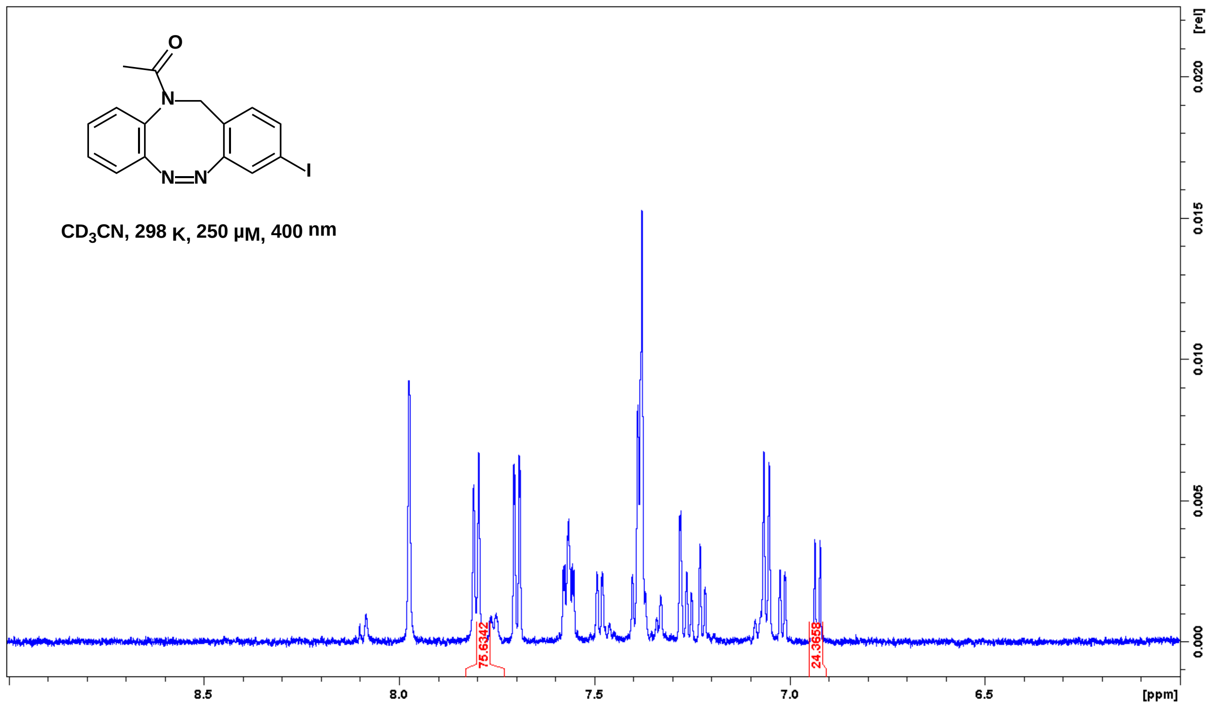

Figure S57: ^{1}H NMR spectrum (aromatic region) of I-NAc diazocine **10b** in CD_3CN at 298 K after irradiation with 400 nm.

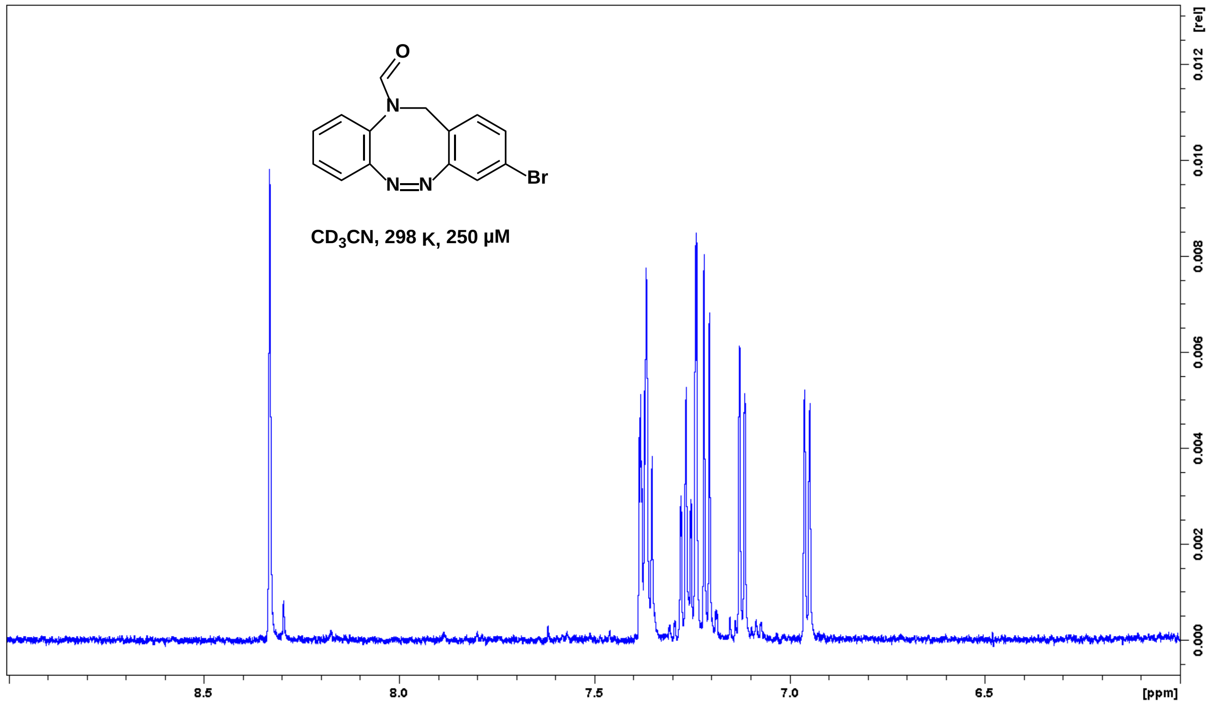

Figure S58: ^{1}H NMR spectrum (aromatic region) of Br-NCHO diazocine **11a** in CD_3CN at 298 K.

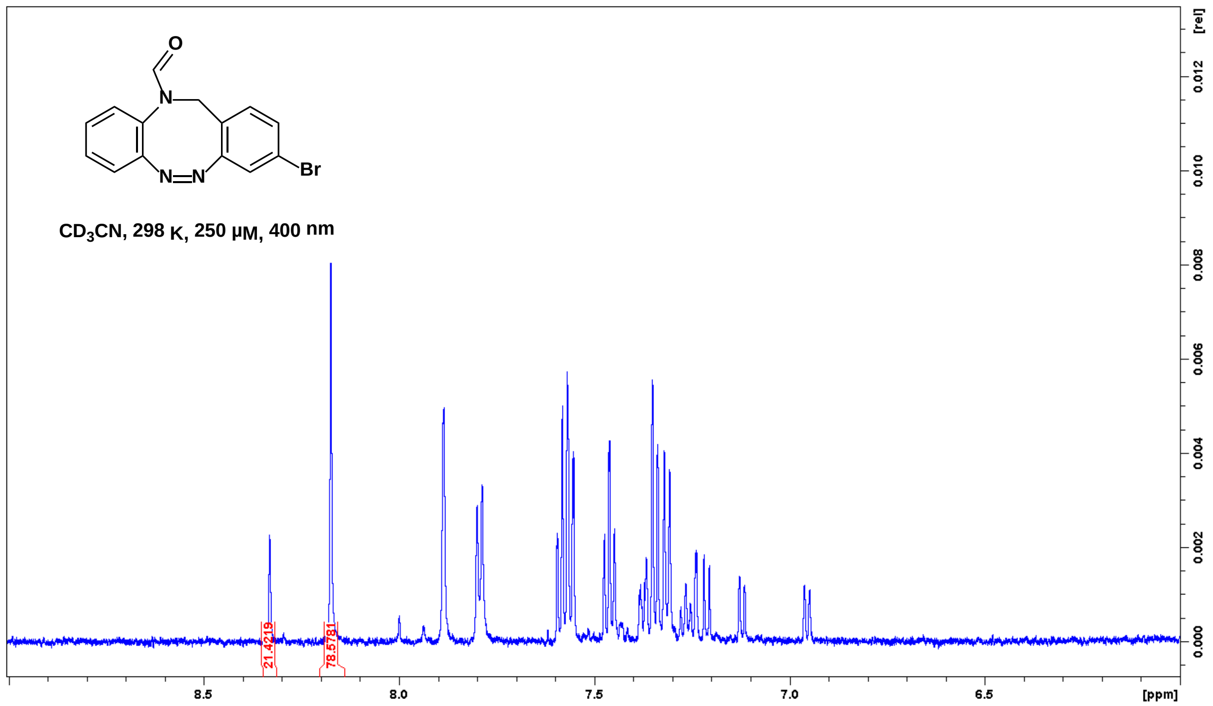

Figure S59: ^{1}H NMR spectrum (aromatic region) of Br-NCHO diazocine **11a** in CD_3CN at 298 K after irradiation with 400 nm.

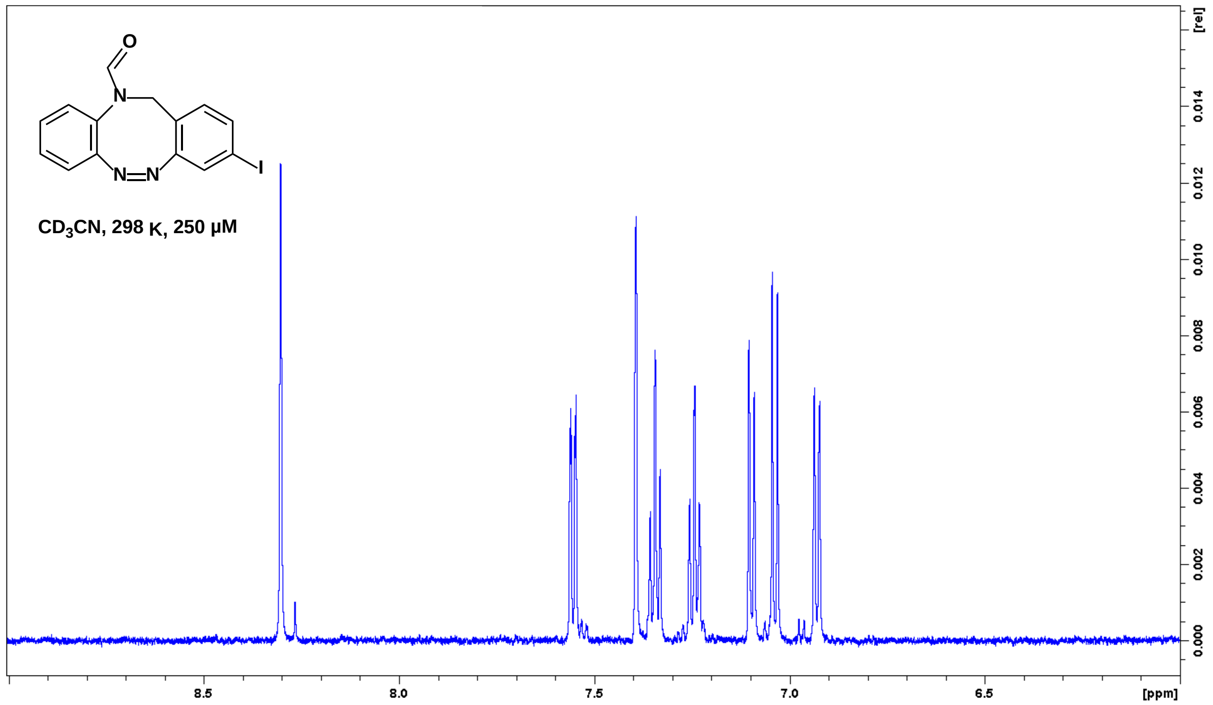

Figure S60: ^{1}H NMR spectrum (aromatic region) of I-NCHO diazocine **11b** in CD_3CN at 298 K.

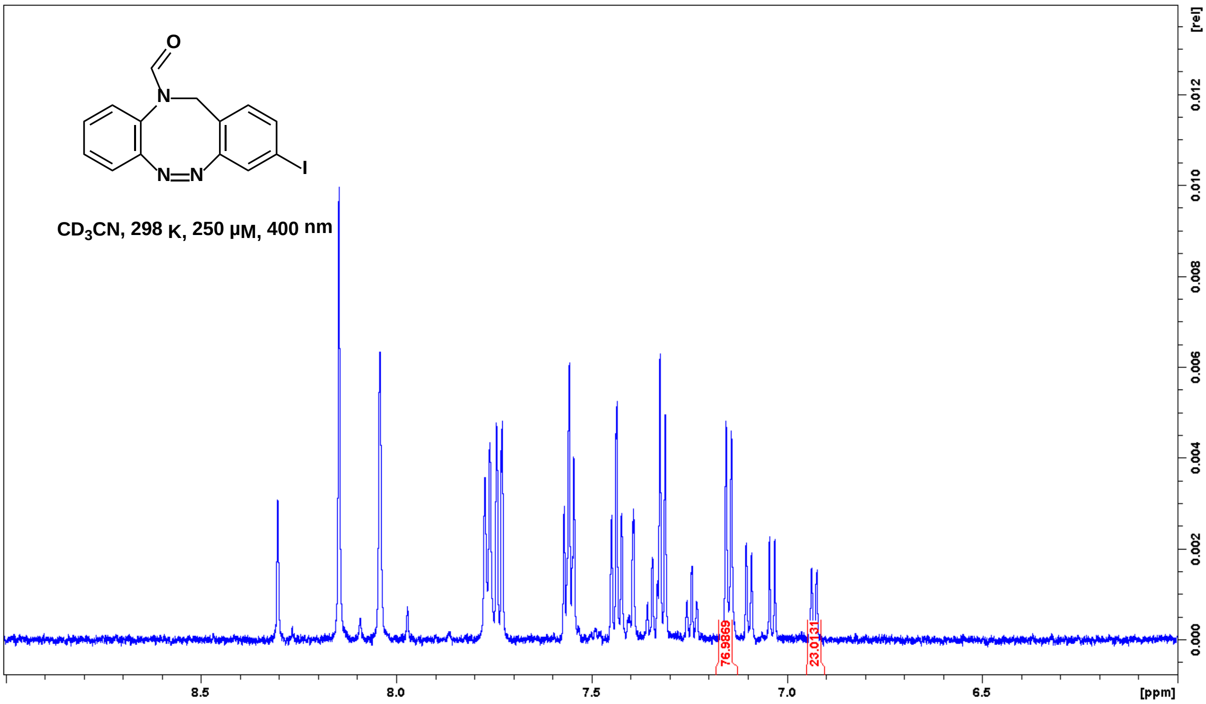

Figure S61: ^{1}H NMR spectrum (aromatic region) of I-NCHO diazocine **11b** in CD_3CN at 298 K after irradiation with 400 nm.

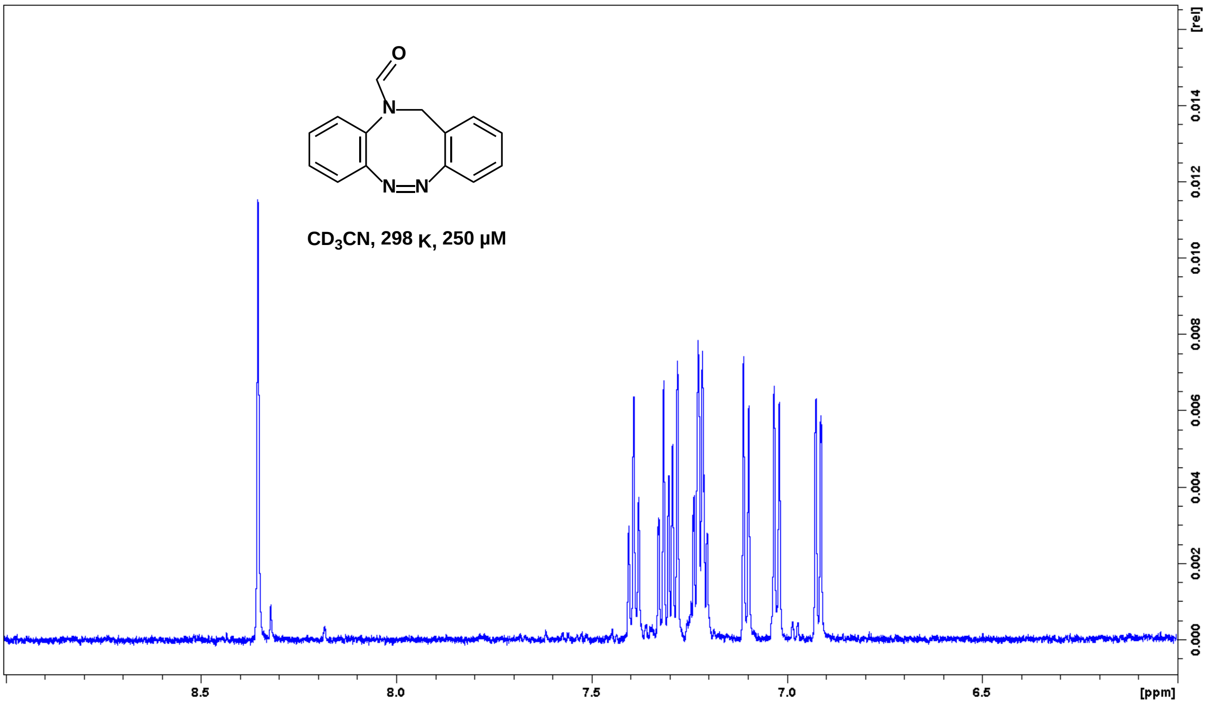

Figure S62: ^{1}H NMR spectrum (aromatic region) of NCHO diazocine **11c** in CD_3CN at 298 K.

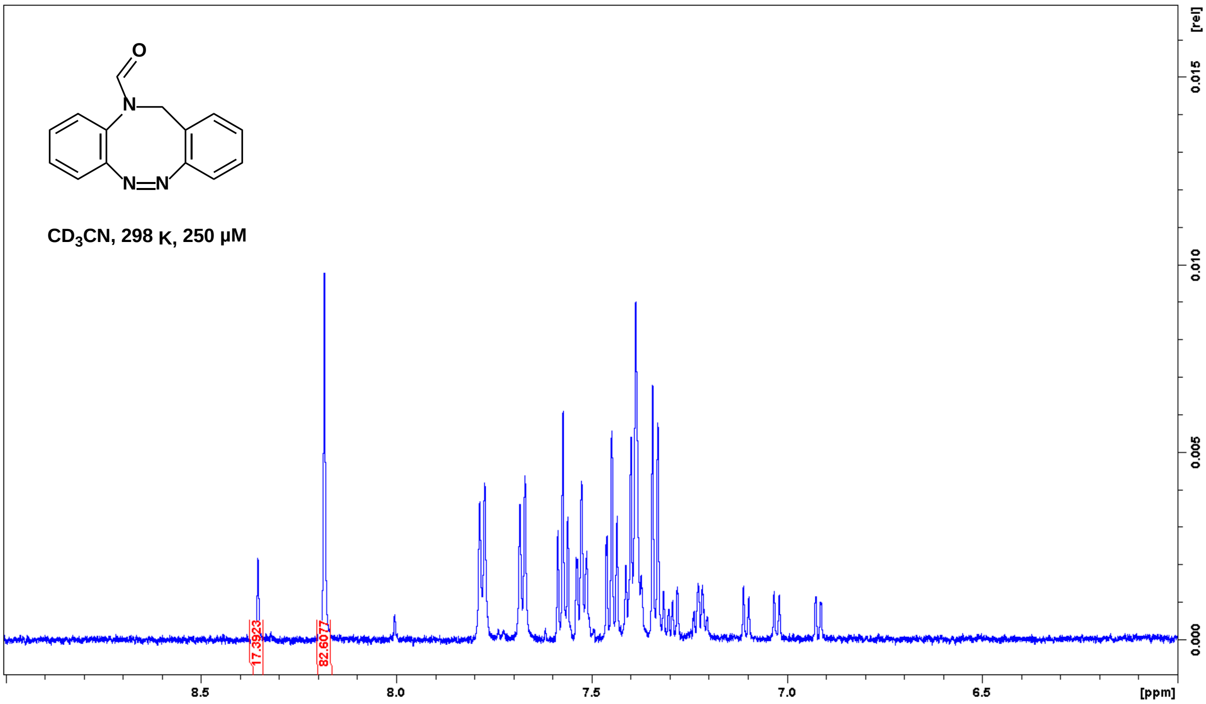

Figure S63: ^{1}H NMR spectrum (aromatic region) of NCHO diazocine **11c** in CD_3CN at 298 K after irradiation with 400 nm.

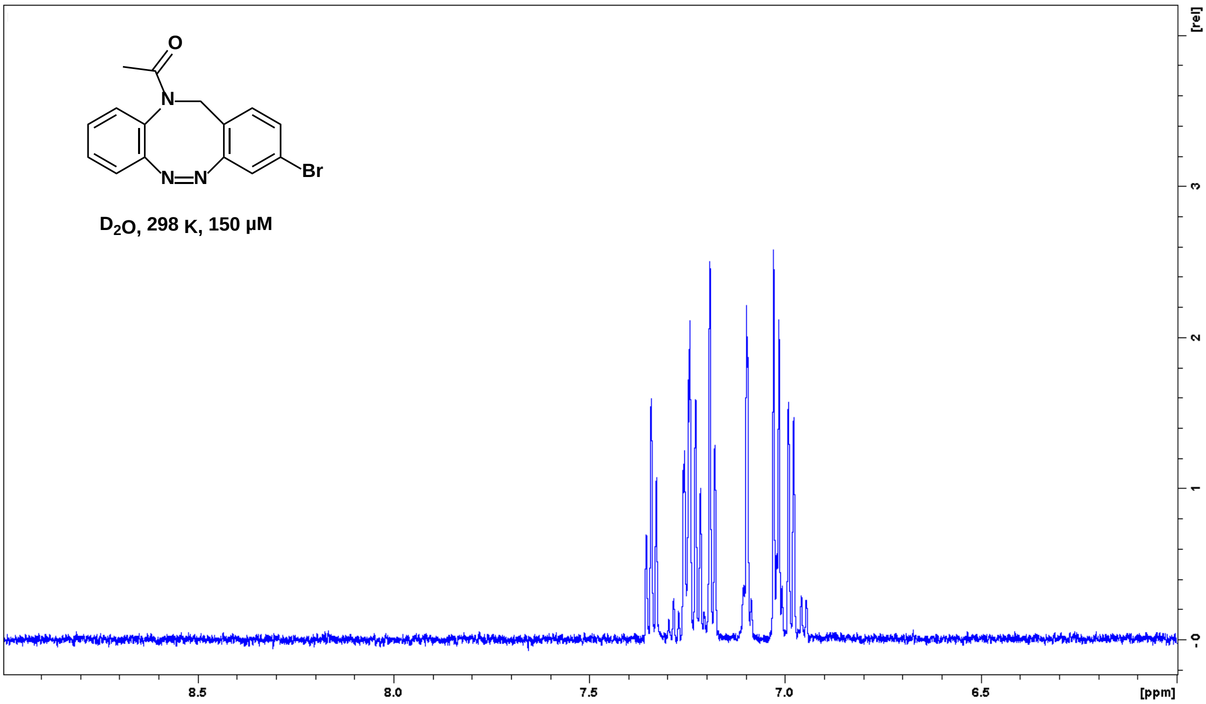

Figure S64: ^{1}H NMR spectrum (aromatic region) of Br-NAc diazocine **10a** in D_2O at 298 K.

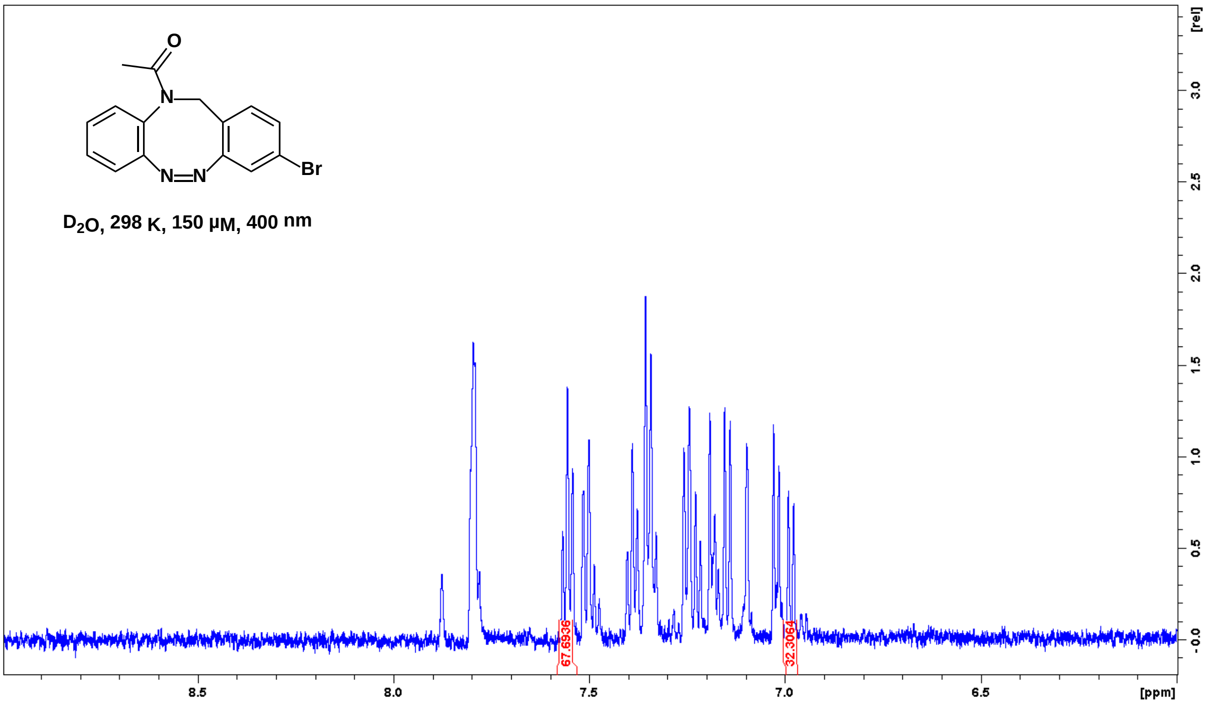

Figure S65: ^{1}H NMR spectrum (aromatic region) of Br-NAc diazocine **10a** in D_2O at 298 K after irradiation with 400 nm.

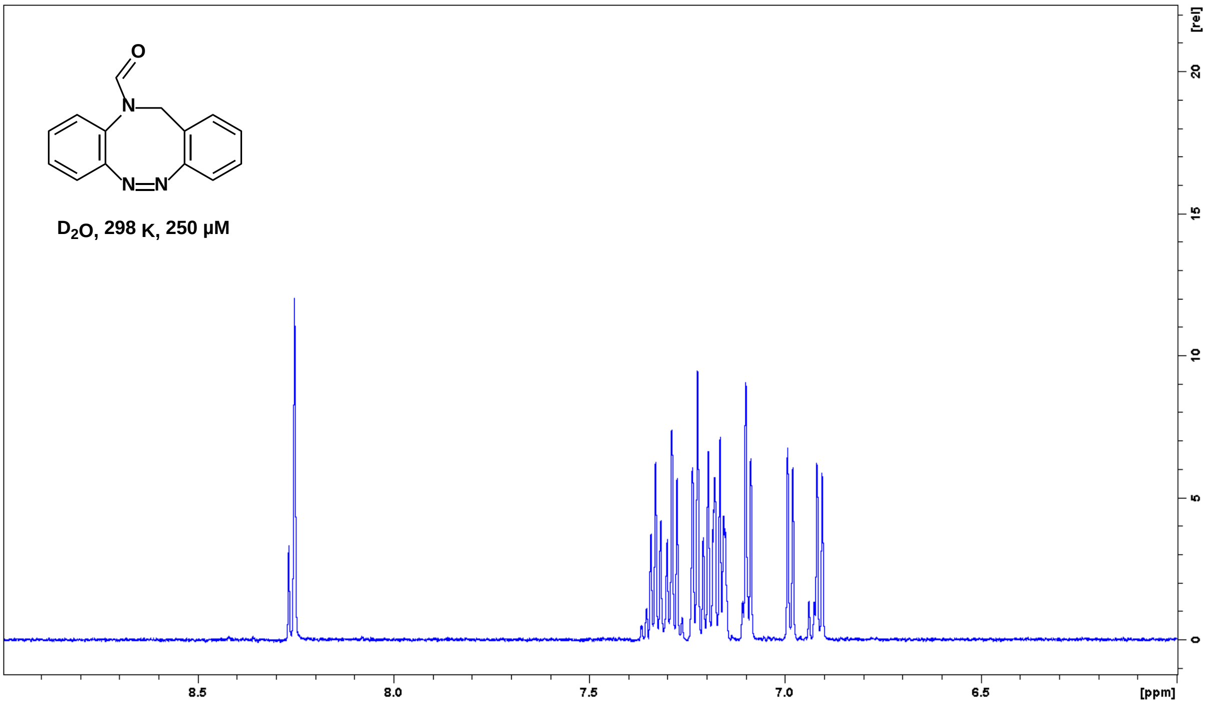

Figure S66: ^{1}H NMR spectrum (aromatic region) of NCHO diazocine **11c** in D_2O at 298 K.

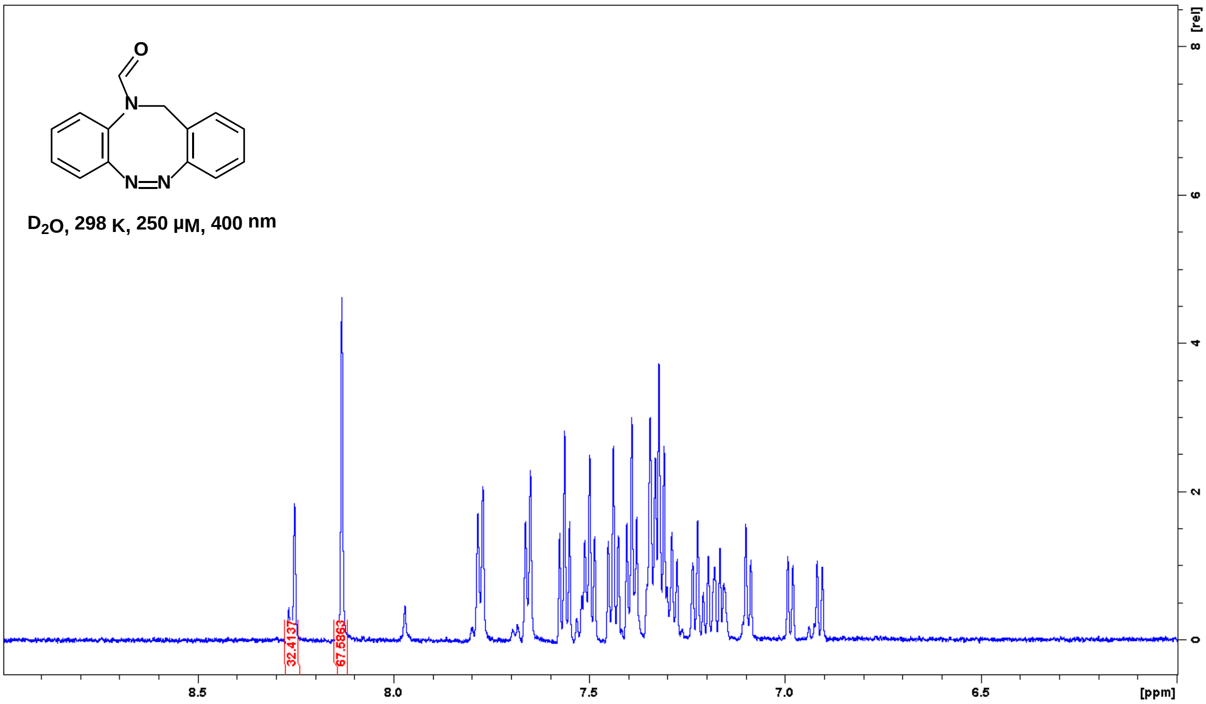

Figure S67: ^{1}H NMR spectrum (aromatic region) of NCHO diazocine **11c** in D_2O at 298 K after irradiation with 400 nm.

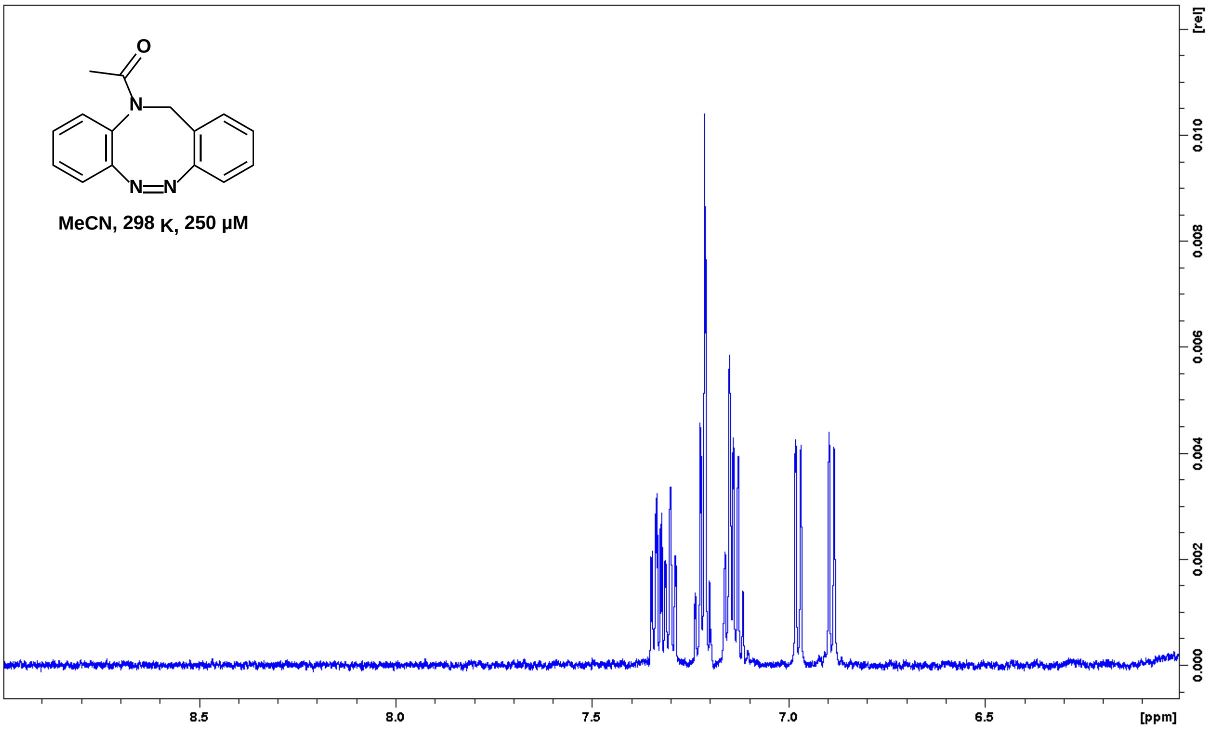

Figure S68: ^{1}H NMR spectrum (aromatic region) of NAc diazocine **10c** in MeCN at 298 K.

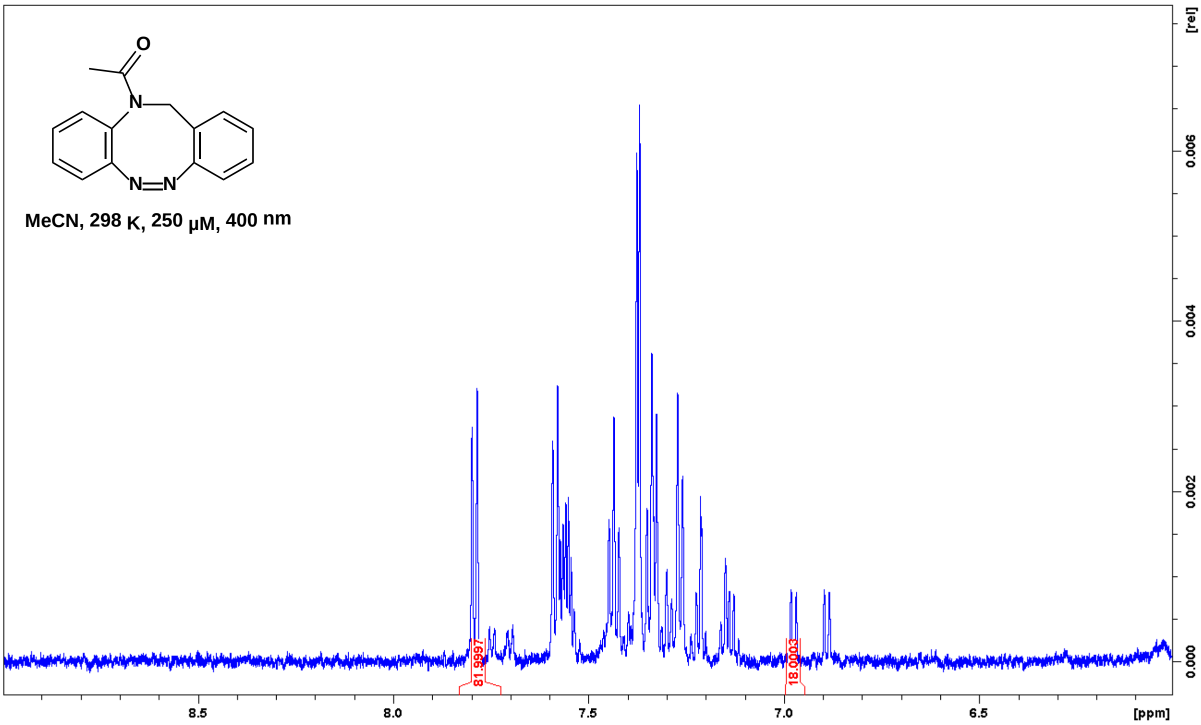

Figure S69: ^{1}H NMR spectrum (aromatic region) of NAc diazocine **10c** in MeCN at 298 K after irradiation with 400 nm.

[1] Lentes, P.; Stadler, E.; Röhricht, F.; Brahms, A.; Gröbner, J.; Sönnichsen, F. D.; Gescheidt, G.; Herges, R. *J. Am. Chem. Soc.* 2019, 141, 34, 13592–13600.
[2] Liebig, J. *Justus Liebigs Ann. Chem.* **1832**, *1*, 182–230.
[3] Schehr, M.; Hugenbusch, D.; Moje, T.; Näther, C.; Herges, R. *Beilstein J. Org. Chem.* **2018**, *14*, 2799–2904.
